# Hijacking the Hijackers: *Escherichia coli* Pathogenicity Islands Redirect Helper Phage Packaging for Their Own Benefit

**DOI:** 10.1016/j.molcel.2019.06.017

**Published:** 2019-09-05

**Authors:** Alfred Fillol-Salom, Julio Bacarizo, Mohammed Alqasmi, J. Rafael Ciges-Tomas, Roser Martínez-Rubio, Aleksander W. Roszak, Richard J. Cogdell, John Chen, Alberto Marina, José R. Penadés

**Affiliations:** 1 Institute of Infection, Immunity and Inflammation, University of Glasgow, Glasgow G12 8TA, UK; 2 College of Applied Medical Sciences, Shaqra University, Shaqra City 15572, Saudi Arabia; 3 Instituto de Biomedicina de Valencia (IBV-CSIC) and CIBER de Enfermedades Raras (CIBERER), Valencia, Spain; 4 Departamento de Ciencias Biomédicas, Universidad CEU Cardenal Herrera, 46113 Moncada, Spain; 5 Institute of Molecular Cell and Systems Biology, University of Glasgow, Glasgow G12 8QQ, UK; 6 Department of Microbiology and Immunology, Yong Loo Lin School of Medicine, National University of Singapore, 5 Science Drive 2, Singapore, Singapore; 7 MRC–University of Glasgow Centre for Virus Research, Glasgow G61 1QH, UK

**Keywords:** bacteriophage, pathogenicity islands, pirating, PICI, evolution, virulence, gene transfer, transduction, TerS

## Abstract

Phage-inducible chromosomal islands (PICIs) represent a novel and universal class of mobile genetic elements, which have broad impact on bacterial virulence. In spite of their relevance, how the Gram-negative PICIs hijack the phage machinery for their own specific packaging and how they block phage reproduction remains to be determined. Using genetic and structural analyses, we solve the mystery here by showing that the Gram-negative PICIs encode a protein that simultaneously performs these processes. This protein, which we have named Rpp (for redirecting phage packaging), interacts with the phage terminase small subunit, forming a heterocomplex. This complex is unable to recognize the phage DNA, blocking phage packaging, but specifically binds to the PICI genome, promoting PICI packaging. Our studies reveal the mechanism of action that allows PICI dissemination in nature, introducing a new paradigm in the understanding of the biology of pathogenicity islands and therefore of bacterial pathogen evolution.

## Highlights

• PICI Rpp protein promotes PICI transfer while blocking phage reproduction
• Rpp forms a heterocomplex with helper phage TerS
• Crystal structures of Rpp alone or complexed with TerS were determined
• TerS complexed with Rpp switches specificity from the phage DNA to the PICI genome

## Graphical Abstract

**Figure:**
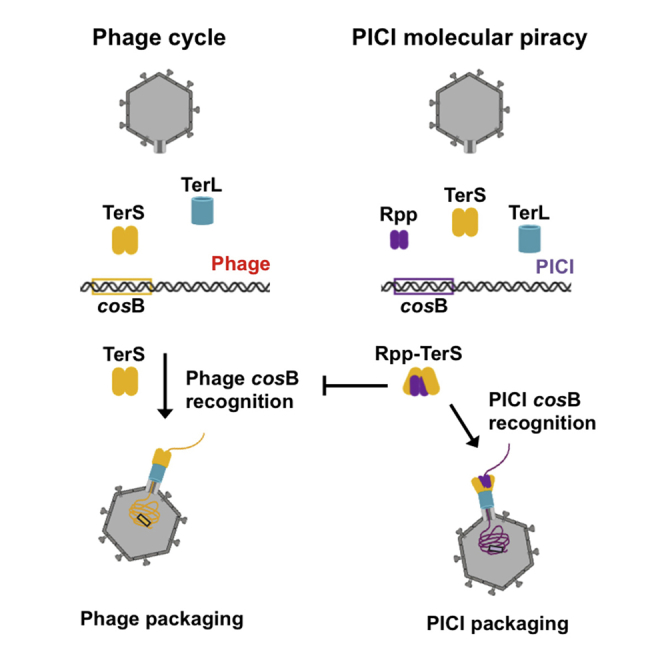

## Teaser

Fillol-Salom et al. report that Gram-negative PICIs employ the Rpp protein to block helper phage reproduction. Rpp binds to helper phage TerS, and the Rpp-TerS heterocomplex enables PICIs to hijack the phage machinery for their own packaging. These findings reveal a mechanism used by Gram-negative PICIs to spread in nature.

## Introduction

The acquisition of mobile genetic elements (MGEs) that carry virulence factors is a major event that can transform an avirulent or weakly virulent strain into a multi-resistant hypervirulent strain. In spite of the importance of their consequences, the mechanisms underlying the genetic transfer of pathogenicity islands among bacteria remain unidentified in most cases. In recent years, we have described and characterized a new class of chromosomally integrated mobile pathogenicity islands: the phage-inducible chromosomal islands (PICIs) (Penadés and Christie, 2015). The PICIs are widespread among Gram-positive cocci and Gram-negative bacteria (Fillol-Salom et al., 2018, Martínez-Rubio et al., 2017), and they are clinically relevant because they carry and disseminate genes for bacterial superantigens, virulence, and antibiotic resistance (Penadés and Christie, 2015). Following induction by a helper phage, PICIs excise from the bacterial chromosome, replicate, and are packaged into phage-like particles composed of phage virion proteins, leading to very high frequencies of intra- as well as inter-generic transfer (Chen et al., 2015, Chen and Novick, 2009, Maiques et al., 2007).

Although the biology of the Gram-positive PICIs has been extensively studied (Penadés and Christie, 2015), it remains a mystery how the PICI elements present in the Gram-negative bacteria hijack the phage machinery for their preferential packaging and transfer in nature and how these elements interfere with helper phage reproduction. To address these questions, we have analyzed one of these elements, EcCICFT073, present in the uropathogenic *Escherichia coli* CFT073 strain. This PICI raised our curiosity because of its role in virulence and because this element can be mobilized by the archetypical *E. coli* λ and 80 phages (Fillol-Salom et al., 2018). More importantly, our previous results had demonstrated that EcCICFT073 can interfere with phage reproduction using a novel mechanism of phage interference. Although most Gram-positive PICIs interfere with phage reproduction by promoting the formation of small PICI capsids that are much too small for the larger phage genomes (Carpena et al., 2016, Martínez-Rubio et al., 2017, Matos et al., 2013, Quiles-Puchalt et al., 2014, Ruzin et al., 2001, Ubeda et al., 2005), that was not the case for the EcCICFT073 element, which is packaged into phage-sized capsid (Fillol-Salom et al., 2018).

How do *cos* phages, such as λ and 80, package their DNA? The terminase of phage λ is among the best biochemically characterized proteins that catalyze this process and provides an ideal model for DNA packaging. The enzyme is a hetero-oligomer composed of gpNu1 (also called small terminase [TerS]) and gpA (large terminase [TerL]) subunits. Genome packaging begins with terminase assembly at *cos*, the packaging initiation site in the DNA concatemer. The λ *cos* sequence has three regions required to interact with the packaging machinery: *cos*Q; *cos*N; and *cos*B. Termination of phage packaging requires *cos*Q, and TerL completes this process by cutting the DNA at *cos*N. Initiation of DNA packaging requires both *cos*N and *cos*B sites; *cos*B consists of three binding sites or R elements (R3, R2, and R1) that are required for λ TerS binding to initiate the phage packaging process (Rao and Feiss, 2008; Figure S1).

In a previous study, we found that EcCICFT073 requires the phage-encoded TerS for packaging. We also demonstrated that EcCICFT073 carries two *cos* sites, *cos*1 and *cos*2, with *cos*1 being required for the λ- and 80-mediated transfer of the element (Fillol-Salom et al., 2018). Surprisingly, although both EcCICFT073 *cos* sites have *cos*Q and *cos*N sequences that resemble those present in the *E. coli* λ and 80 phages, we were unable to identify the phage *cos*B element in the EcCICFT073 region (Figure S1A; Fillol-Salom et al., 2018). This observation posed the question, if EcCICFT073 requires the phage machinery for packaging, why does it carry a different *cos*B sequence in its genome, which would be poorly recognized by the phage TerS protein? Does EcCICFT073 encode uncharacterized proteins involved in PICI packaging? And how does EcCICFT073 interfere with phage reproduction? We have unraveled here the mechanism of molecular piracy used by the *E. coli* PICIs to be highly and preferentially packaged and transferred in nature.

## Results

### Identification of the EcCICFT073-Encoded Inhibitor of Phage λ Reproduction

**Figure 1:**
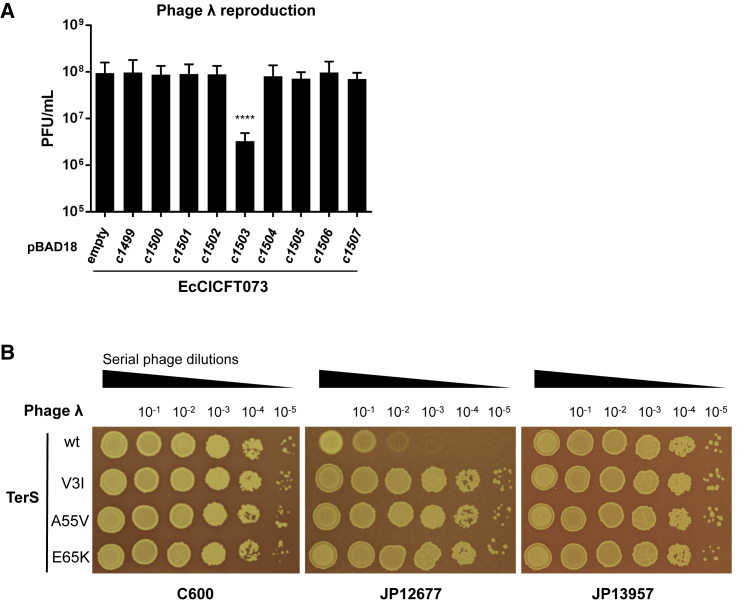
Identification of the EcCICFT073-Encoded Protein Involved in Phage Interference (A) Phage λ was used to plaque derivatives of non-lysogenic *E. coli* laboratory strain 594 containing pBAD18 expressing different EcCICFT073 proteins. Infected cells were plated on phage base agar supplemented with 0.1% arabinose using phage top agar. The results are represented as the plaque forming units (PFUs) mL^−1^. The means and SDs are presented (n = 3). A one-way ANOVA with Dunnett’s multiple comparisons test was performed to compare mean differences pBAD18 derivatives. Adjusted p values were as follows: ^∗∗∗∗^p < 0.0001. (B) Phage λ dilutions were spotted on non-lysogenic *E. coli* laboratory strain C600, JP12677 (C600 EcCICFT073 *tet*A-positive), or JP13957 (C600 EcCICFT073 *tet*A-positive Δ*c1503*). Plates were stained with 0.1% (w/v) 2,3,5-triphenyltetrazolium chloride (TTC) for enhanced contrast. See also Figures S1 and S2 and Table S1.

We initiated this study by identifying the EcCICFT073 gene responsible for blocking λ reproduction. To do that, we individually cloned the EcCICFT073 genes present in the region located after the EcCICFT073 *ori* site into the expression vector pBAD18 (Guzman et al., 1995), under the control of the arabinose-inducible promoter (*P*_BAD_), and tested these clones for inhibition of λ reproduction. Note that, in the Gram-positive PICIs, this region usually contains the genes involved in phage interference. Expression of the *c1503* gene, but none of the other genes, dramatically reduced plaque formation by phage λ (Figure 1A). Similar results were obtained with phage 80, but not with phage HK97, which is insensitive to *c1503* (Figure S2A). Deletion of *c1503* fully restored plaque formation and increased λ plaque titers nearly to those seen with the host strain lacking EcCICFT073 (Figure 1B). Based on its function (redirecting phage packaging; see below for more details), the *c1503*-encoded protein was named RppA.

### Identification of RppA Homologs

We analyzed the distribution of the *rpp*A gene in the GenBank database and observed that many *E. coli* PICIs encode *rpp*A homologs (Figure S1B), as well as PICIs from other species (Table S1). We next examined whether the other two Rpp homologs found in *E. coli* PICIs, named here RppB and RppC, respectively (Table S1; Figures S1C and S1D), were also able to block phage reproduction. Note that RppA and RppB show 82.64% identity, although RppC shows less identity (43.65%) with RppA and is longer in length (144 residues RppA versus 153 residues RppC; Figures S1C and S1D). The *rpp*B and *rpp*C genes were cloned into the expression vector pBAD18, and the ability of the two encoded proteins to block phage reproduction was tested as previously indicated. As shown in Figure S2B, both RppB and RppC also blocked λ and 80 reproduction.

### Identification of the Phage-Encoded Protein Targeted by the Rpp Proteins

We next attempted to identify the stage in the phage reproduction cycle inhibited by RppA. To do this, we introduced the pBAD18 derivative plasmid expressing RppA into the λ and 80 lysogens, and the life cycle of these prophages was induced using mitomycin C (MC). To express RppA, the culture media was supplemented with 0.02% arabinose. Samples containing the lysogenic *E. coli* cells were taken before and 30, 60, 90, and 120 min after MC induction, total DNA was extracted, and the phage replication was analyzed by Southern blotting. In parallel, the impact of RppA on the phage titers was also analyzed. As expected, RppA expression significantly reduced phage titers (Figure S3A), but it did not impact phage replication or phage lysis (Figures S3B and S3C), suggesting that RppA targets phage packaging.

To identify the λ phage protein that was targeted by RppA, we isolated phage mutants able to form plaques on a strain either carrying the EcCICFT073::*tet*A element or expressing the cloned *rpp*A gene. Mutants were readily obtained, and we sequenced the phage genome of 6 of these. In parallel, we also isolated 80 phage mutants insensitive to the RppA interference and sequenced 3 of these mutants. In each case, there was an amino acid substitution in the gene encoding the TerS subunit (Table 1); in four of the six λ TerS mutants, the mutations were at the same site, E65, and in all of these, the glutamic acid was replaced by lysine (E65K; Table 1). Additional mutations were found in the *ter*S gene from phages λ and 80 (Table 1), suggesting that RppA interacts with multiple TerS residues. The identification of TerS as the target explains why the phage HK97 is insensible to RppA; although phages λ and 80 encode a TerS that is practically identical (GenPept: NP_040580 and AFV29141, respectively), the HK97 encoded TerS is completely different in sequence (GenPept: NP_037697).

**Table 1:** Phage Mutants Insensitive to the Rpp-Mediated Interference

	TerS Mutations	
	Phage λ	Phage 80
Target Used to Evolve the Phages		
EcCICFT073::*tet*A	V3I, A55V, E65K	
pBAD18 *rpp*A	E65K	D68G, L69R, R70P
pBAD18 *rpp*C	E65K/Y50N	E65K

To know whether RppC also targets the phage TerS, we tried to isolate λ phage mutants insensitive to RppC. Surprisingly, we were unable to isolate a λ mutant capable to form plaques on the RppC-positive strain. We then repeated the selection using phage 80. A single-phage mutant, insensitive both to RppC and RppA, was obtained. This phage carried the TerS E65K mutation (Table 1; Figure S2C). This result confirmed that RppC also targets the phage TerS protein and suggested that the affinity of the RppC protein for the λ TerS is stronger than that observed for the RppA protein. If this was the case, only those λ phages carrying several mutations on the TerS protein would be able to escape to the RppC interference. To test this, we made use of the aforementioned λ TerS E65K mutant, which is insensitive to RppA but still sensitive to RppC (Figure S2D) and evolved it in presence of RppC. In support of this idea, we obtained λ mutants insensitive to RppC; these phages had the double Y50N and E65K mutations in the λ TerS (Table 1; Figure S2D). The fact that the 80 and λ TerS have some differences in sequence explains why one single mutation is enough to avoid RppC interference in phage 80 but two mutations are required in phage lambda.

**Figure 2:**
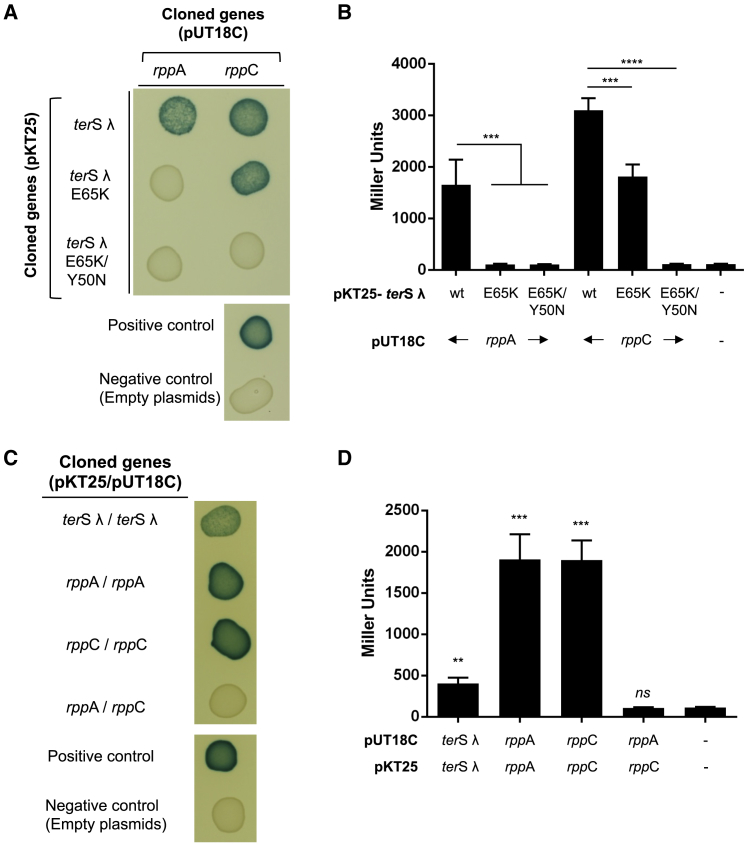
Characterization of the λ TerS-Rpp Interaction (A) Bacterial adenylate cyclase-based two-hybrid (BACTH) analysis was performed using the plasmid pKT25 encoding different λ TerS versions (WT, E65K, and E65K/Y50N) and plasmid pUT18C encoding RppA or RppC. Plasmid combinations are indicated. (B) Quantification of the BACTH analysis in (A) after overnight induction with 0.5 mM Isopropyl β-D-1-thiogalactopyranoside (IPTG) measured in Miller units. The means of results and SD are presented (n = 3). A one-way ANOVA with Dunnett’s multiple comparisons test was performed to compare mean differences between samples. Adjusted p values were as follows: *rpp*A-WT versus *rpp*A-E65K ^∗∗∗^p = 0.0009, *rpp*A-WT versus *rpp*A-E65K ^∗∗∗^p = 0.0010, and *rpp*C-WT versus *rpp*C-E65K ^∗∗∗^p = 0.0003; ^∗∗∗∗^p < 0.0001. (C) The Rpp proteins form dimers. BACTH analysis was performed using plasmids pKT25 and pUT18C encoding λ TerS, RppA, or RppC. (D) Quantification of the BACTH analysis in (C) after overnight induction with 0.5 mM IPTG measured in Miller units. The means of results and SD are presented (n = 3). An unpaired t test was performed to compare dimerization against empty plasmids. Adjusted p values were as follows: ^∗∗^p = 0.0024; *rpp*A ^∗∗∗^p = 0.0005, *rpp*C ^∗∗∗^p = 0.0002. *ns*, not significant. See also Figures S2, S3, S4, S5, and S8.

The previous results suggested that the Rpp proteins interact with the phage-encoded TerS. This was confirmed using a bacterial two-hybrid test, comparing the wild-type (WT) λ TerS and the λ TerS E65K and TerS Y50N/E65K mutants for any interaction with RppA or RppC. As shown in Figure 2, RppA binds strongly to the WT λ TerS, but not to the mutant proteins, and RppC binds even more strongly to the λ TerS and also binds to the λ TerS E65K protein, but not to the λ TerS Y50N/E65K double-mutant protein, confirming that RppC has higher affinity for the λ TerS than RppA. An explanation for this is provided later. Remarkably, both RppA and RppC proteins produce dimers, as does the λ TerS (Figure 2; de Beer et al., 2002). However, RppA and RppC do not interact with each other (Figure 2), suggesting that the different islands interfere with the helper phage, but not with one another. Identical results were obtained when the interaction between the Rpp proteins and the WT and mutant 80 TerS proteins were analyzed (Figure S4). Finally, the interaction between RppC and λ TerS was confirmed by a pull-down assay using His_6_-tagged λ TerS (residues 1–98) and untagged RppC (Figure S5A), suggesting that the Rpp proteins interact directly and specifically with the phage-encoded TerS, blocking packaging of the phage DNA.

### RppA Is Required for EcCICFT073 Packaging and Transfer

**Figure 3:**
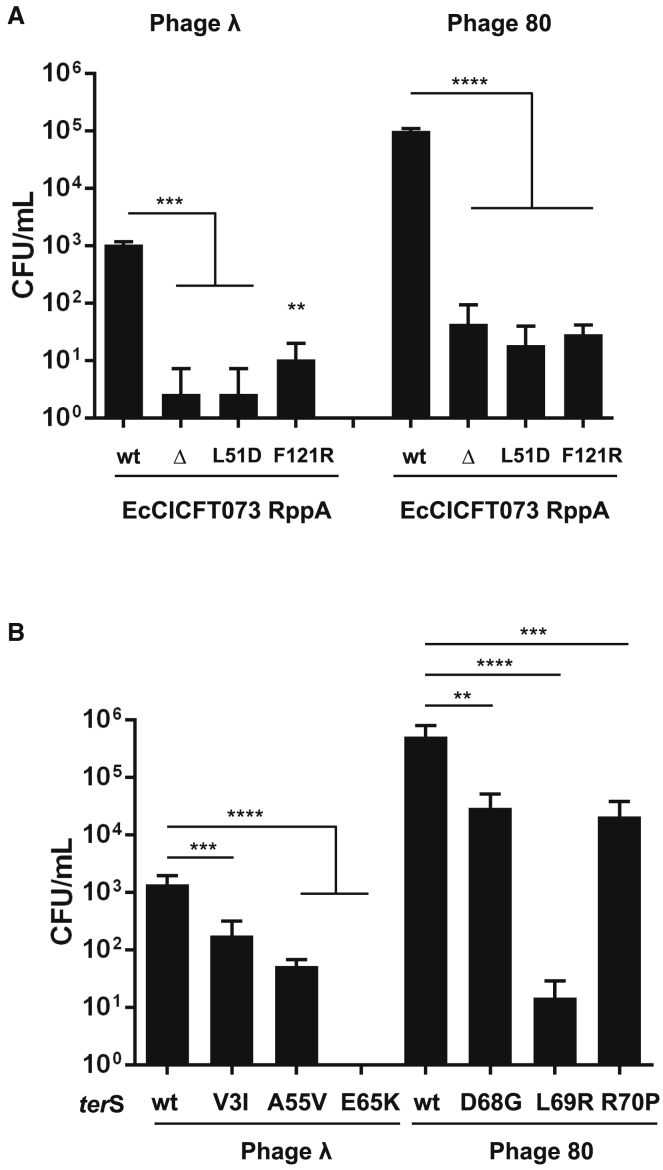
Effect of RppA and TerS Mutations on EcCICFT073 Transfer (A) Lysogenic strains for phages λ or 80, carrying different versions of the EcCICFT073 island (WT or encoding different RppA mutants), were MC induced (2 μg/mL), and the transfer of the PICI was analyzed using *E. coli* 594 as the recipient strain. The means of the colony forming units (CFUs) and SD are presented (n = 3). A one-way ANOVA with Dunnett’s multiple comparisons test was performed to compare mean differences between EcCICFT073-*rpp*A WT. Adjusted p values were as follows: ^∗∗∗^p = 0.0005; ^∗∗^p = 0.0018; and ^∗∗∗∗^p < 0.0001. (B) Lysogenic strains, carrying either WT or mutant λ or 80 prophages, were MC induced in presence of EcCICFT073 and the transfer of the island analyzed using *E. coli* 594 as recipient strain. The means of the CFUs and SD are presented (n = 3). A one-way ANOVA with Dunnett’s multiple comparisons test was performed to compare mean differences within between empty plasmids. Adjusted p values were as follows: Lambda TerS WT versus V3I ^∗∗∗^p = 0.0001; Phage 80 TerS WT versus D68G ^∗∗^p = 0.0016; and Phage 80 TerS WT versus R70P ^∗∗∗^p = 0.0007, ^∗∗∗∗^p < 0.0001. See also Figures S4 and S6.

The previous results were unexpected. Because EcCICFT073 requires the helper phage TerS protein for packaging (Fillol-Salom et al., 2018), why does this island express a protein (RppA) that blocks TerS activity? Trying to solve this question, we analyzed the role of RppA in EcCICFT073 transfer. Because RppA blocks TerS and TerS is required for EcCICFT073 transfer, we hypothesized that deletion of *rpp*A would increase EcCICFT073 packaging and transfer by phages λ or 80. This was not the case, and surprisingly, the transfer of the *rpp*A mutant island by phages λ and 80 was significantly reduced compared to the transfer of the WT island (Figure 3A).

We next tested the ability of different λ and 80 TerS mutants, incapable of interacting with the Rpp proteins and consequently insensitive to Rpp-mediated interference (Figures 1B, 2, and S4), to transfer EcCICFT073. As shown in Figure 3B, all the evolved phage mutants showed a reduced capacity to package and transfer the island, suggesting that the Rpp-TerS interaction is essential for EcCICFT073 transfer. Collectively, these results can be summarized as follows: (1) although the phage TerS is required for EcCICFT073 transfer, this island expresses RppA, a protein that blocks TerS function; (2) in addition to blocking phage packaging, RppA is essential for EcCICFT073 packaging; and (3) to perform its function, RppA must interact with the phage-encoded TerS. Based on these data, the pertinent question was how do the Rpp proteins work?

### Structure of RppC

**Figure 4:**
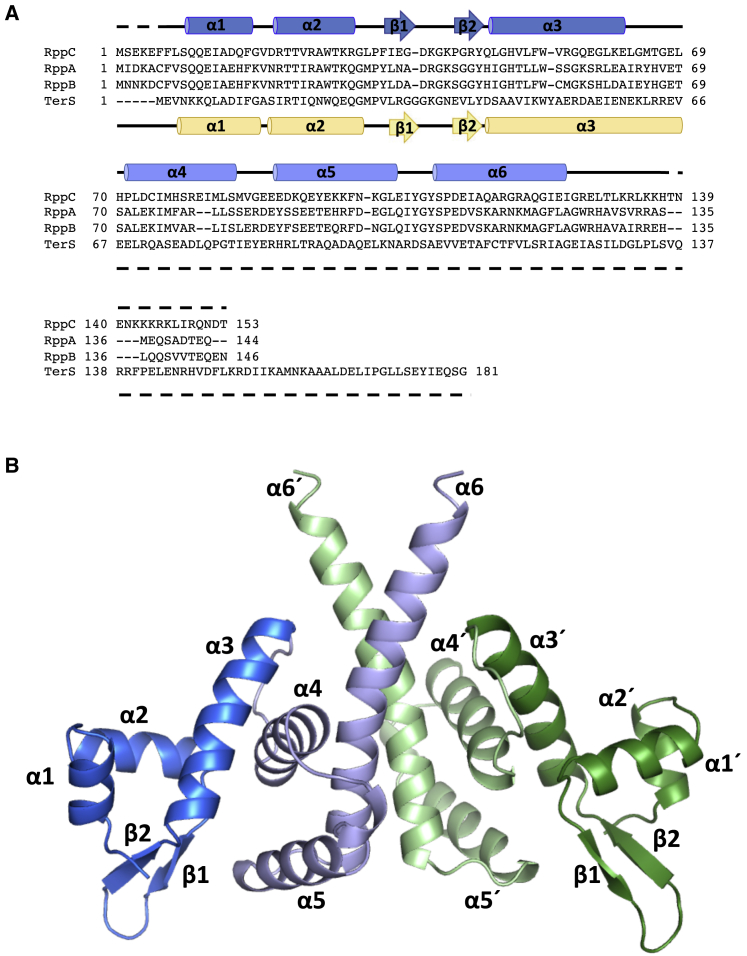
Crystallographic Structure of RppC (A) Sequence alignment of Rpp proteins and λ TerS. Structural elements of RppC are shown above the sequence colored in blue tones. Structural elements of TerS (PDB: 1J9I) are shown below the sequence colored in yellow. (B) Cartoon representation of the RppC dimer. Each monomer is colored in blue and green, respectively. DNA binding motifs are highlighted in dark tones. Secondary structural elements are numbered and labeled in order from N to C terminus. The apostrophe (') indicates the elements from the second protomer. See also Figure S5 and Table S2.

To address this question, we first solved the structure of RppC at 2.4 Å resolution by X-ray crystallography, using the single-wavelength anomalous dispersion (SAD) method (Table 2). The structure showed a single molecule in the asymmetric unit that forms a dimer due to the symmetry of the crystal packing (Figure 4), confirming the RppC oligomerization capacity detected *in vivo* (Figure 2). RppC protomer is composed of six α helices (α1–α6) and two β strands (β1 and β2) that form a long β-hairpin. A DALI search for similar proteins (Holm and Laakso, 2016) revealed that RppC has structural similarities with the MerR transcriptional regulator as well as with other DNA binding proteins, including λ TerS. The structural similarity with these proteins is mainly due to the N-terminal portion (residues 1–64) of RppC that shows a characteristic winged helix-turn-helix (wHTH) DNA-binding fold. This N-terminal DNA binding domain (DBD) includes three α helices (α1–α3) and the β hairpin that correspond to the wHTH wing (Figure S5B). Remarkably, the RppC DBD presents a quite similar fold to that observed for the RMN structure of the N-terminal region of the λ TerS (PDB: 1J9I), showing a root mean square (RMS) deviation of 1.6 Å for the superposition of 51 equivalent C α positions corresponding to this domain (sequence identity 25%) and suggesting that RppC is a DNA binding protein (Figure S5C). Moreover, DALI searches showed structural similarities with the wHTH domain of MerR proteins (root-mean-square deviation [RMSD] of 1.9–3.1 Å for the superimposition of 49–56 C α atoms). MerR family binds to DNA by inserting the recognition helix of the wHTH motif in the major grove and the wing in the minor grove. We modeled the RppC-DNA complex using BldC, a MerR family protein from *Streptomyces*, as a template bound to one of its target promoters (RMSD 2.5 Å for 51 residues superimposed). The model, which was similar to that obtained using other MerR-DNA proteins as templates (data not shown), showed that RppC recognition helix α2 inserts in the DNA major groove with residues R21, T22, R25, K29, and R30 as candidates to direct readout of the DNA, and K39 and K41 projecting from the wing would read out the DNA through the minor groove. Additionally, helix α2 and the wing would also participate in the indirect readout of the operator DNA backbone, suggesting our model of T23, W27, and R43 as candidates to mediate these contacts (Figure S5D). Residues of λ TerS in equivalent positions have been demonstrated to play key roles in the recognition of *cosB* R elements (de Beer et al., 2002, Sippy et al., 2015), suggesting a similar DNA-binding mechanism for RppC and λ TerS as could be expected for the conserved DBD fold (Figure S5D). Notably, the DNA-contacting residues proposed by the model are highly conserved among RppA, RppB, and RppC, pointing out that, if Rpps mediate DNA binding, the operator sequence recognized by these three proteins could be similar. In contrast, Rpps should recognize alternative DNA sequences than λ TerS because the residues located at these positions differ between these two types of proteins. This observation would then explain why EcCICFT073 has a different *cos*B region, although it conserves the *cos*N and *cos*Q regions (Fillol-Salom et al., 2018) for λ TerL function.

**Table 2:** Data Collection and Refinement Statistics

Data Collection	RppC	RppC-TerS^1–98^
Beamline	DLS I04	ESRF ID30B
Wavelength (Å)	0.9795	0.9762
Space group	P4_3_2_1_2	C222_1_
Cell dimensions (Å)	a = 56.88, b = 56.88	a = 60.52, b = 101
	c = 132.27	c = 82.63
	α = β = γ = 90	α = β = γ = 90
Resolution (Å)	43.12–2.42 (2.54–2.4)^a^	50.50–3.00 (3.18–3.00)
Total reflections	46,007 (2,558)	18,007 (3,017)
Unique reflections	7,721 (389)	5,163 (832)
Completeness (%)	86.38 (78)	97.7 (98.7)
Multiplicity	6.0 (6.6)	3.5 (3.6)
Mean I/(σI)	13.6 (1.2)	11.1 (6.7)
Rmerge	0.073 (1.475)	0.064 (0.105)
Rpim	0.033 (0.619)	0.041 (0.066)
CC 1/2	0.999 (0.584)	0.992 (0.992)
Refinement		
Rwork	0.237	0.3124
Rfree	0.267	0.3321
Number of atoms	1,060	1,615
Protein	1,060	1,595
Water	–	20
RMSD, bonds (Å)	0.003	0.016
RMSD, angles (°)	0.679	1.75
Ramachandran Plot		
Preferred (%)	95	86
Allowed (%)	5	14
Outliers (%)	1	0

a Number in parentheses indicates values for the highest-resolution cell.

The remaining C-terminal portion of RppC (residues 65–139) is folded in three α helices (α4–α6) and mediates protein dimerization, especially the long C-terminal α6 helix (residues 110–139) that runs parallel to the α6 helix of the second monomer forming a coiled-coil and providing most of the dimer contacts (Figure 4; Table S2). The dimerization interface buries ∼660 Å^2^ surface area and in helix α6 involves two hydrophobic patches formed by I123, I125, L129, L131, and L134 fringed by polar residues (Q115, R119, Q121, R127 T130, K132, and R133) and, surprisingly for a coiled-coil helix, three Gly residues (G118, G122, and G126) spaced one from each other by one helix turn. Sequence comparison among Rpps showed that residues mediating dimerization are present with low conservation (Figures S1C and S1D), rationalizing the lack of interaction between RppC and RppA observed in our bacterial two-hybrid test assays (Figure 2). Although RppC is a dimer like λ TerS, the available structural data show that both proteins use different surfaces to oligomerize. Although λ TerS does it through the DBD domain, RppC does not use its equivalent domain but rather employs the C-terminal portion.

### Structural Basis of Rpp Function

**Figure 5:**
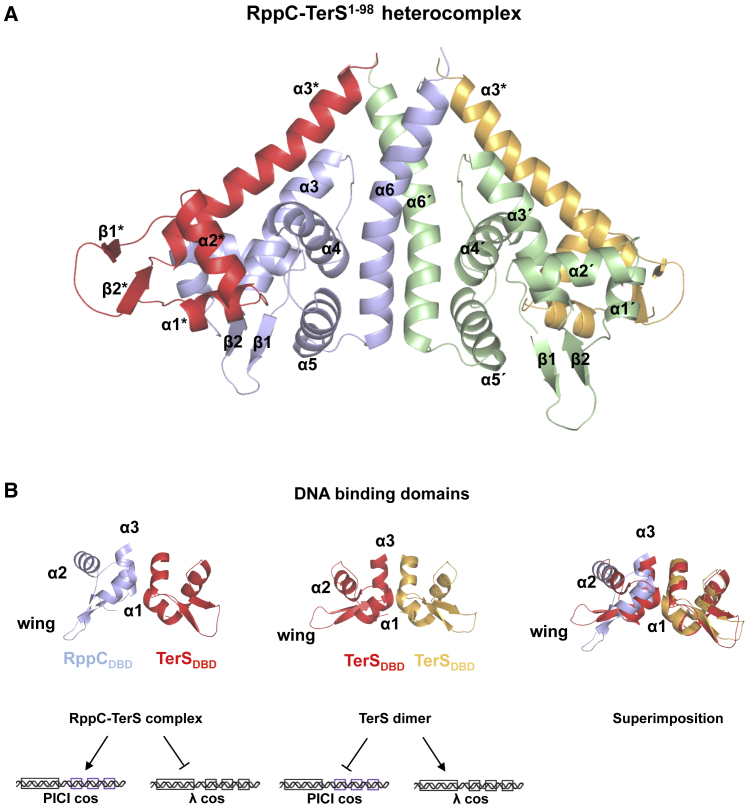
Crystallographic Structure of TerS λ^1–98^ in Complex with RppC (A) Cartoon representation of the RppC-Terλ^1–98^ heterocomplex. RppC monomers are colored in blue and green. TerS λ^1–98^ monomers are colored in red and yellow. Secondary structural elements are numbered and labeled in order from N to C terminus. The apostrophe (') indicates the elements from the second RppC protomer, whereas TerS structural elements are indicated with asterisks (^∗^). (B) The DBD structure from the RppC-TerS heterocomplex (left), involved in PICI *cos* recognition, shows identical folding as the TerS DBD (middle), which specifically recognizes the λ *cos*. Superimposition of the previous DBD structures (right) shows quasi-identical disposition of the dimeric DBDs. Secondary structural elements are labeled in the left protomer. See also Figures S5, S6, and S8 and Table S3.

Next, we attempted to solve the structure of the Rpp in complex with the λ TerS. Unfortunately, λ TerS is highly insoluble, which has hindered its structural characterization. In contrast, the N-terminal DBD portion (residues 1–98) forms soluble dimers that have facilitated its study in solution (de Beer et al., 2002, Yang et al., 1999). We produced the N-terminal soluble portion of λ TerS (TerS^N-ter^ residues 1–98) and confirmed that it maintained its capacity to interact with Rpp (Figure S5A). Importantly, we were able to obtain the crystallographic structure of RppC in complex with the TerS^N-ter^ at 2.8-Å resolution (Figure 5). The asymmetric unit of the crystal showed a heterodimer composed of single copies of RppC and TerS^N-ter^. RppC again exploits the crystallographic symmetry to oligomerize, generating a homodimer almost identical to the observed in the crystal structure of RppC alone (RMSD of 0.6 Å for the superimposition of both homodimers). The RppC dimer interacts laterally with two monomers of TerS^N-ter^ to form a heterocomplex with a TerS^N-ter^-RppC_2_-TerS^N-ter^ tetrameric organization. As in the NMR structure (de Beer et al., 2002), TerS^N-ter^ presents a wHTH fold consisting of three α helices (α1–α3) and two β strands (β1 and β2) with similar overall docking arrangements seen between the X-ray and NMR structures. However, the X-ray and NMR structures differ in the disposition of their C-terminal segments (residues 52–65), which show high mobility in the NMR structure, protruding away from the DBD domain, and in the X-ray structure is stabilized by contacts with RppC and forms part of the long (residues 43–65) α3 helix. Consistent with the mobility of this region observed in NMR experiments, we were unable to trace the 32 C-terminal residues (from 66 to 98) of TerS^N-ter^ that have been proposed as helical linker between the DBD and the oligomerization domain of λ TerS.

However, the most striking observation from the comparison of TerS homodimer and the RppC-TerS heterodimer structures relates to the disposition of the DBDs in the docking. Superimposition of TerS DBD in both structures shows that RppC DBD occupies the same location as that of the second TerS DBD in the homodimer (Figure 5B). This arrangement indicates that RppC hijacks the phage-packaging machinery by mimicking the DNA-binding portion of TerS to form the dimer. λ TerS DBD homodimerizes by the reciprocal interaction of two patches of residues in the α1 (I11, F12, and G13) and α3 (S43, A44, I47, and A51) helices (Table S3). To form the heterocomplex, RppC not only mimics interactions with these TerS residues but also provides additional interactions between its dimerization domain and the three helices of the TerS DBD. In particular, TerS α3 in its new extended conformation runs parallel to RppC α6, forming the C-terminal portion of these helices into a nascent four-helix bundle in the heterotetramer. These additional interactions will favor the formation of the RppC-TerS heterocomplex over the TerS homodimer. Indeed, *in silico* analysis of both complexes with the PRODIGY server (Xue et al., 2016) predicts a higher binding affinity (ΔG −8.8 versus −6.2 kcal/mol) and dissociation constant (3.7 10^−7^ versus 2.8 10^−5^ M) for the heterocomplex than for the homodimer. Sequence comparison of the Rpp family reveals that residues involved in heterocomplex formation are only partially conserved among Rpps, explaining the differences in affinity for these proteins for TerS.

We can now explain the properties of the λ TerS mutants obtained in the *in vivo* evolutionary experiments. The λ TerS E65 is located in the C-terminal part of the α3 helix, and its mutation to Lys would interfere with the formation of the four-helix bundle in the heterotetramer with Rpp. The TerS Y50 is situated in the main interface used by the DBDs to heterodimerize, and its mutation to Asn would have drastic effects in the complex formation (Figure S5E; Table S3).

Importantly, the λ TerS/RppC structure reveals the strategy used by RppC to perform its dual role: first, because the folding of the DBD formed in the heterodimer is the same as that observed for the λ TerS DBD (Figure 5B), this suggests that it will be functional as a DNA binding domain and should be essential for the recognition of the *cos*B site present in the EcCICFT073 island. As previously mentioned, and because our previous results indicated that the phage-encoded TerS was essential for EcCICFT073 packaging (Fillol-Salom et al., 2018), it was a mystery why this element has a different *cos*B site than its helper phage (Figure S1). Our structural data solve this question. Second, following the formation of the heterodimer DBD, the new DBD formed will have reduced affinity for the λ *co*s site, explaining how Rpp blocks phage packaging.

### Functional Characterization of the RppC-TerS Complex

The structural analysis shows that Rpps present two interaction surfaces, one more C-terminal involved in both homo- and heterodimerization and other N-terminal mimicking the TerS DBD used to heterodimerize, and both should be required for Rpp function. In support of this, phages escaping Rpp interference present mutations in λ TerS residues that have a clear impact on the generation of the heterodimer (Figure 2; Table 1). To go further in these studies, and based on the structure of the RppC-λ TerS^N-ter^ complex, two additional mutants were generated and analyzed in RppA. These correspond to L51D and F121R (Figure S5E). Note that the L51 residue is also conserved in RppC. RppA was used instead of RppC because it was not possible to obtain the EcCIEC2733.1 island encoding RppC, so the impact of the different mutations cannot be analyzed *in vivo* (in a well-defined helper phage-PICI system). In contrast, EcCICFT073 encodes RppA, and this element is mobilized by phages λ and 80 (Fillol-Salom et al., 2018). RppA L51 is placed in one of the two DBD patches that nucleate heterodimerization with TerS and F121 is found in α6 helix, the key structural element in Rpp homodimerization. Thus, the L51D mutation may disrupt heterodimerization with TerS, and the F121R mutation would disrupt Rpp homodimerization. These predictions were confirmed using the aforementioned bacterial two-hybrid test. Thus, the RppA L51D mutant formed homodimers with RppA but was incapable of interacting with the λ TerS, and the RppA F121R mutant was incapable of forming homodimers (Figures S6A and S6B). Interestingly, F121R had also impaired capacity to intact with the λ TerS (Figures S6A and S6B), indicating that formation of Rpp dimer is essential for interaction with the λ TerS.

We next utilized complementary strategies to validate these mutations *in vivo*. First, we analyzed the ability of phages λ and 80 to infect an *E. coli* strain expressing from plasmid pBAD18 the two RppA mutants. As expected, none of the mutants blocked phage reproduction (Figure S6C). Second, we introduced the different *rpp*A mutations into the EcCICFT073 *cat* element and tested the ability of the different mutant islands to be mobilized by phages λ and 80. As shown in Figure 3A, the transfer of the island encoding the different RppA mutants was significantly reduced. Taken together, these results confirm that both homo- and heterodimer formation are essential for PICI transfer and phage interference.

**Figure 6:**
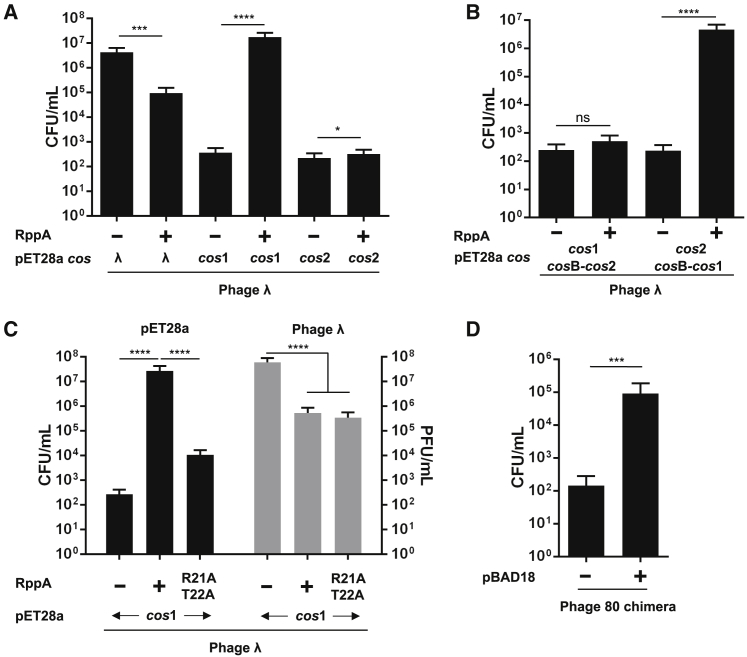
RppA Promotes EcCICFT073 *cos*1 Recognition (A) Strains lysogenic for phage λ containing pET28a with different *cos* sequences (λ, *cos*1, or *cos*2) and pBAD-15A expressing RppA (0.02% arabinose) were MC induced (2 μg/mL) and the transfer of the plasmids analyzed using *E. coli* WG5 as recipient strain. The means of the CFUs and SD are presented (n = 3). An unpaired t test was performed to compare mean differences of each pET28a *cos* plasmid in presence (+) or absence (−) or *rpp*A. Adjusted p values were as follows: λ *cos* (+) versus (−) ^∗∗∗^p = 0.0002; EcCICFT073 *cos*1 (+) versus (−) ^∗∗∗∗^p < 0.0001; and EcCICFT073 *cos*2 (+) versus (−) ^∗^p = 0.04. (B) RppA promotes recognition of the *cos*B region from the EcCICFT073 *cos*1 site. Strains lysogenic for phage λ containing pET28a with different *cos* chimeric sequences were MC induced and the transfer of the different plasmids analyzed. The means of the CFUs and SD are presented (n = 3). An unpaired t test was performed to compare mean differences of each pET28a *cos* plasmid in presence (+) or absence (−) or *rpp*A. Adjusted p values were as follows: ^∗∗∗∗^p < 0.0001. (C) Strains lysogenic for phage λ containing pET28a with the *cos*1 site and pBAD18-15A expressing RppA WT or RppA R21A T22A were MC induced, and the transfer of the plasmid with the pET28a *cos*1 (left, black bars) or the titer of the λ phage (right, gray bars) was analyzed. The means of results and SD are presented (n = 3). A one-way ANOVA with Dunnett’s multiple comparisons test was performed to compare mean differences between samples. Adjusted p values were as follows: ^∗∗∗∗^p < 0.0001. (D) The strain lysogenic for chimeric phage 80 with the *cos*B region from the EcCICFT073 *cos1* site was MC induced, in presence or absence of RppA, and the transfer of the chimeric phage 80 to recipient *E. coli* 594 was analyzed. The means of CFUs and SD are presented (n = 3). An unpaired t test was performed to compare phage 80 chimera (−) against (+). Adjusted p values were as follows: ^∗∗∗^p = 0.0011. See also Figures S5 and S7.

Our structural data led us to hypothesize that the interaction of Rpp with the phage TerS generates a new DBD that specifically recognizes the EcCICFT073 DNA, but not the λ *cos*B, site. Because λ TerS has shown low-affinity and non-specific DNA binding activity *in vitro* that have forced the use of genetic experiments to dissect its DNA packaging specificity (Frackman et al., 1985, Sippy et al., 2015), we decided to perform additional experiments *in vivo* to test our hypothesis. In the first one, we introduced independently the λ, 80, or each of the two EcCICFT073 *cos* sites (containing the putative *cos*Q, *cos*N, and *cos*B sequences) into plasmid pET28a, which is not transferrable by phages λ or 80, and found that the cloned *cos* sites enabled transfer of the plasmids by these phages (Figures 6A and S7). Consistent with the presence of completely different *co*sB sequences, transfer of the plasmids carrying the EcCICFT073 *cos* sites was reduced compared to that observed with the plasmids carrying the cognate phage *cos* sequences (Figures 6A and S7). We next performed the same experiments but in presence of RppA expressed from plasmid pJP2233, a pBAD derivative plasmid carrying a different origin of replication to avoid plasmid incompatibilities. In support of the proposed model, expression of RppA significantly reduced phage-mediated transfer of the plasmid carrying the phage *cos* sites but significantly increased the transfer of the plasmid carrying the EcCICFT073 *cos*1 site (Figures 6A and S7). This result also explains why the *cos*1 site, but not the *cos*2, is essential for EcCICFT073 transfer. To further demonstrate that the RppA-TerS complex recognizes the EcCICFT073 *cos*B region present in the *cos*1 site, we swapped the *cos*B regions present in the EcCICFT073 *cos*1 and *cos*2 sites and analyzed the ability of these chimeric *cos* sites to be transferred by phage λ in presence of RppA. As shown in Figure 6B, only those plasmids carrying the *cos*B region from the *co*s1 site were transferred in presence of RppA.

Our model proposes that RppA interacts specifically with the EcCICFT073 *cos*1 site via the residues in the α2 helix (Figure S5D). To test this hypothesis, we generated a RppA mutant in which the residues R21-T22 were mutated to alanine. Note that these residues are essential components of the α2 helix (Figure 4) and are conserved in both RppB and RppC proteins (Figure S1). Next, the impact of this mutation in both phage interference and in the transfer of the plasmid carrying the EcCICFT073 *cos*1 site was analyzed. In support of our model, the RppA mutant retained its capacity to block phage packaging but was unable to promote the preferential packaging of the island (Figure 6C). Finally, we generated a chimeric 80 prophage in which the *cos*B region from the EcCICFT073 *cos*1 site replaced the phage *cos*B site. This chimeric prophage also contained a *cat* marker, which is used to test lysogenic conversion in *E. coli*. Next, we introduced into the strain lysogenic for the chimeric 80 prophage either the empty plasmid pBAD18 or the pBAD18 derivative expressing RppA. The different strains were induced with MC, the cultures lysed, and the number of lysogens generated in the *E. coli* 594 strain analyzed. In the absence of the Rpp protein, the chimeric 80 phage carrying the EcCICFT073 *cos*B site generated a small number of lysogens (Figure 6D). In contrast, expression of RppA significantly increased packaging and transfer of the chimeric phage, supporting the model that the RppA-TerS complex specifically recognizes the EcCICFT073 *cos*B, but not the phage *cos*B, site.

### The Pirating Mechanism Involving Rpp Proteins Is Widespread in Nature

To generalize our results, we analyzed whether the Rpp homologs found in PICIs from different species (Table S1) also work by the same mechanisms as the *E. coli* Rpp proteins. To test this, we selected the Rpp protein from *Pluralibacter gergoviae* (GenPept: WP_086499225) and scrutinized the *P. gergoviae* genomes to find phage-encoded TerS proteins, which would be the target of the Rpp protein. One of these proteins was selected (GenBank: KMK30155.1) and its interaction with the *P. gergoviae* Rpp analyzed, using the two-hybrid system assay. As shown in Figures S8A and S8B, the *P. gergoviae* Rpp forms dimers and interacts with the *P. gergoviae* phage-encoded TerS. Interestingly, the *E. coli* RppC (but not the RppA) is also able to interact with the *P. gergoviae* phage-encoded TerS, and the *P. gergoviae* Rpp is able to interact with the λ TerS, but not with the evolved λ phages carrying mutations in the *ter*S gene (Figures S8A and S8B). Together, these results strongly suggest that all the Rpp proteins are structurally related. In fact, overexpression of the *P. gergoviae* Rpp protein interferes with λ phage reproduction (Figure S8C), confirming the idea that the Rpp proteins have a conserved and widespread mechanism of action. In summary, our results decipher the fascinating mechanism of action that allows the Gram-negative *cos* PICI elements to be packaged and disseminated in nature.

## Discussion

Two key features in the PICI lifestyle have been well conserved among all the PICIs analyzed so far: their capacity to interfere with phage reproduction and their ability to hijack the phage machinery for their own packaging and transfer. The most conserved strategy of PICI-mediated interference with phage reproduction is the production, using the phage-encoded proteins, of PICI small capsids, which are commensurate to the size of the PICI genomes (Penadés and Christie, 2015). Because PICI genomes are usually 1/3 in size than their helper phages, this strategy impairs packaging of a full-phage genome in the PICI capsids.

However, the production of PICI-sized capsids, although interfering with phage reproduction, does not favor packaging of the PICI element, suggesting PICIs require complementary strategies to increase their transferability in nature. Until now, only one of these strategies had been discovered, used by the prototypical members of the PICI family, the *Staphylococcus aureus* pathogenicity islands (SaPIs). SaPIs that use the headful mechanism for packaging (*pac* SaPIs) encode a homolog of the phage terminase small subunit (TerS_S_) that specifically recognizes the SaPI genome and directs the packaging of the SaPIs into the SaPI- or phage-sized capsids (Ubeda et al., 2007). To help with this preferential packaging, *pac* SaPIs encode Ppi (for phage packaging interference; Ram et al., 2012), which binds to the phage terminase small subunit (TerS_P_), but not to the SaPI TerS_S_, blocking phage TerS_P_ function. This process would favor SaPI packaging by facilitating the TerS_S_-TerL interaction, and at the same time, this would block phage packaging by blocking the formation of the TerS_P_-TerL complex (Ram et al., 2012). However, the exact mechanism by which Ppi performs its function remains to be deciphered.

In contrast to the two-shot strategy (SaPI TerS + Ppi) used by the *pac* SaPIs to promote their transfer, blocking helper phage reproduction, the Gram-negative *cos* PICIs have evolved an elegant one-shot strategy in which the same protein, Rpp, is used to perform both processes. This mechanism explains why the *cos E. coli* PICI has a *cos*B site different to that present in their inducing phages and why this strategy is so efficient in simultaneously performing both processes. Our structural data reveal that Rpps present a DBD domain structurally similar to TerS, suggesting that this protein could substitute for TerS in the *cosB* R elements binding. Because the structural models propose that TerS and Rpps present alternative residues in the key positions for DNA recognition, the R elements of the phage and the PICIs should differ, explaining why λ TerS R elements are not present in *cosB* region of EcCICFT073. Remarkably, the residues involved in DNA recognition seem to show some conservation among Rpps, suggesting that the R elements present are similar. This fact could open the door to a certain degree of promiscuity, and the genomes of nearby related islands could be packaged in different capsids, thus ensuring a high degree of transference.

Does the interaction of Rpp with TerS only aim to deprive the phage of this essential protein required for its own packaging? We do not think this is correct. Although this strategy blocks phage packaging, the Rpp-TerS interaction also allows the recruitment of the phage–packaging machinery. TerS interacts with TerL by its C-terminal portion (residues 100–181; Frackman et al., 1985, Wu et al., 1988). This region is dispensable for the interaction with RppC, as confirmed by the RppC-TerS structure, where it is absent. Indeed, we were unable to trace the 30 C-terminal residues of TerS in the RppC-TerS structure (residues 67–96), which precedes the TerL-interacting region, confirming the independent functions of the TerS domains involved in the RppC or TerL binding. In this scenario, it is tempting to speculate that the TerS C-terminal domains that project from the body of TerS-RppC heterocomplex are free to recruit TerL. Once TerL is recruited, the core of the catalytically component terminase complex is formed (Maluf et al., 2006) and other components of the phage machinery can then be hijacked, completing the elegant one-shot strategy developed by the PICIs to promote their preferential packaging.

Fascinating questions about the evolutionary history of the Gram-negative PICIs are raised by this study. What is the origin of the Rpp proteins? Why do different Rpps exist? Why do some PICIs have two different *cos* sites? The fact that TerS and Rpp share conserved DBD domains, including some sequence identity, suggests that these proteins either have a common ancestor or more likely the Rpp proteins have evolved from TerS. This evolution has generated Rpp proteins that perform some functions (DNA recognition) similarly to TerS. But they only work by forming a complex with TerS, explaining why the Rpp proteins affect phage packaging. This parasitic evolution has also generated Rpp variants, all with the ability to interfere with TerS function but unable to interact and interfere with the activity of the other Rpp proteins. With this strategy, and in the case of a strain containing several PICIs encoding different Rpp proteins, all the PICIs would be able to hijack the phage machinery for packaging without generating Rpp heterodimers that could affect the transfer of the different PICIs. Furthermore, sequence similarities would indicate some packaging promiscuity among PICIs. It is clear for all these scenarios that the PICIs are independently evolving genetic elements that have fine-tuned multiple strategies to spread in nature.

## STAR★Methods

### Key Resources Table

**Table:** 

REAGENT or RESOURCE	SOURCE	IDENTIFIER
**Antibodies**		

Anti-Digoxigenin-AP, Fab fragments	Sigma-Aldrich (Roche)	11093274910

**Bacterial and Virus Strains**		

Bacterial strains, see Table S4	N/A	N/A

**Chemicals, Peptides, and Recombinant Proteins**		

LB medium	Sigma-Aldrich	L3022
Bacteriological agar	Sigma-Aldrich	A5306; CAS 9002-18-0
Nutrient Broth No. 2	ThermoFisher (Thermo Scientific)	Cat#CM0001
Platinum® Taq DNA Polymerase High Fidelity	ThermoFisher (Invitrogen)	Cat#11304011
DreamTaq DNA Polymerase	ThermoFisher (Thermo Scientific)	Cat#EP0703
2,3,5-Triphenyltetrazolium chloride	Sigma-Aldrich	T8877; CAS 298-96-4
L-(+)-Arabinose	Sigma-Aldrich	A3256; CAS 5328-37-0
IPTG	Sigma-Aldrich	I6758; CAS 367-93-1
Thermo Scientific X-Gal	FisherScientific	10490470
4-Nitrophenyl β-D-galactopyranoside	Sigma-Aldrich	N1252; CAS 3150-24-1
Digoxigenin-11-dUTP, alkali-stable	Sigma-Aldrich (Roche)	11093088910
Lysozyme from hen egg white	Sigma-Aldrich (Roche)	10837059001
Proteinase K from Tritirachium album	Sigma-Aldrich	P2308
Nylon Membranes, positively charged	Sigma-Aldrich (Roche)	11417240001
Anhydrotetracycline hydrochloride	Sigma-Aldrich	37919; CAS 13803-65-1
Ampicillin sodium salt	Sigma-Aldrich	A9518; CAS 69-52-3
Kanamycin Sulfate	Sigma-Aldrich	60615; CAS 70560-51-9
Chloramphenicol	Sigma-Aldrich	C0378; CAS 56-75-7
Tetracycline	Sigma-Aldrich	T3258; CAS 60-54-8
HisPur Ni-NTA Resin	ThermoFisher (Thermo Scientific)	Cat#88221
SelenoMethionine Solution	Molecular Dimensions	Cat#MD12-503B
Mitomycin C	Sigma-Aldrich	M0503; CAS 50-07-7
Crystallization screenings JBS I, JBS II	Jena Biosciences	Cat#CS114-L
Crystallization screening JCSG	Molecular Dimensions	Cat#MD1-40

**Critical Commercial Assays**		

QIAquick PCR Purification Kit	QIAgen	Cat#28106
QIAprep Spin Miniprep Kit	QIAgen	Cat#27106

**Deposited Data**		

Atomic coordinates of RppC	This paper	6HLK
Atomic coordinates of the RppC-Terλ^1-98^ heterocomplex	This paper	6HN7
Original data in Mendeley dataset	This paper	https://doi.org/10.17632/m64fw49kr8.2

**Oligonucleotides**		

Primers used in this study, see Table S5	N/A	N/A

**Recombinant DNA**		

Plasmids used in this study, see Table S6	N/A	N/A

**Software and Algorithms**		

GraphPad prism	GraphPad Software	https://www.graphpad.com/scientific-software/prism/
Mosflm	Powell et al., 2013	https://www.mrc-lmb.cam.ac.uk/harry/imosflm/ver722/introduction.html
Aimless	Evans and Murshudov, 2013	http://www.ccp4.ac.uk/dist/html/aimless.html
Phenix suite	Adams et al., 2010	http://www.phenix-online.org/
CCP4 suite	Winn et al., 2011	http://www.ccp4.ac.uk/
Phaser	McCoy et al., 2007	http://www.ccp4.ac.uk/html/phaser.html
Refmac	Murshudov et al., 2011	http://www.ccp4.ac.uk/html/refmac5.html
Coot	Emsley et al., 2010	https://www2.mrc-lmb.cam.ac.uk/personal/pemsley/coot/

### Lead Contact and Materials Availability

Further information and requests for reagents should be directed to Lead Contact José R Penadés (joser.penades@glasgow.ac.uk).

### Method Details

#### Bacterial Strains and Growth Conditions

Bacterial strains used in this study are listed in Table S4. Strains were grown at 37°C or 30°C on Luria-Bertani (LB) agar or in LB broth with shaking (180 rpm). Ampicillin (100 μg ml^-1^), Kanamycin (30 μg ml^-1^), Chloramphenicol (20 μg ml^-1^) or Tetracycline (20 μg ml^-1^; all Sigma-Aldrich), were added when appropriate.

#### Induction

Bacteria were grown in LB broth to OD_600_ = 0.2 and induced by adding mitomycin C (2 μg ml^-1^). Cultures were grown at 32°C with gentle shaking (80 rpm). Generally, cell lysis occurred 4-5 h post-induction. The number of phage particles in a lysate was quantified using the titering assay. A 1:50 dilution (in fresh LB broth) of an overnight culture of the appropriate *E. coli* recipient strain was prepared and grown until OD600 = 0.3-0.4 was reached. Strains were infected using 50 μL of the recipient culture with the addition of 100 μL of phage lysate serial dilutions, prepared with phage buffer, and incubated for 5 min at room temperature. The different mixtures of culture-phage dilution were plated out on phage base agar plates (PBA; 25 g of Nutrient Broth No. 2, Oxoid; 7g agar) supplemented with CaCl_2_ to a final concentration of 10mM. PBA plates were kept at room temperature to set up and, afterward, were incubated at 37°C for 24 h. The number of plaques formed (phage particles present in the lysate) were counted and the plaque forming units (PFU) estimated. PBA plates were stained to enhance plaque visibility in the images taken. At least, 6 mL of LB supplemented with 0.1% (w/v) 2,3,5-triphenyltetrazolium chloride (TTC) solution was added per PBA plate and incubated for 30 min at room temperature. Plaques remained unstained due to only living bacteria being able to reduce TTC dye to red formazan.

The PICIs or phage 80 derivatives used in this work contained a *tet*A or *cat* antibiotic cassette. These markers allow for selection of the PICI or phage on selective LB plates, supplemented either with 20 μg/ml tetracycline or 20 μg/ml chloramphenicol. In plasmid transduction experiments, plasmids were selected based on their plasmid antibiotic resistance gene. Transduction titering assays were performed in *E. coli* using strain 594 as recipient. A 1:50 dilution of an overnight culture (in fresh LB broth) was prepared and grown until OD_600_ = 1.4 was reached. Strains were infected using 1 mL of the recipient culture with the addition of 100 μL of phage lysate serial dilutions, prepared in phage buffer, and cultures were supplemented with CaCl_2_ to a final concentration of 4.4 mM before incubation for 30 min at 37°C. This incubation allows the PICI or phage to infect the acceptor strain. The different culture-phage dilutions were plated out on LBA plates containing the PICI-phage-plasmid appropriate antibiotic. LBA plates were kept at room temperature to set up and, afterward, were incubated at 37°C for 24 h. The number of colonies formed (PICI-phage-plasmid particles in a lysate) were counted and the colony forming units (CFU) were estimated.

#### DNA Methods

Gene insertions or deletions were performed as described (Datsenko and Wanner, 2000). The chloramphenicol (*cat*) or kanamycin resistance (*km*R) makers were amplified by PCR, with primers listed in Table S5, and inserted in the PICI genome using λ Red recombinase-mediated recombination. The PCR product was transformed into the recipient strain harboring plasmid pKD46, which expresses the λ Red recombinase. The insertion of the resistance markers were verified by PCR. Site-directed scarless mutagenesis was performed as described previously (Hoffmann et al., 2017, Blank et al., 2011). The *km*R marker together with an I-*Sce*I recognition restriction site was amplified by PCR, using primers listed in Table S5, and inserted into the recipient strain harboring plasmid pRWG99, which expresses the λ Red recombinase protein. After verification of the insertion by PCR, 80-mer DNA fragments derived from oligonucleotides or PCR products were electroporated into the mutant strain expressing the λ Red recombinase-mediated system. Successful recombinants were selected by expression of I-*Sce*I endonuclease. The different mutants obtained were subsequently verified by PCR and DNA sequencing.

#### Plasmid Construction

The plasmids used in this study (Table S6) were constructed by cloning PCR products, amplified with the oligonucleotides listed in Table S5 (Sigma-Aldrich), into the appropriate vectors. The cloned plasmids were verified by Sanger sequencing (Eurofins Genomics). Synthetic plasmids were purchased from DC BIOSCIENCES Limited.

#### Phage Evolution

Phages were evolved to overcome either the plasmid- or the PICI-mediated interference. The phage plaques obtained after infection of the appropriate strains were collected in a tube containing phage buffer (50 mM Tris pH = 8, 1 mM MgSO_4_, 4 mM CaCl_2_ and 100 mM NaCl). Tubes were centrifuged at 5000 rpm for 10 min. The supernatant was filtered using a sterile 0.2 μm filter (Minisart® single use syringe filter unit) and the resultant lysate was used in a new round of phage infection. Consecutive rounds of phage infection, collection of the top layer and generation of new lysate, were performed until the phage overcame the mediated-PICI or plasmid interference. Then, single plaques of insensitive phage mutants were selected to generate individual phage lysogenic strains, which were sequenced by whole genome sequencing.

#### Southern Blot

Following plasmid (0.02% arabinose; Sigma-Aldrich) and phage (mitomycin C; Sigma-Aldrich from *Streptomyces caespitosus*) induction, samples were taken at defined time points and pelleted. Samples were re-suspended in 50 μL lysis buffer (47.5 μL TES-Sucrose and 2.5 μL lysozyme [10 μg ml^-1^]; Sigma-Aldrich) and incubated at 37°C for 1 h. Then, 55 μL of SDS 2% proteinase K buffer (47.25 μL H_2_O, 5.25 μL SDS 20%, 2.5 μL proteinase K [20 mg ml^-1^], Sigma-Aldrich from *Tritirachium album*) was added to the obtained lysates and incubated at 55°C for 30 min. Lysates were vortexed with 10 μL of 10x loading dye for 1h. Samples were frozen and thawed in cycles of 5 min incubation in dry ice with ethanol and in a water bath at 65°C. This cycle was repeated three times. Chromosomal DNA was separated by agarose gel electrophoresis by running samples on 0.7% agarose gel at 30V, overnight. The DNA was transferred to Nylon membranes (Hybond-N 0.45 mm pore size filters; Amersham Life Science) using standard methods. DNA was detected using a DIG-labeled probe (Digoxigenin-11-dUTP alkali-labile; Roche) and anti-DIG antibody (Anti-Digoxigenin-AP Fab fragments; Roche), before washing and visualization. The primers used to obtain the DIG-labeled probes are listed in Table S5.

#### Two-Hybrid Assay

The two-hybrid assay for protein-protein interaction was conducted as previously described (Battesti and Bouveret, 2012) using two compatible plasmids; pUT18C and pKT25, expressing the different protein combinations. Both plasmids were co-transformed into *E. coli* BTH101 for the Bacterial Adenylate Cyclase Two Hybrid (BACTH) system and plated on LB supplemented with ampicillin, kanamycin, 0.1 mM of isopropyl-b-D thiogalactopyranoside (IPTG) and X-gal as an indicator. After incubation at 30°C for 24-48 h, the protein-protein interaction was detected by a color change. Blue colonies represent an interaction between the two clones, while white/yellow colonies are negative for any interaction.

For quantification of the BACTH analysis, strains were grown overnight at 37°C in LB medium containing the appropriate antibiotics and 0.5 mM IPTG. Following overnight induction, a 1 mL aliquot of each strain was pelleted. The Miller method was used to measure β-galactosidase activity levels, using ortho-Nitrophenyl-β-galactoside (ONPG; Sigma-Aldrich) as the substrate. Pellets were re-suspended in the same volume of chilled Z buffer (0.06 M Na_2_HPO_4_x7H_2_O, 0.04 M NaH_2_PO_4_xH_2_O, 0.01 M KCl, 0.001 M MgSO_4_ and 0.05M β -mercaptoethanol). The OD_600_ of the re-suspended pellets was measured. The re-suspended cells were diluted in Z buffer to 1 mL (0.1 mL cells + 0.9 mL Z buffer) and cells were permeabilized by adding 100 μL chloroform and 50 μL 0.1% SDS. Immediately after, the mix was vortexed and the tubes were equilibrated for 5 min in a 28°C water bath. The reaction was initiated by adding 0.2 mL of ONPG (4 mg/mL). The time of addition was recorded precisely with a timer. Immediately, the mix was vortexed and the tubes were incubated at 28°C in a water bath. When sufficient yellow color was observed, the reaction was stopped by adding 0.5 mL 1 M Na_2_CO_3_. The time of addition was recorded precisely and the mix was vortexed. Following this, 1 mL of sample was transferred to Eppendorf tubes and centrifuged for 5 minutes at maximum r.p.m and the OD at 420nm and at 550nm for each tube was recorded. The average of at least three independent experiments is shown in Miller units.

#### Protein Expression and Purification

Proteins were overexpressed from *Escherichia coli* BL21 (DE3) (Novagen) cells transformed with the corresponding expression plasmids (Table S6). Cultures were grown at 37°C in LB medium supplemented with 100 μg ml^-1^ ampicillin to an OD_600_ of 0.5–0.6. Then, protein expression was induced with 1 mM IPTG at 16°C for 16 h. Cells were harvested by centrifugation at 4°C, 4000 rpm for 30 min, resuspended in lysis buffer (100 mM Tris pH = 8, 300 mM NaCl, 1 mM TCEP) and lysed by sonication. The soluble fractions were obtained by centrifugation at 4°C, 15000 rpm for 1h and loaded onto a pre-equilibrated Nickel affinity gravity column (HisPur^TM^ Ni-NTA Resin; Thermo Fisher). After two washes with 20 mM (40x bed volume) and 50 mM imidazole (30x bed volume), the proteins were eluted with lysis buffer containing 250 mM imidazole. The fractions were analyzed by SDS-PAGE and those fractions showing purest protein were selected, concentrated, and stored at −80°C.

For anomalous X-ray diffraction and phasing, RppC was selenomethionine-labeled (SeMet) by expressing the protein in SelenoMethionine Medium Complete (Molecular Dimensions Ltd; MD 12-500), according to the manufacturer instructions, and purified as described previously.

#### Protein Crystallization and Data Collection

Crystals of the RppC protein or the RppC-λTerS^N-ter^ heterocomplex were obtained by vapor-diffusion technique using a sitting drop setup at 15°C. Crystallization drops were generated by mixing equal volumes of each protein solution and the corresponding reservoir solution, and were equilibrated against 100-300 μL reservoir solution. SeMet derivative RppC was crystallized at 15 mg ml^-1^ in a reservoir solution of 15% PEG8K, 0.1 M sodium acetate pH = 6. The heterocomplex was crystallized at 10 mg ml^-1^ in a reservoir solution of 2 M ammonium acetate, 0.1 M sodium acetate (pH = 5). The crystals were cryo-protected using 25%–35% of glycerol solution when freezing in liquid nitrogen. X-ray data collection was carried out at 110K. RppC was collected by Single-wavelength anomalous diffraction (SAD) on the I04 beamline at the Diamond Light Source synchrotron radiation facility (DLS; Didcot, UK) at a wavelength of 0.9795Å. X-ray data of RppC-λTerS^N-ter^ heterocomplex was collected on the beamline ID-30B of the European Synchrotron Radiation Facility (ESRF; Grenoble, France). Data from SeMet-labeled RppC were indexed, integrated, and scaled using the program autoPROC (Vonrhein et al., 2011), whereas the heterocomplex data were processed and reduced with Mosflm (Powell et al., 2013) and Aimless (Evans and Murshudov, 2013) programs. The crystallographic parameters and data-collection statistics are listed in Table 2.

#### Model Building and Refinement

Solution and refinement of the crystallographic structure of RppC was performed with the *Phenix* suite (Adams et al., 2010). Automated structure solution using SAD phasing technique was carried out on the Autosol pipeline of Phenix, and a total of 4 selenium atoms were localized, which was enough to calculate experimental phasing and model building.

Structure of RppC-λTerS^N-ter^ was solved using the CCP4 suite (Winn et al., 2011). Phases were obtained by molecular-replacement using Phaser (McCoy et al., 2007). The structure of the RppC monomer (obtained previously) was used as a model, as well as the monomer of the λ TerS DNA binding domain (PDB: 1J9I; (de Beer et al., 2002)). All the final models were generated by iterative cycles of refinement using Refmac (Murshudov et al., 2011) and manual optimization with Coot (Emsley et al., 2010). Data refinement statistics are given in Table 2. Atomic coordinates and structure factors have been deposited in the PDB (Key Resources Table).

### Quantification and Statistical Analysis

All statistical analyses were performed as indicated in the figure legends using GraphPad Prism 6.01 software, where n represents the number of independent experiments.

### Data and Code Availability

Coordinates for atomic structures have been deposited at the RCSB Protein Data Bank (PDB: 6HLK and PDB: 6HN7). The original data and figures have been deposited in Mendeley dataset (https://doi.org/10.17632/m64fw49kr8.2).

## Supplemental Information

Document S1. Figures S1–S8 and Tables S1–S4 and S6

Table S5. Oligonucleotides Used in This Study, Related to STAR Methods

Document S2. Article plus Supplemental Information
