# Supplementary material for: Hijacking the Hijackers: Escherichia coli Pathogenicity Islands Redirect Helper Phage Packaging for Their Own Benefit
Source: Mol Cell. 2019 Sep 5;75(5):1020–1030.e4. doi: 10.1016/j.molcel.2019.06.017 (PMC6739421; doi:10.1016/j.molcel.2019.06.017)
Supplement: Document S1. Figures S1–S8 and Tables S1–S4 and S6 [file mmc1.pdf]

Molecular Cell, Volume *75*

Supplemental Information

Hijacking the Hijackers: *Escherichia coli* Pathogenicity Islands Redirect Helper Phage Packaging for Their Own Benefit

Alfred Fillol-Salom, Julio Bacarizo, Mohammed Alqasmi, J. Rafael Ciges-Tomas, Roser Martínez-Rubio, Aleksander W. Roszak, Richard J. Cogdell, John Chen, Alberto Marina, and José R. Penadés

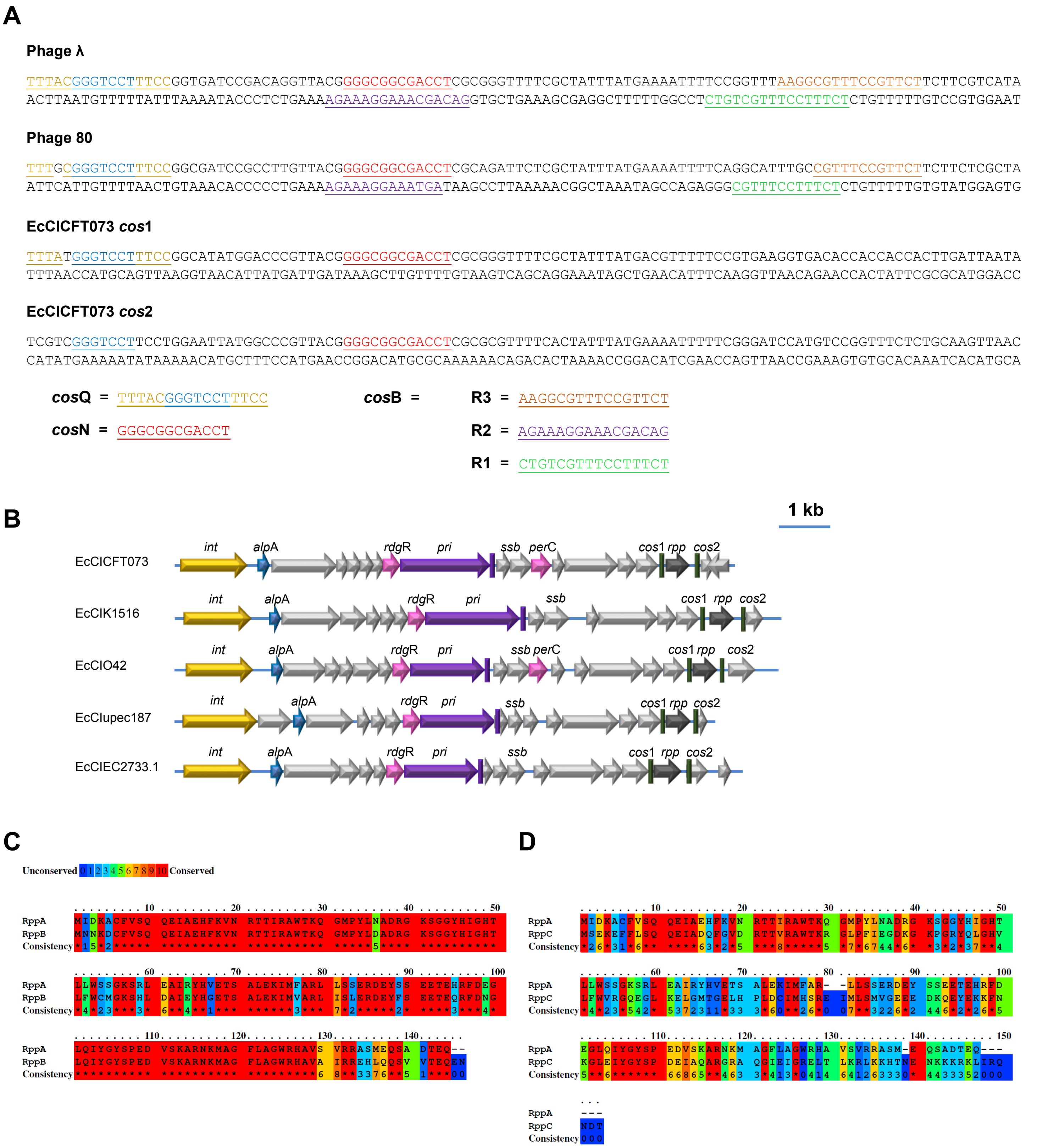

Figure S1. *cos* and Rpp sequences. Related to Figures 1 and S2 and Table S1.
(A) Sequence of the *cos* regions from phages λ and 80 and from the EcCICFT073 element. While all the elements carry the same *cos*Q and *cos*N sequences, the EcCICFT073 PICI *cos*B sequences are completely divergent.
(B) Genome maps for *E. coli* PICIs encoding *rpp* genes. Genomes are aligned according to the prophage convention, with the integrase gene (*int*) at the left end. Genes are coloured according to their function: *int* is yellow; transcription regulator (*alp*A) is dark blue; replication genes (*pri*) are purple; redirecting phage packaging genes (*rpp*) are grey; virulence genes are pink; other accessory genes are red; genes encoding hypothetical proteins are white. *cos* sites are indicated as green rectangles.
(C-D) Rpp homologues in *E. coli* PICIs. Protein sequence alignment of RppA and RppB (C) or RppA and RppC (D), generated using the PRALINE server. Colours indicate relative sequence conservation at each position, with red being most conserved and blue least.

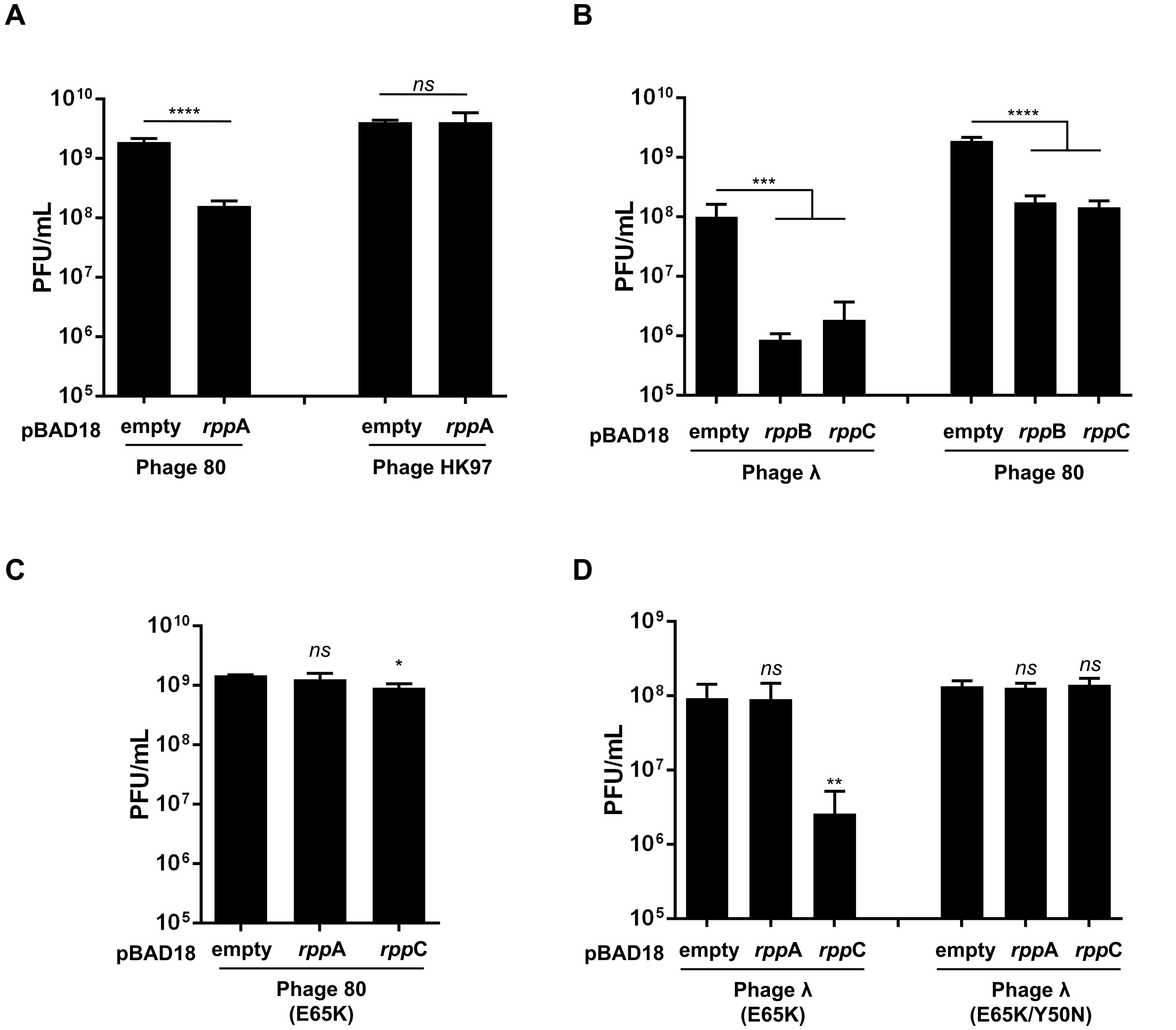

Figure S2. The Rpp homologues block helper phage reproduction. Related to Figures 1, 2 and S1.
(A) *E. coli* strain 594 expressing RppA from plasmid pBAD18 was infected with phages 80 or HK97 and plated on phage base agar plates supplemented with 0.1% arabinose using phage top agar. Plates were incubated for 24 h at 37°C and the number of phage plaques were quantified. The means and standard deviation from three independent experiments are presented (n=3). An unpaired t-test was performed to compare mean differences within rows. Adjusted *p* values were as follows: $p<0.0001^{****}$. *ns*, not significant.
(B) *E. coli* strain 594 containing different pBAD18 derivatives was infected with phage lambda or phage 80 and plated on phage base agar plates supplemented with 0.1% arabinose using phage top agar. Plates were incubated for 24 h at 37°C and the number of phage plaques quantified. The means of results and standard deviation from three independent experiments are presented (n=3). A 1-way ANOVA with Dunnett's multiple comparisons test was performed to compare mean differences between pBAD18 empty row. Adjusted *p* values were as follows: λ: empty vs *rpp*B $p=0.0003^{***}$, empty vs *rpp*C $p=0.0005^{***}$, $p<0.0001^{****}$.
(C-D) Phage 80 and λ mutants insensitive to the RppA and RppC interference. *E. coli* strain 594 containing different pBAD18 derivatives was infected with the evolved phage 80 carrying the TerS E65K mutation (C) or with evolved λ mutants (TerS E65K or E65K/Y50N; D) and plated on phage base agar plates supplemented with 0.1% arabinose using phage top agar. Plates were incubated for 24 h at 37°C. The means of results and standard deviation from three independent experiments are presented (n=3). A 1-way ANOVA with Dunnett's multiple comparisons test was performed to compare mean differences between samples. Adjusted *p* values were as follows: $p=0.0337^{*}$, $p=0.0010^{**}$. *ns*, not significant.

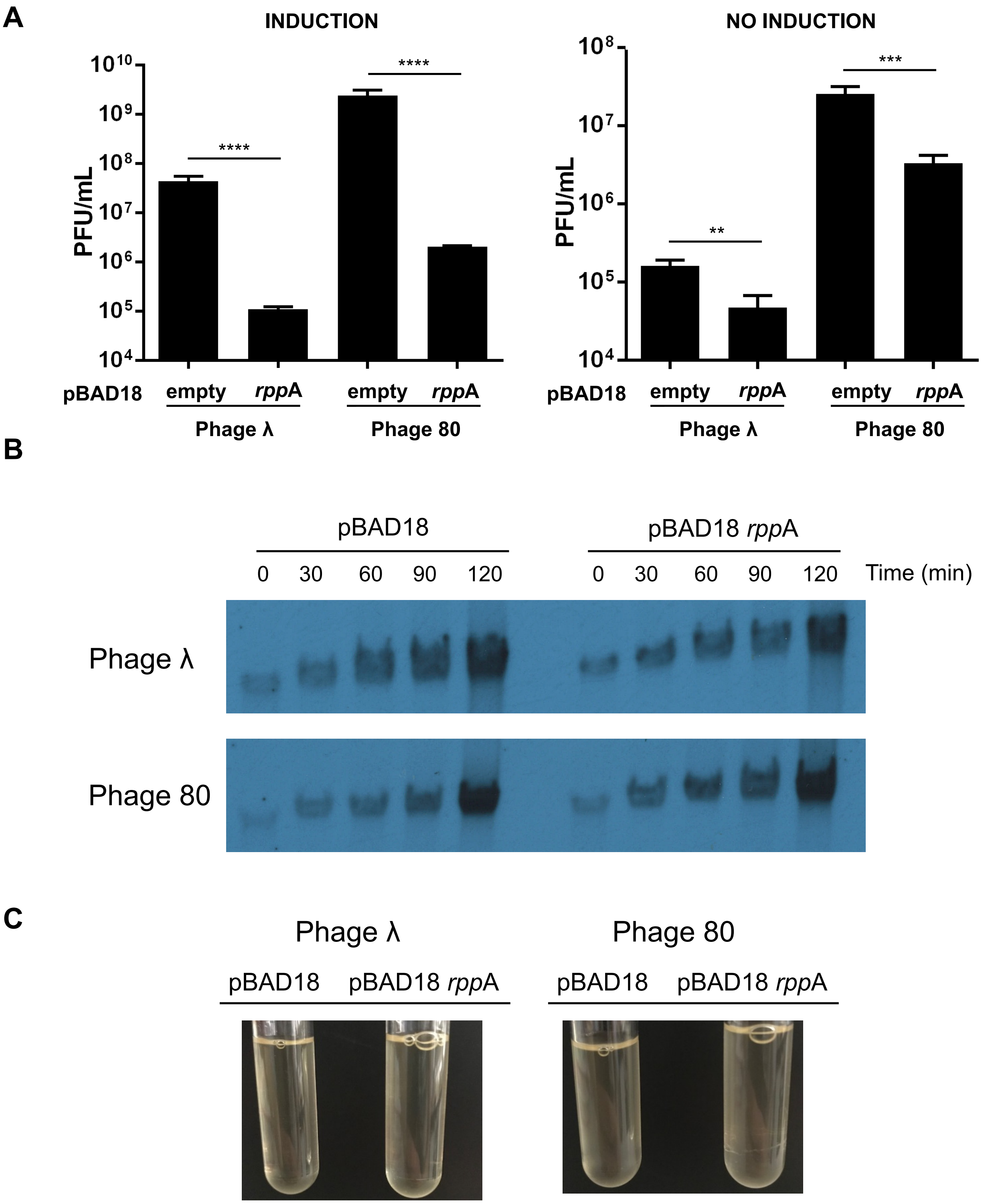

Figure S3. Identification of the Rpp target. Related to Figure 2.

(A) Lysogenic strains for phage λ or 80, carrying plasmid pBAD18 empty or expressing RppA, were induced (left) or not (right) with MC, and the titre of the phage analysed. Expression of RppA from plasmid pBAD18 was induced using 0.02% arabinose when appropriate. The experiment shows the no. of plaques/mL of lysate, using *E. coli* 594 as recipient strain. The means of results and standard deviation from three independent experiments are presented (n=3). An unpaired t-test was performed to compare mean differences within rows. Adjusted *p* values were as follows: $p<0.0001^{****}$, $p=0.0006^{***}$, $p=0.0079^{**}$.

(B) Phage replication after expression of the cloned *rpp*A gene. Lysogenic strains for phage λ or 80 carrying plasmid pBAD18 empty or expressing RppA, were induced and one millilitre of each culture at different time points after induction was collected and used to prepare standard minilysates, which were resolved on a 0.7% agarose gel, Southern blotted and probed for phage DNA.

(C) RppA does not affect phage lysis. Lysogenic strains for for phage λ or 80, carrying plasmid pBAD18 empty or expressing RppA, were MC induced and photographed 6 h after induction.

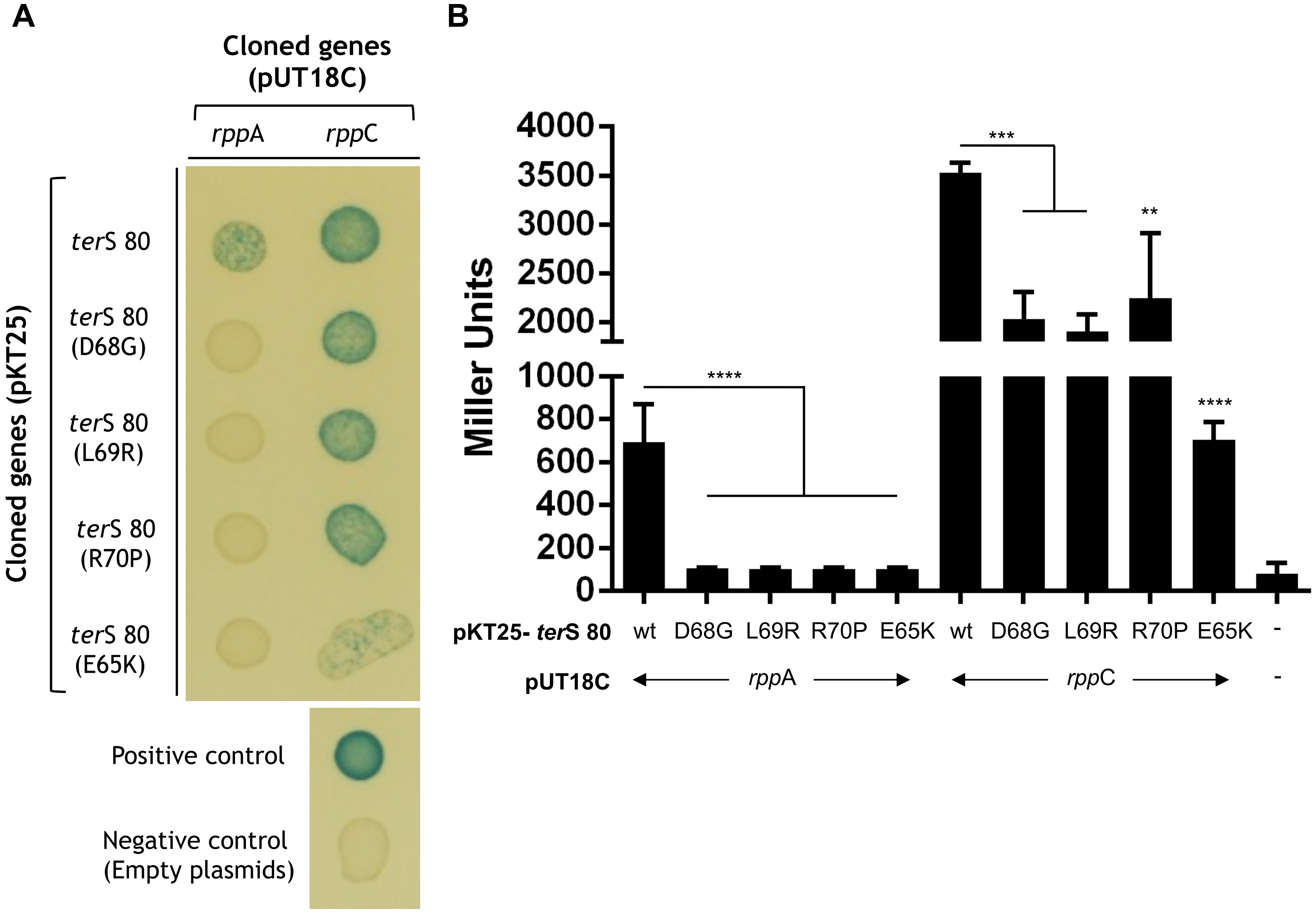

Figure S4. Characterisation of the phage 80 TerS-Rpp interaction. Related to Figures 2 and 3.
(A) BACTH analysis was performed using the plasmid pKT25 encoding different phage 80 *ter*S versions (wt, D68G, L69R, R70P and E65K) and plasmid pUT18C encoding *rpp*A or *rpp*C. Different plasmid combinations are indicated.
(B) Quantification of the BACTH analysis after overnight induction with 0.5mM IPTG. The means of results and standard deviation from three independent experiments are presented (n=3). A 1-way ANOVA with Dunnett's multiple comparisons test was performed to compare mean differences between empty plasmids. Adjusted *p* values were as follows: *rpp*C-*ter*S 80 wt vs *rpp*C-*ter*S 80 D68G p=0.0010***, *rpp*C-*ter*S 80 wt vs *rpp*C-*ter*S 80 L69R p=0.0005***, *rpp*C-*ter*S 80 wt vs *rpp*C-*ter*S 80 R70P p=0.0029**, p<0.0001****.

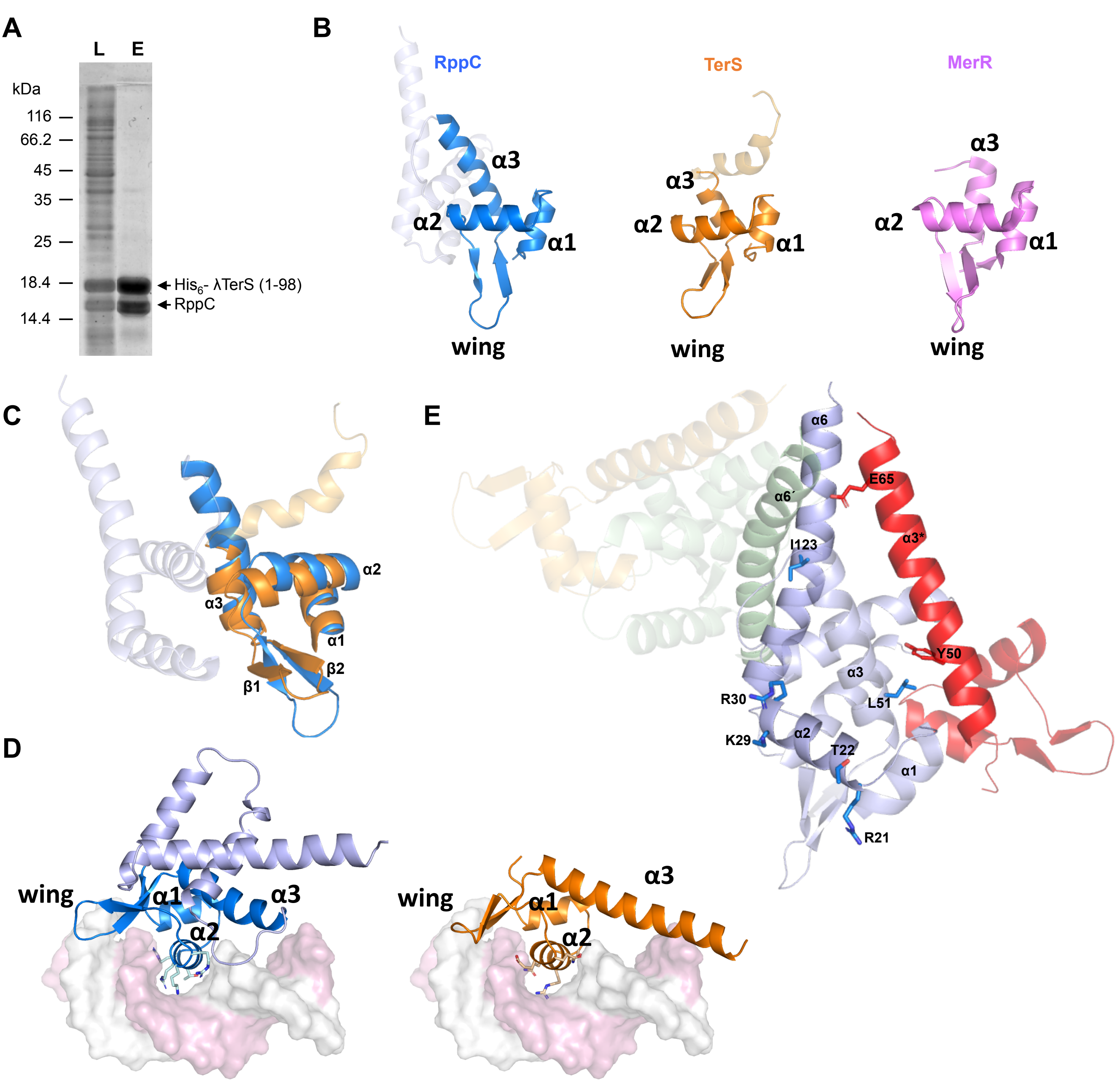

Figure S5. Characterisation of the RppA-TerS interaction. Related to Figures 2, 4, 5 and 6.

(A) RppC interacts with λ TerS (1-98). Affinity chromatography of untagged RppC using His6–λ TerS (residues 1-98). The presence of the different proteins was monitored in the load (lane L), flow-through, wash and elute (lane E) fractions by Coomassie staining.

(B) RppC DBD presents a canonical winged helix-turn-helix DNA binding domain similar to that present in the TerS DBD and in other DNA binding proteins as the MerR transcriptional regulator (PDB 6ama). The wHTH motif from each protein is highlighted in dark tone and the secondary structural elements are labelled in order from N- to C-terminus.

(C) Superimposition of the DNA binding motifs from RppC (blue) and λ TerS DBD (orange) shows identical folding.

(D) Model of RppC-DNA and λTerS-DNA complexes. Cartoon representation of RppC-DNA (left) and λTerS-DNA (right) models. RppC and $\lambda TerS^{1-98}$ are coloured in blue and orange, respectively, with the DBD highlighted in dark tone. Secondary structural elements of the DBDs are labelled in order from N- to C-terminus. The DNA binding models show a common DNA recognition and binding strategy with the helix 2 inserting into the DNA major groove and the wing in the minor groove. The RppC residues placed in these facing secondary structural elements are responsible of the specific PICI *cos*B sequence recognition (R21, T22, R25, K29, R30, from α2, represented in stick, and residues K39 and K41 from the wing), while the equivalent residues present in the λ TerS are involved in the specific recognition of the phage *cos*B site.

(E) Location of the mutated residues in the RppC-$\lambda TerS^{1-98}$ heterocomplex structure. Cartoon representation of the RppC-$\lambda TerS^{1-98}$ heterocomplex. RppC protomers are coloured in green and blue, respectively. The $\lambda TerS^{1-98}$ protomers are in red and yellow, respectively. Key secondary structural elements are labelled in order from N- to C-terminus, the apostrophe (´) indicates the second RppC molecule and the asterisk (*) the λTerS. Mutated residues are labelled and shown in stick representation, with carbon atoms coloured according to the protomer to which they belong. Nitrogen, oxygen and phosphorous atoms are coloured in dark blue, red and orange, respectively.

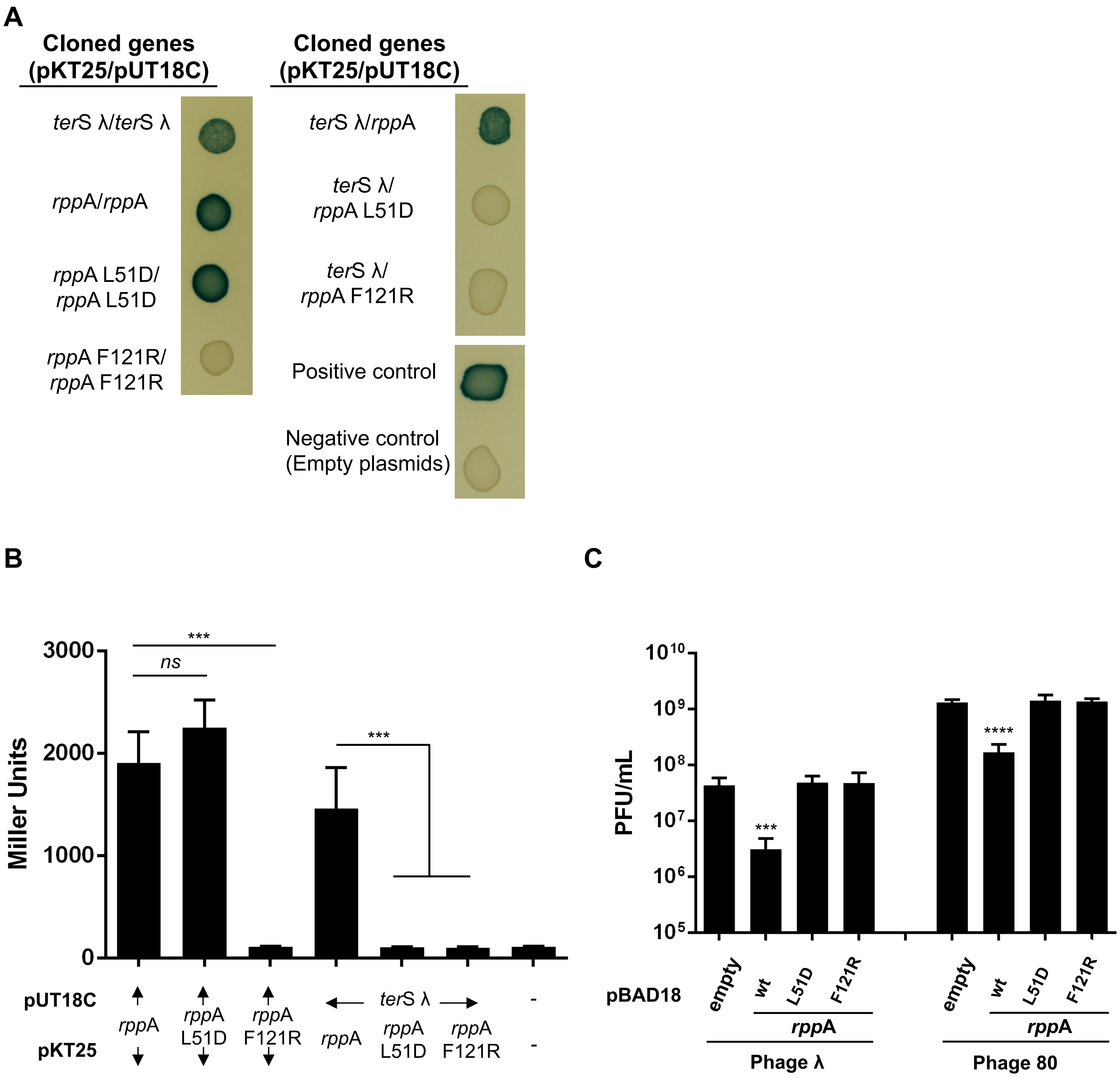

Figure S6. Characterisation of the RppA L51D and F121R mutants. Related to Figures 3 and 5.

(A) BACTH analysis was performed using derivative plasmids pKT25 and pUT18C expressing either the λ TerS or the different RppA mutants. Different plasmid combinations are indicated.

(B) Quantification of the BACTH analysis after overnight induction with 0.5mM IPTG. The means of results and standard deviation from three independent experiments are presented (n=3). A 1-way ANOVA with Dunnett's multiple comparisons test was performed to compare mean differences between *rpp*A and *rpp*A versions or *ter*S λ-*rpp*A and *ter*S λ-different *rpp*A versions. Adjusted *p* values were as follows: *rpp*A-*rpp*A vs *rpp*A F121R-*rpp*A F121R p=0.0002***, *ter*S-*rpp*A vs *ter*S-*rpp*A L51D/F121R p=0.0007***. *ns*, not significant.

(C) The RppA mutants do not block phage reproduction. *E. coli* strains expressing different RppA mutant proteins were infected with phages λ 80 and plated on phage base agar plates supplemented with 0.1% arabinose using phage top agar. Plates were incubated for 24 h at 37°C, and the number of phage plaques quantified. The means of results and standard deviation from three independent experiments are presented (n=3). A 1-way ANOVA with Dunnett's multiple comparisons test was performed to compare mean differences between pBAD18 empty row. Adjusted *p* values were as follows: p=0.0003***, p<0.0001****.

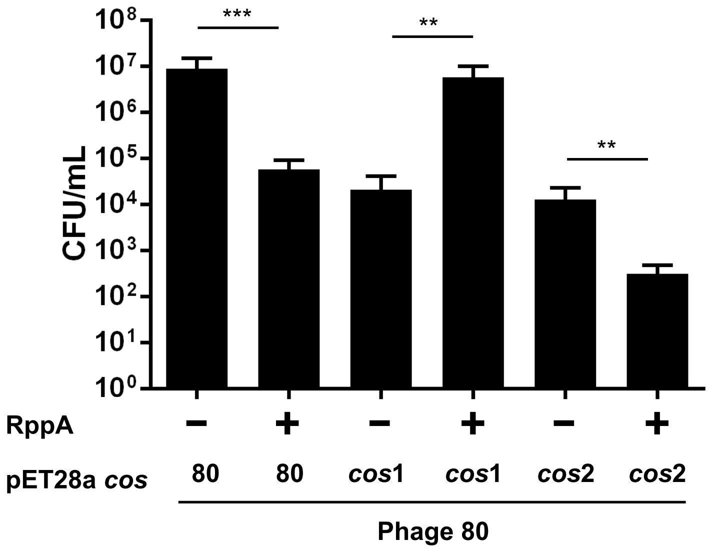

Figure S7. RppA promotes EcCICFT073 *cos*1 recognition. Related to Figure 6.
pET28a derivative plasmids, containing different *cos* sequences (80, *cos*1 or *cos*2) were introduced into the lysogenic strains for phage 80. The strains were MC induced (2 µg/ml) and the transfer of the plasmids analysed in presence or absence of RppA. Expression of RppA from plasmid pBAD18-15A was induced using 0.02% arabinose when appropriate. The figure shows the number of transductants/mL of lysate, using *E. coli* WG5 as recipient strain. The means of results and standard deviation from three independent experiments are presented (n=3). An unpaired *t*-test was performed to compare mean differences of each pET28a *cos* plasmid in presence (+) or absence (-) or *rpp*A. Adjusted *p* values were as follows: Phage 80 pET28a *cos* 80 (+) vs (-) p=0.0006***, Phage 80 pET28a *cos*1 (+) vs (-) p=0.0014**, Phage 80 pET28a *cos*2 (+) vs (-) p=0.0033**.

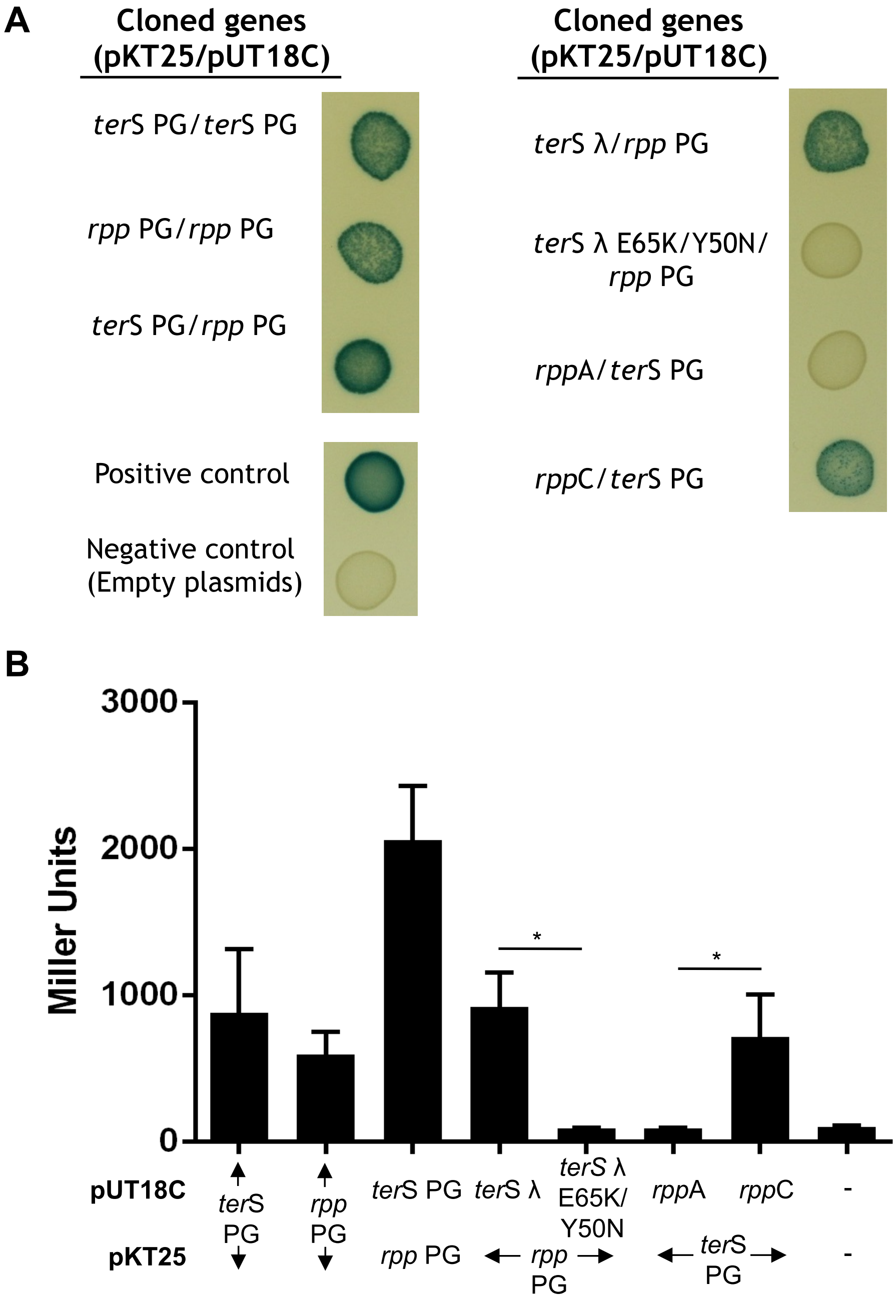

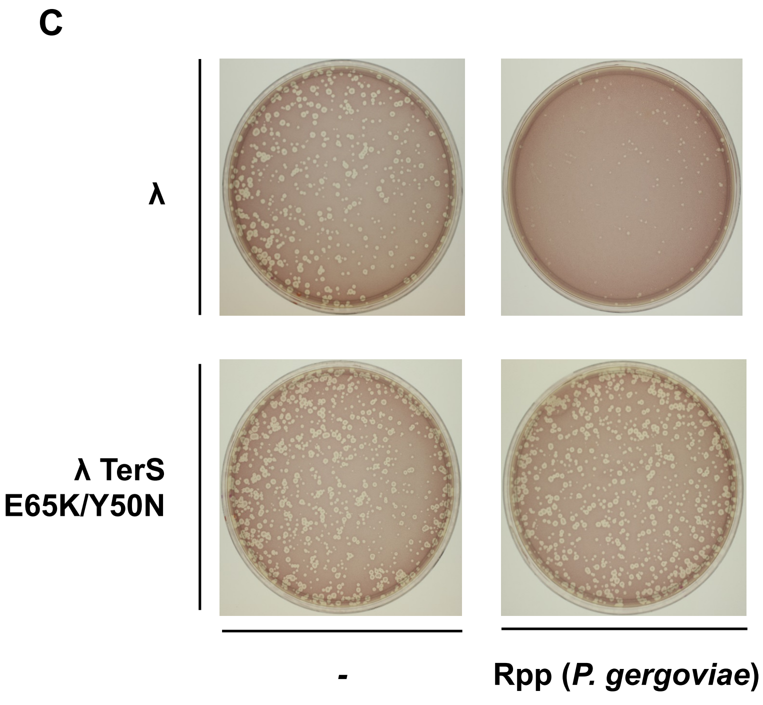

Figure S8. Testing the Rpp-TerS interaction in *Pluralibacter gergoviae*. Related to Figures 2 and 5.

(A) BACTH analysis was performed using the plasmids pKT25 and pUT18C encoding *P. gergoviae ter*S (KMK30155.1) and *rpp* (WP_086499225) or *E. coli ter*S λ, *rpp*A or *rpp*C. Different plasmid combinations are indicated.

(B) Quantification of the BACTH analysis after overnight induction with 0.5mM of IPTG. The means of results and standard deviation from three independent experiments are presented (n=3). An unpaired t-test was performed to compare mean differences within rows. Adjusted *p* values were as follows: *ter*S λ-*rpp* PG vs *ter*S λ E65K/Y50N-*rpp* PG *p*=0.0108*, *rpp*A-*ter*S PG vs *rpp*C-*ter*S PG *p*=0.0204*.

(C) Rpp from *Pluralibacter gergoviae* interferes with λ reproduction. *E. coli* strains JP13131 (594 pBAD18 empty) or JP18876 (594 pBAD18 *rpp* PG) were infected with phage λ, plated on phage bottom agar and incubated for 24 h at 37 °C. Plates were stained with 0.1% (w/v) TTC in LB and photographed.

Table S1. Identification of Rpp proteins in Gram-negative PICIs. Related to Figures 1 and S1.

Specie	Strain	Accession number
E. coli	CFT073	AAN79972.1[a]
E. coli	K1516	EZB64746.1
E. coli	O42	CBG34579.1[a]
E. coli	upec-187	WP_033562019
E. coli	EC2733.1	WP_072135240.1[b]
Shigella boydii	Sb277	ABB66699.1
Shigella dysenteriae	DMB SH20201	RIE73627.1
Shigella flexneri	CDC 796-83 SGF	EFW59515.1
Shigella sonnei	sh1475	SJK45406.1
Salmonella enterica	BCW_2636	WP_080171664.1
Pluralibacter gergoviae	MGH173	OUF48099.1

[a]The protein deposited in the GenBank lacks the first 31 residues.
[b]The protein deposited in the GenBank lacks the first 64 residues.

Table S2. Intersubunit interactions in the RppC dimer. Related to Figure 4.

Homodimer RppC						
RppC subunit A			RppC subunit B			
Structural element	Residue	Atom type	Structural element	Residue	Atom type	Distance
α3	63(LEU)	CA	α6	133(ARG)	CD	3.84
		C				3.66
		CD2			CZ	3.77
	64(GLY)	C		129(LEU)	CD2	3.64
		O		132(LYS)	NZ	2.62
α6	111(ASP)	CB		114(ALA)	CB	3.92
	114(ALA)	C		115(GLN)	CA	3.90
		CB		111(ASP)	CB	3.92
	115(GLN)	CA		114(ALA)	C	3.90
		CG		118(GLY)	CA	3.96
	118(GLY)	CA		115(GLN)	CG	3.96
				118(GLY)	C	3.97
		C				3.39
					CA	3.97
				119(ARG)		3.75
	119(ARG)	CA		118(GLY)	C	3.75
		CG		121(GLN)	CG	3.84
	121(GLN)	CG		119(ARG)	CG	3.84
	122(GLY)	CA		123(ILE)	CG1	3.91
		C		122(GLY)	C	3.53
	123(ILE)	CG1			CA	3.91
				125(ILE)	CG2	3.94
	125(ILE)	CG2		123(ILE)	CG1	3.94
	126(GLY)	C		126(GLY)	C	3.96
	129(LEU)	CD2		64(GLY)	C	3.64
	130(THR)	CG2		130(THR)	CG2	3.85
				131(LEU)	CD2	3.80
	131(LEU)	CD2		130(THR)	CG2	3.80
				134(LEU)	CD1	3.74
	132(LYS)	NZ	α3	64(GLY)	O	2.62
	133(ARG)	CD		63(LEU)	CA	3.84
					C	3.66
		CZ			CD2	3.77
	134(LEU)	CD1	α6	134(LEU)	CD1	3.55
				131(LEU)	CD2	3.74

Table S3. Intermolecular interactions in the RppC-TerS^{Nter} complex. Related to Figure 5.

Heterocomplex RppC-TerS						
RppC			TerS			
**Structural element**	**Residue**	**Atom type**	**Structural element**	**Residue**	**Atom type**	**Distance**
**α1**	6(PHE)	CD1	**Lα1**	1(MET)	CE	3.88
		CZ				3.97
				3(VAL)	CG1	3.53
			**Lα1-α2**	11(ILE)	CD1	3.74
			**α1**	7(GLN)	CD	3.94
		CE2			CB	3.52
					CG	3.74
					CD	3.98
				11(ILE)	CD1	3.36
					CG1	3.65
		CB				3.97
		CG				3.60
					CD1	3.83
		CD2				3.40
					CG1	3.16
	16(GLN)	NE2	**α3**	43(SER)	OG	2.62
**α2**	47(LEU)	CB	**α1**	11(ILE)	CG2	3.64
					CG	3.57
					CD2	3.74
	51(LEU)	CD2		12(PHE)	CE1	3.61
			**α3**	50(TYR)	CD2	3.72
		CD1				3.90
	52(PHE)	CE1			CZ	3.92
	55(ARG)	CD		51(ALA)	CB	3.80
		NE		54(ASP)	OD1	3.00
	63(LEU)	CD1		58(GLU)	CD	3.78
**α3**	77(HIS)	CE1			CD	3.77
	81(ILE)	CG2		57(ILE)	CG2	3.89
		CD1		61(LYS)	CE	3.91
	82(MET)	CE		22(TRP)	CH2	3.98
	85(MET)	O			NE1	2.72
		CG			CZ2	3.76
		CE		53(ARG)	CG	3.02
					CD	3.62
				57(ILE)	CD1	2.28
		CB		53(ARG)	CZ	3.60
**α4**	95(TYR)	CE1	**α2**	14(ALA)	C	3.67
				15(SER)	CA	3.52
		CE2			CB	3.82
		CZ			CA	3.52
					CB	3.55
**α6**	127(ARG)	NH2	**α3**	61(LYS)	O	2.82
				65(GLU)	OE1	2.97
	133´(ARG)	NE			OE1	2.85
		NH1			OE1	2.65

L = loop

Table S4. Strains used in this study. Related to STAR Methods.

Strain	Description	Reference
594	Laboratory strain	
C600	Laboratory strain	ATCC 23738
WG5	Laboratory strain	
DH5α	Laboratory strain	
BTH101	Bacterial Adenylate CyclaseTwo-hybrid System Kit	Euromedex
BL21 (DE3)	Protein overexpression	Novagen
JP10400	C600 phage lambda lysogen	(Fillol-Salom et al., 2018)
JP12507	594 phage 80 lysogen	(Fillol-Salom et al., 2018)
JP12508	594 phage HK97 lysogen	This work
JP13131	594 pBAD18	This work
JP19328	594 pJP2214	This work
JP19329	594 pJP2215	This work
JP19330	594 pJP2216	This work
JP13132	594 pJP2217	This work
JP13133	594 pJP2218	This work
JP13134	594 pJP2219	This work
JP13135	594 pJP2220	This work
JP13136	594 pJP2221	This work
JP13137	594 pJP2222	This work
JP12677	C600 EcCICFT073-c1501::*tet*A	(Fillol-Salom et al., 2018)
JP13957	C600 EcCICFT073-c1501::*tet*A Δc1503	This work
JP16526	594 pJP2243	This work
JP16528	594 pJP2304	This work
JP12979	C600 lambda evolved-EcCICFT073::*tet*A (*nu*1-V3I)	This work
JP12982	C600 lambda evolved-EcCICFT073::*tet*A (*nu*1-A55V)	This work
JP12993	C600 lambda evolved-pBAD18 *rpp*A (*nu*1-E65K)	This work
JP16575	JP12993 evolved-pBAD18 *rpp*C (*nu*1-E65K/Y50N)	This work
JP13173	594 phage 80 evolved-pBAD18 *rpp*A (1) (gp01-D68G)	This work
JP17583	594 phage 80 evolved-pBAD18 *rpp*A (2) (gp01-L69R)	This work
JP17545	594 phage 80 evolved-pBAD18 *rpp*A (3) (gp01-R70P)	This work
JP16571	594 phage 80 evolved-pBAD18 *rpp*C (gp01-E65K)	This work
JP15009	JP10400 pBAD18	This work
JP15012	JP10400 pJP2218	This work
JP15013	JP12507 pBAD18	This work
JP15016	JP12507 pJP2218	This work
JP19363	BTH101 pJP2225 pJP2224	This work
JP19364	BTH101 pJP2228 pJP2224	This work
JP19365	BTH101 pJP2262 pJP2224	This work
JP19366	BTH101 pJP2225 pJP2244	This work
JP19367	BTH101 pJP2228 pJP2244	This work
JP19368	BTH101 pJP2262 pJP2244	This work
JP19369	BTH101 pKT25-control pUT18C-control	This work
JP19370	BTH101 pKT25 pUT18C	This work
JP19388	BTH101 pJP2225 pJP2250	This work
JP19389	BTH101 pJP2249 pJP2224	This work
JP19390	BTH101 pJP2245 pJP2244	This work
JP19391	BTH101 pJP2249 pJP2244	This work
JP19371	BTH101 pJP2229 pJP2224	This work
JP19372	BTH101 pJP2230 pJP2224	This work
JP19373	BTH101 pJP2231 pJP2224	This work
JP19374	BTH101 pJP2232 pJP2224	This work

Strain	Description	Reference
JP19375	BTH101 pJP2305 pJP2224	This work
JP19376	BTH101 pJP2229 pJP2244	This work
JP19377	BTH101 pJP2230 pJP2244	This work
JP19378	BTH101 pJP2231 pJP2244	This work
JP19379	BTH101 pJP2232 pJP2244	This work
JP19380	BTH101 pJP2305 pJP2244	This work
JP13413	JP10400 EcCICFT073-c1504-07::*cat*	This work
JP15342	JP10400 EcCICFT073-c1504-07::*cat* c1503*	This work
JP13891	JP12507 EcCICFT073-c1504-07::*cat*	This work
JP15377	JP12507 EcCICFT073-c1504-07::*cat* c1503*	This work
JP15293	JP12979 EcCICFT073-c1504-07::*cat*	This work
JP15294	JP12993 EcCICFT073-c1504-07::*cat*	This work
JP15295	JP12982 EcCICFT073-c1504-07::*cat*	This work
JP15325	JP13173 EcCICFT073-c1504-07::*cat*	This work
JP17617	JP17583 EcCICFT073-c1504-07::*cat*	This work
JP17618	JP17545 EcCICFT073-c1504-07::*cat*	This work
JP19394	BTH101 pJP2258 pJP2255	This work
JP19395	BTH101 pJP2259 pJP2256	This work
JP19397	BTH101 pJP2225 pJP2255	This work
JP19398	BTH101 pJP2225 pJP2256	This work
JP19332	594 pJP2252	This work
JP19333	594 pJP2253	This work
JP15839	JP10400 EcCICFT073-c1504-07::*cat* c1503 L51D	This work
JP18258	JP10400 EcCICFT073-c1504-07::*cat* c1503 F121R	This work
JP15961	JP12507 EcCICFT073-c1504-07::*cat* c1503 L51D	This work
JP18084	JP12507 EcCICFT073-c1504-07::*cat* c1503 F121R	This work
JP15994	JP10400 pJP2030 pJP2233	This work
JP15995	JP10400 pJP2030 pJP2234	This work
JP15996	JP10400 pJP2033 pJP2233	This work
JP15997	JP10400 pJP2033 pJP2234	This work
JP15998	JP10400 pJP2034 pJP2233	This work
JP15999	JP10400 pJP2034 pJP2234	This work
JP16012	JP12507 pJP2031 pJP2233	This work
JP16013	JP12507 pJP2031 pJP2234	This work
JP16014	JP12507 pJP2033 pJP2233	This work
JP16015	JP12507 pJP2033 pJP2234	This work
JP16016	JP12507 pJP2034 pJP2233	This work
JP16017	JP12507 pJP2034 pJP2234	This work
JP16000	JP10400 pJP2035 pJP2233	This work
JP16001	JP10400 pJP2035 pJP2234	This work
JP16002	JP10400 pJP2036 pJP2233	This work
JP16003	JP10400 pJP2036 pJP2234	This work
JP19697	JP10400 pJP2033 pJP2311	This work
JP16578	594 phage 80 orf63-orf64:*cat* chimera *cos*B:*cos*1 EcCICFT073 pBAD18	This work
JP16579	594 phage 80 orf63-orf64:*cat* chimera *cos*B:*cos*1 EcCICFT073 pJP2218	This work
JP19401	BTH101 pJP2306 pJP2308	This work
JP19402	BTH101 pJP2306 pJP2309	This work
JP19404	BTH101 pJP2307 pJP2309	This work
JP19405	BTH101 pJP2225 pJP2309	This work
JP19406	BTH101 pJP2262 pJP2309	This work
JP19407	BTH101 pJP2249 pJP2308	This work

Strain	Description	Reference
JP19408	BTH101 pJP2245 pJP2308	This work
JP18876	594 pJP2310	This work

Table S6. Plasmids used in this study. Related to STAR Methods.

Plasmid	Description	Reference
**pET28a**	Km^R. Expression vector	Novagen
**pProEX HTa**	Amp^R. Expression vector	Life Technologies
**pKD46**	Amp^R. Thermosensitive plasmid with Red system of lambda phage	(Datsenko and Wanner, 2000)
**pCP20**	Amp^R. Thermosensitive plasmid with Red system of lambda phage	(Datsenko and Wanner, 2000)
**pWRG717**	Amp^R, *km*R. pBluescript II SK+ derivative, *aph* resistance cassette and I-*Sce*I cleavage site.	(Hoffmann et al., 2017)
**pWRG99**	Amp^R. Thermosensitive plasmid with Red system of lambda phage and I-*Sce*I endonuclease under control of tetracycline-inducible promoter (P_{tetA})	(Blank et al., 2011)
**pUT18C**	Amp^R. Bacterial Adenylate CyclaseTwo-hybrid System Kit	Euromedex
**pKT25**	Km^R. Bacterial Adenylate CyclaseTwo-hybrid System Kit	Euromedex
**pUT18C-control**	Amp^R. Bacterial Adenylate CyclaseTwo-hybrid System Kit	Euromedex
**pKT25-control**	Km^R. Bacterial Adenylate CyclaseTwo-hybrid System Kit	Euromedex
**pBAD18**	Amp^R. Expression vector	(Guzman et al., 1995)
**pJP2233**	pBAD18 derivative, origin of replication 15A. Expression vector. Amp^R	This work
**pJP2214**	pBAD18 c1499 EcCICFT073	This work
**pJP2215**	pBAD18 c1500 EcCICFT073	This work
**pJP2216**	pBAD18 c1501 EcCICFT073	This work
**pJP2217**	pBAD18 c1502 EcCICFT073	This work
**pJP2218**	pBAD18 c1503 EcCICFT073	This work
**pJP2219**	pBAD18 c1504 EcCICFT073	This work
**pJP2220**	pBAD18 c1505 EcCICFT073	This work
**pJP2221**	pBAD18 c1506 EcCICFT073	This work
**pJP2222**	pBAD18 c1507 EcCICFT073	This work
**pJP2304**	pBAD18 *rpp*B	This work
**pJP2243**	pBAD18 *rpp*C	This work
**pJP2225**	pKT25 *ter*S lambda	This work
**pJP2228**	pKT25 *ter*S lambda E65K	This work
**pJP2262**	pKT25 *ter*S lambda E65K/Y50N	This work
**pJP2224**	pUT18C *rpp*A	This work
**pJP2244**	pUT18C *rpp*C	This work
**pJP2249**	pKT25 *rpp*A	This work
**pJP2245**	pKT25 *rpp*C	This work
**pJP2250**	pUT18C *ter*S lambda	This work
**pJP2229**	pKT25 *ter*S phage 80	This work
**pJP2230**	pKT25 *ter*S phage 80 D68G	This work
**pJP2231**	pKT25 *ter*S phage 80 L69R	This work
**pJP2232**	pKT25 *ter*S phage 80 R70P	This work
**pJP2305**	pKT25 *ter*S phage 80 E65K	This work
**pJP2285**	pProEX HTa *rpp*C	This work
**pJP2286**	pProEX HTa *ter*S lambda(1-98) *rpp*C	This work
**pJP2255**	pUT18C *rpp*A L51D	This work

Plasmid	Description	Reference
**pJP2256**	pUT18C *rpp*A F121R	This work
**pJP2258**	pKT25 *rpp*A L51D	This work
**pJP2259**	pKT25 *rpp*A F121R	This work
**pJP2252**	pBAD18 *rpp*A L51D	This work
**pJP2253**	pBAD18 *rpp*A F121R	This work
**pJP2234**	pBAD18 15A *rpp*A	This work
**pJP2030**	pET28a *cos* lambda	(Fillol-Salom A., 2018)
**pJP2031**	pET28a *cos* phage 80	(Fillol-Salom A., 2018)
**pJP2033**	pET28a *cos*1 EcCICFT073	(Fillol-Salom A., 2018)
**pJP2034**	pET28a *cos*2 EcCICFT073	(Fillol-Salom A., 2018)
**pJP2035**	pET28a *cos*1-*cos*B *cos*2 EcCICFT073	This work
**pJP2036**	pET28a *cos*2-*cos*B *cos*1 EcCICFT073	This work
**pJP2311**	pBAD18 15A *rpp*A R21A T22A	This work
**pJP2306**	pKT25 *ter*S *Pluralibacter gergoviae*	This work
**pJP2307**	pKT25 *rpp Pluralibacter gergoviae*	This work
**pJP2308**	pUT18C *ter*S *Pluralibacter gergoviae*	This work
**pJP2309**	pUT18C *rpp Pluralibacter gergoviae*	This work
**pJP2310**	pBAD18 *rpp Pluralibacter gergoviae*	This work
