# Supplementary material for: Hijacking the Hijackers: Escherichia coli Pathogenicity Islands Redirect Helper Phage Packaging for Their Own Benefit
Source: Mol Cell. 2019 Sep 5;75(5):1020–1030.e4. doi: 10.1016/j.molcel.2019.06.017 (PMC6739421; doi:10.1016/j.molcel.2019.06.017)
Supplement: Document S2. Article plus Supplemental Information [file mmc3.pdf]

Article

Molecular Cell

Hijacking the Hijackers: *Escherichia coli* Pathogenicity Islands Redirect Helper Phage Packaging for Their Own Benefit

Graphical Abstract

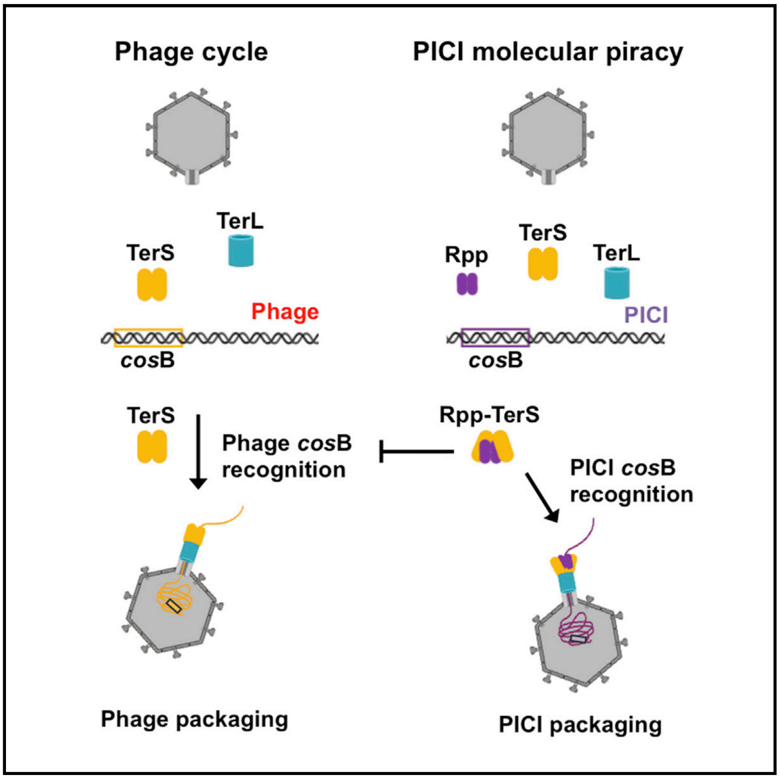

Authors

Alfred Fillol-Salom, Julio Bacarizo, Mohammed Alqasmi, ..., John Chen, Alberto Marina, José R. Penadés

Correspondence

amarina@ibv.csic.es (A.M.),
joser.penades@glasgow.ac.uk (J.R.P.)

In Brief

Fillol-Salom et al. report that Gram-negative PICIs employ the Rpp protein to block helper phage reproduction. Rpp binds to helper phage TerS, and the Rpp-TerS heterocomplex enables PICIs to hijack the phage machinery for their own packaging. These findings reveal a mechanism used by Gram-negative PICIs to spread in nature.

Highlights

- PICI Rpp protein promotes PICI transfer while blocking phage reproduction
- Rpp forms a heterocomplex with helper phage TerS
- Crystal structures of Rpp alone or complexed with TerS were determined
- TerS complexed with Rpp switches specificity from the phage DNA to the PICI genome

Fillol-Salom et al., 2019, Molecular Cell *75*, 1020–1030
September 5, 2019 © 2019 The Authors. Published by Elsevier Inc.
https://doi.org/10.1016/j.molcel.2019.06.017

CellPress

Molecular Cell
Article

Hijacking the Hijackers: *Escherichia coli* Pathogenicity Islands Redirect Helper Phage Packaging for Their Own Benefit

Alfred Fillol-Salom,[1,8] Julio Bacarizo,[1,8] Mohammed Alqasmi,[1,2,8] J. Rafael Ciges-Tomas,[3] Roser Martínez-Rubio,[4] Aleksander W. Roszak,[5] Richard J. Cogdell,[5] John Chen,[6] Alberto Marina,[3,*] and José R. Penadés[1,7,9,*]
[1]Institute of Infection, Immunity and Inflammation, University of Glasgow, Glasgow G12 8TA, UK
[2]College of Applied Medical Sciences, Shaqra University, Shaqra City 15572, Saudi Arabia
[3]Instituto de Biomedicina de Valencia (IBV-CSIC) and CIBER de Enfermedades Raras (CIBERER), Valencia, Spain
[4]Departamento de Ciencias Biomédicas, Universidad CEU Cardenal Herrera, 46113 Moncada, Spain
[5]Institute of Molecular Cell and Systems Biology, University of Glasgow, Glasgow G12 8QQ, UK
[6]Department of Microbiology and Immunology, Yong Loo Lin School of Medicine, National University of Singapore, 5 Science Drive 2, Singapore, Singapore
[7]MRC–University of Glasgow Centre for Virus Research, Glasgow G61 1QH, UK
[8]These authors contributed equally
[9]Lead Contact
*Correspondence: amarina@ibv.csic.es (A.M.), joser.penades@glasgow.ac.uk (J.R.P.)
https://doi.org/10.1016/j.molcel.2019.06.017

SUMMARY

Phage-inducible chromosomal islands (PICIs) represent a novel and universal class of mobile genetic elements, which have broad impact on bacterial virulence. In spite of their relevance, how the Gram-negative PICIs hijack the phage machinery for their own specific packaging and how they block phage reproduction remains to be determined. Using genetic and structural analyses, we solve the mystery here by showing that the Gram-negative PICIs encode a protein that simultaneously performs these processes. This protein, which we have named Rpp (for redirecting phage packaging), interacts with the phage terminase small subunit, forming a heterocomplex. This complex is unable to recognize the phage DNA, blocking phage packaging, but specifically binds to the PICI genome, promoting PICI packaging. Our studies reveal the mechanism of action that allows PICI dissemination in nature, introducing a new paradigm in the understanding of the biology of pathogenicity islands and therefore of bacterial pathogen evolution.

INTRODUCTION

The acquisition of mobile genetic elements (MGEs) that carry virulence factors is a major event that can transform an avirulent or weakly virulent strain into a multi-resistant hypervirulent strain. In spite of the importance of their consequences, the mechanisms underlying the genetic transfer of pathogenicity islands among bacteria remain unidentified in most cases. In recent years, we have described and characterized a new class of chromosomally integrated mobile pathogenicity islands: the phage-inducible chromosomal islands (PICIs) (Penadés and Christie, 2015). The PICIs are widespread among Gram-positive cocci and Gram-negative bacteria (Fillol-Salom et al., 2018; Martínez-Rubio et al., 2017), and they are clinically relevant because they carry and disseminate genes for bacterial superantigens, virulence, and antibiotic resistance (Penadés and Christie, 2015). Following induction by a helper phage, PICIs excise from the bacterial chromosome, replicate, and are packaged into phage-like particles composed of phage virion proteins, leading to very high frequencies of intra- as well as inter-generic transfer (Chen et al., 2015; Chen and Novick, 2009; Maiques et al., 2007).

Although the biology of the Gram-positive PICIs has been extensively studied (Penadés and Christie, 2015), it remains a mystery how the PICI elements present in the Gram-negative bacteria hijack the phage machinery for their preferential packaging and transfer in nature and how these elements interfere with helper phage reproduction. To address these questions, we have analyzed one of these elements, EcCICFT073, present in the uropathogenic *Escherichia coli* CFT073 strain. This PICI raised our curiosity because of its role in virulence and because this element can be mobilized by the archetypical *E. coli* λ and 80 phages (Fillol-Salom et al., 2018). More importantly, our previous results had demonstrated that EcCICFT073 can interfere with phage reproduction using a novel mechanism of phage interference. Although most Gram-positive PICIs interfere with phage reproduction by promoting the formation of small PICI capsids that are much too small for the larger phage genomes (Carpena et al., 2016; Martínez-Rubio et al., 2017; Matos et al., 2013; Quiles-Puchalt et al., 2014; Ruzin et al., 2001; Ubeda et al., 2005), that was not the case for the EcCICFT073 element, which is packaged into phage-sized capsid (Fillol-Salom et al., 2018).

How do *cos* phages, such as λ and 80, package their DNA? The terminase of phage λ is among the best biochemically

 © 2019 The Authors. Published by Elsevier Inc.
This is an open access article under the CC BY license (http://creativecommons.org/licenses/by/4.0/).

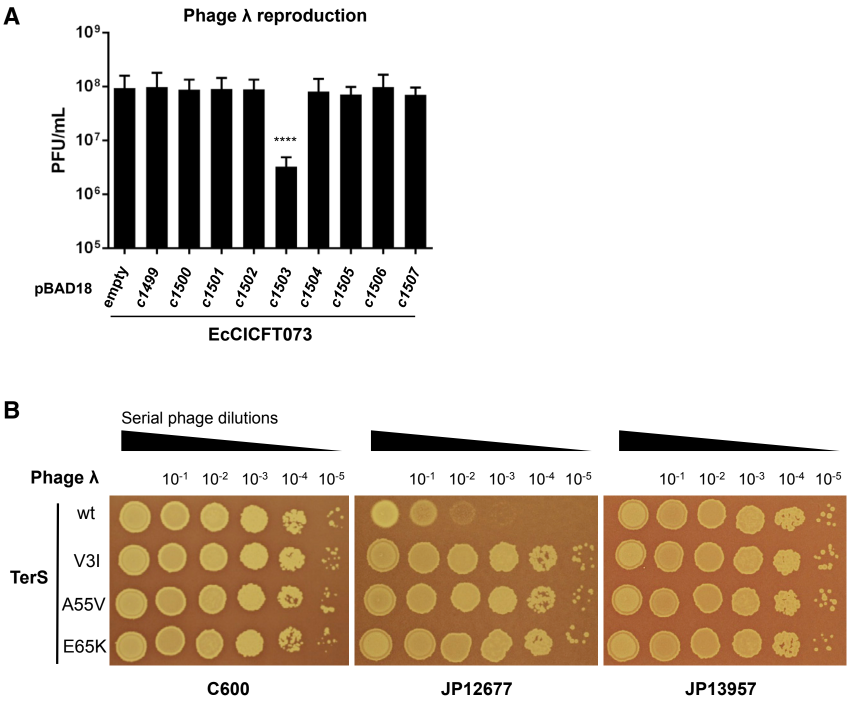

Figure 1. Identification of the EcCICFT073-Encoded Protein Involved in Phage Interference

(A) Phage λ was used to plaque derivatives of non-lysogenic *E. coli* laboratory strain 594 containing pBAD18 expressing different EcCICFT073 proteins. Infected cells were plated on phage base agar supplemented with 0.1% arabinose using phage top agar. The results are represented as the plaque forming units (PFUs) mL^{-1}. The means and SDs are presented (n = 3). A one-way ANOVA with Dunnett's multiple comparisons test was performed to compare mean differences pBAD18 derivatives. Adjusted p values were as follows: ****$p < 0.0001$.

(B) Phage λ dilutions were spotted on non-lysogenic *E. coli* laboratory strain C600, JP12677 (C600 EcCICFT073 *tet*A-positive), or JP13957 (C600 EcCICFT073 *tet*A-positive Δ*c1503*). Plates were stained with 0.1% (w/v) 2,3,5-triphenyltetrazolium chloride (TTC) for enhanced contrast. See also Figures S1 and S2 and Table S1.

characterized proteins that catalyze this process and provides an ideal model for DNA packaging. The enzyme is a hetero-oligomer composed of gpNu1 (also called small terminase [TerS]) and gpA (large terminase [TerL]) subunits. Genome packaging begins with terminase assembly at *cos*, the packaging initiation site in the DNA concatemer. The λ *cos* sequence has three regions required to interact with the packaging machinery: *cos*Q; *cos*N; and *cos*B. Termination of phage packaging requires *cos*Q, and TerL completes this process by cutting the DNA at *cos*N. Initiation of DNA packaging requires both *cos*N and *cos*B sites; *cos*B consists of three binding sites or R elements (R3, R2, and R1) that are required for λ TerS binding to initiate the phage packaging process (Rao and Feiss, 2008; Figure S1).

In a previous study, we found that EcCICFT073 requires the phage-encoded TerS for packaging. We also demonstrated that EcCICFT073 carries two *cos* sites, *cos*1 and *cos*2, with *cos*1 being required for the λ- and 80-mediated transfer of the element (Fillol-Salom et al., 2018). Surprisingly, although both EcCICFT073 *cos* sites have *cos*Q and *cos*N sequences that resemble those present in the *E. coli* λ and 80 phages, we were unable to identify the phage *cos*B element in the EcCICFT073 region (Figure S1A; Fillol-Salom et al., 2018). This observation posed the question, if EcCICFT073 requires the phage machinery for packaging, why does it carry a different *cos*B sequence in its genome, which would be poorly recognized by the phage TerS protein? Does EcCICFT073 encode uncharacterized proteins involved in PICI packaging? And how does EcCICFT073 interfere with phage reproduction? We have unraveled here the mechanism of molecular piracy used by the *E. coli* PICIs to be highly and preferentially packaged and transferred in nature.

RESULTS

Identification of the EcCICFT073-Encoded Inhibitor of Phage λ Reproduction

We initiated this study by identifying the EcCICFT073 gene responsible for blocking λ reproduction. To do that, we individually cloned the EcCICFT073 genes present in the region located after the EcCICFT073 *ori* site into the expression vector pBAD18 (Guzman et al., 1995), under the control of the arabinose-inducible promoter (P_{BAD}), and tested these clones for inhibition of λ reproduction. Note that, in the Gram-positive PICIs, this region usually contains the genes involved in phage interference. Expression of the *c1503* gene, but none of the other genes, dramatically reduced plaque formation by phage λ (Figure 1A). Similar results were obtained with phage 80, but not with phage HK97, which is insensitive to *c1503* (Figure S2A). Deletion of *c1503* fully restored plaque formation and increased λ plaque titers nearly to those seen with the host strain lacking EcCICFT073 (Figure 1B). Based on its function (redirecting phage packaging; see below for more details), the *c1503*-encoded protein was named RppA.

Identification of RppA Homologs

We analyzed the distribution of the *rpp*A gene in the GenBank database and observed that many *E. coli* PICIs encode *rpp*A homologs (Figure S1B), as well as PICIs from other species (Table S1). We next examined whether the other two Rpp homologs found in *E. coli* PICIs, named here RppB and RppC, respectively (Table S1; Figures S1C and S1D), were also able to block phage reproduction. Note that RppA and RppB show 82.64% identity, although RppC shows less identity (43.65%) with RppA and is longer in length (144 residues RppA versus 153 residues

Table 1. Phage Mutants Insensitive to the Rpp-Mediated Interference

	TerS Mutations	
	Phage λ	Phage 80
Target Used to Evolve the Phages		
EcCICFT073::*tet*A	V3I, A55V, E65K	
pBAD18 *rpp*A	E65K	D68G, L69R, R70P
pBAD18 *rpp*C	E65K/Y50N	E65K

RppC; Figures S1C and S1D). The *rpp*B and *rpp*C genes were cloned into the expression vector pBAD18, and the ability of the two encoded proteins to block phage reproduction was tested as previously indicated. As shown in Figure S2B, both RppB and RppC also blocked λ and 80 reproduction.

Identification of the Phage-Encoded Protein Targeted by the Rpp Proteins

We next attempted to identify the stage in the phage reproduction cycle inhibited by RppA. To do this, we introduced the pBAD18 derivative plasmid expressing RppA into the λ and 80 lysogens, and the life cycle of these prophages was induced using mitomycin C (MC). To express RppA, the culture media was supplemented with 0.02% arabinose. Samples containing the lysogenic *E. coli* cells were taken before and 30, 60, 90, and 120 min after MC induction, total DNA was extracted, and the phage replication was analyzed by Southern blotting. In parallel, the impact of RppA on the phage titers was also analyzed. As expected, RppA expression significantly reduced phage titers (Figure S3A), but it did not impact phage replication or phage lysis (Figures S3B and S3C), suggesting that RppA targets phage packaging.

To identify the λ phage protein that was targeted by RppA, we isolated phage mutants able to form plaques on a strain either carrying the EcCICFT073::*tet*A element or expressing the cloned *rpp*A gene. Mutants were readily obtained, and we sequenced the phage genome of 6 of these. In parallel, we also isolated 80 phage mutants insensitive to the RppA interference and sequenced 3 of these mutants. In each case, there was an amino acid substitution in the gene encoding the TerS subunit (Table 1); in four of the six λ TerS mutants, the mutations were at the same site, E65, and in all of these, the glutamic acid was replaced by lysine (E65K; Table 1). Additional mutations were found in the *ter*S gene from phages λ and 80 (Table 1), suggesting that RppA interacts with multiple TerS residues. The identification of TerS as the target explains why the phage HK97 is insensible to RppA; although phages λ and 80 encode a TerS that is practically identical (GenPept: NP_040580 and AFV29141, respectively), the HK97 encoded TerS is completely different in sequence (GenPept: NP_037697).

To know whether RppC also targets the phage TerS, we tried to isolate λ phage mutants insensitive to RppC. Surprisingly, we were unable to isolate a λ mutant capable to form plaques on the RppC-positive strain. We then repeated the selection using phage 80. A single-phage mutant, insensitive both to RppC and RppA, was obtained. This phage carried the TerS E65K mutation (Table 1; Figure S2C). This result confirmed that RppC also targets the phage TerS protein and suggested that the affinity of the RppC protein for the λ TerS is stronger than that observed for the RppA protein. If this was the case, only those λ phages carrying several mutations on the TerS protein would be able to escape to the RppC interference. To test this, we made use of the aforementioned λ TerS E65K mutant, which is insensitive to RppA but still sensitive to RppC (Figure S2D) and evolved it in presence of RppC. In support of this idea, we obtained λ mutants insensitive to RppC; these phages had the double Y50N and E65K mutations in the λ TerS (Table 1; Figure S2D). The fact that the 80 and λ TerS have some differences in sequence explains why one single mutation is enough to avoid RppC interference in phage 80 but two mutations are required in phage lambda.

The previous results suggested that the Rpp proteins interact with the phage-encoded TerS. This was confirmed using a bacterial two-hybrid test, comparing the wild-type (WT) λ TerS and the λ TerS E65K and TerS Y50N/E65K mutants for any interaction with RppA or RppC. As shown in Figure 2, RppA binds strongly to the WT λ TerS, but not to the mutant proteins, and RppC binds even more strongly to the λ TerS and also binds to the λ TerS E65K protein, but not to the λ TerS Y50N/E65K double-mutant protein, confirming that RppC has higher affinity for the λ TerS than RppA. An explanation for this is provided later. Remarkably, both RppA and RppC proteins produce dimers, as does the λ TerS (Figure 2; de Beer et al., 2002). However, RppA and RppC do not interact with each other (Figure 2), suggesting that the different islands interfere with the helper phage, but not with one another. Identical results were obtained when the interaction between the Rpp proteins and the WT and mutant 80 TerS proteins were analyzed (Figure S4). Finally, the interaction between RppC and λ TerS was confirmed by a pull-down assay using His_6-tagged λ TerS (residues 1–98) and untagged RppC (Figure S5A), suggesting that the Rpp proteins interact directly and specifically with the phage-encoded TerS, blocking packaging of the phage DNA.

RppA Is Required for EcCICFT073 Packaging and Transfer

The previous results were unexpected. Because EcCICFT073 requires the helper phage TerS protein for packaging (Fillol-Salom et al., 2018), why does this island express a protein (RppA) that blocks TerS activity? Trying to solve this question, we analyzed the role of RppA in EcCICFT073 transfer. Because RppA blocks TerS and TerS is required for EcCICFT073 transfer, we hypothesized that deletion of *rpp*A would increase EcCICFT073 packaging and transfer by phages λ or 80. This was not the case, and surprisingly, the transfer of the *rpp*A mutant island by phages λ and 80 was significantly reduced compared to the transfer of the WT island (Figure 3A).

We next tested the ability of different λ and 80 TerS mutants, incapable of interacting with the Rpp proteins and consequently insensitive to Rpp-mediated interference (Figures 1B, 2, and S4), to transfer EcCICFT073. As shown in Figure 3B, all the evolved phage mutants showed a reduced capacity to package and transfer the island, suggesting that the Rpp-TerS interaction is essential for EcCICFT073 transfer. Collectively, these results can be summarized as follows: (1) although the phage TerS is

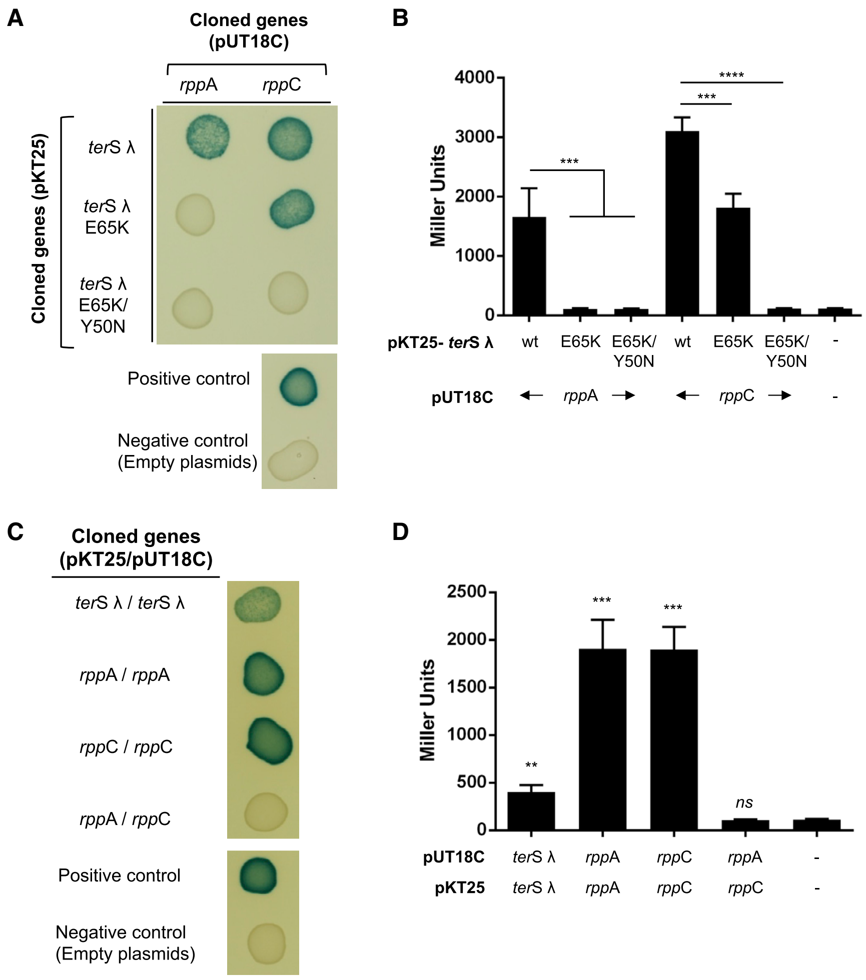

Figure 2. Characterization of the λ TerS-Rpp Interaction

(A) Bacterial adenylate cyclase-based two-hybrid (BACTH) analysis was performed using the plasmid pKT25 encoding different λ TerS versions (WT, E65K, and E65K/Y50N) and plasmid pUT18C encoding RppA or RppC. Plasmid combinations are indicated.

(B) Quantification of the BACTH analysis in (A) after overnight induction with 0.5 mM Isopropyl β-D-1-thiogalactopyranoside (IPTG) measured in Miller units. The means of results and SD are presented (n = 3). A one-way ANOVA with Dunnett's multiple comparisons test was performed to compare mean differences between samples. Adjusted p values were as follows: *rpp*A-WT versus *rpp*A-E65K ***p = 0.0009, *rpp*A-WT versus *rpp*A-E65K ***p = 0.0010, and *rpp*C-WT versus *rpp*C-E65K ***p = 0.0003; ****p < 0.0001.

(C) The Rpp proteins form dimers. BACTH analysis was performed using plasmids pKT25 and pUT18C encoding λ TerS, RppA, or RppC.

(D) Quantification of the BACTH analysis in (C) after overnight induction with 0.5 mM IPTG measured in Miller units. The means of results and SD are presented (n = 3). An unpaired t test was performed to compare dimerization against empty plasmids. Adjusted p values were as follows: **p = 0.0024; *rpp*A ***p = 0.0005, *rpp*C ***p = 0.0002. *ns*, not significant.

See also Figures S2, S3, S4, S5, and S8.

required for EcCICFT073 transfer, this island expresses RppA, a protein that blocks TerS function; (2) in addition to blocking phage packaging, RppA is essential for EcCICFT073 packaging; and (3) to perform its function, RppA must interact with the phage-encoded TerS. Based on these data, the pertinent question was how do the Rpp proteins work?

Structure of RppC

To address this question, we first solved the structure of RppC at 2.4 Å resolution by X-ray crystallography, using the single-wavelength anomalous dispersion (SAD) method (Table 2). The structure showed a single molecule in the asymmetric unit that forms a dimer due to the symmetry of the crystal packing (Figure 4), confirming the RppC oligomerization capacity detected *in vivo* (Figure 2). RppC protomer is composed of six α helices (α1–α6) and two β strands (β1 and β2) that form a long β-hairpin. A DALI search for similar proteins (Holm and Laakso, 2016) revealed that RppC has structural similarities with the MerR transcriptional regulator as well as with other DNA binding proteins, including λ TerS. The structural similarity with these proteins is mainly due to the N-terminal portion (residues 1–64) of RppC that shows a characteristic winged helix-turn-helix (wHTH) DNA-binding fold. This N-terminal DNA binding domain (DBD) includes three α helices (α1–α3) and the β hairpin that correspond to the wHTH wing (Figure S5B). Remarkably, the RppC DBD presents a quite similar fold to that observed for the RMN structure of the N-terminal region of the λ TerS (PDB: 1J9I), showing a root mean square (RMS) deviation of 1.6 Å for the superposition of 51 equivalent C α positions corresponding to this domain (sequence identity 25%) and suggesting that RppC is a DNA binding protein (Figure S5C). Moreover, DALI searches showed structural similarities with the wHTH domain of MerR proteins (root-mean-square deviation [RMSD] of 1.9–3.1 Å for the superimposition of 49–56 C α atoms). MerR family binds to DNA by inserting the recognition helix of the wHTH motif in the major grove and the wing in the minor grove. We modeled the RppC-DNA complex using BldC, a MerR family protein from *Streptomyces*, as a template bound to one of its target promoters (RMSD 2.5 Å for 51 residues superimposed). The model, which was similar to that obtained using other MerR-DNA proteins as templates (data not shown), showed that RppC recognition helix α2 inserts in the DNA major groove with residues R21, T22, R25, K29, and R30 as candidates to direct readout of the DNA, and K39 and K41 projecting from the wing would read out the DNA through the minor groove. Additionally, helix α2 and the wing would also participate in the indirect readout of the operator DNA backbone, suggesting our model of T23, W27, and R43 as candidates to mediate these contacts (Figure S5D). Residues of λ TerS in equivalent positions have been demonstrated to play key roles in the

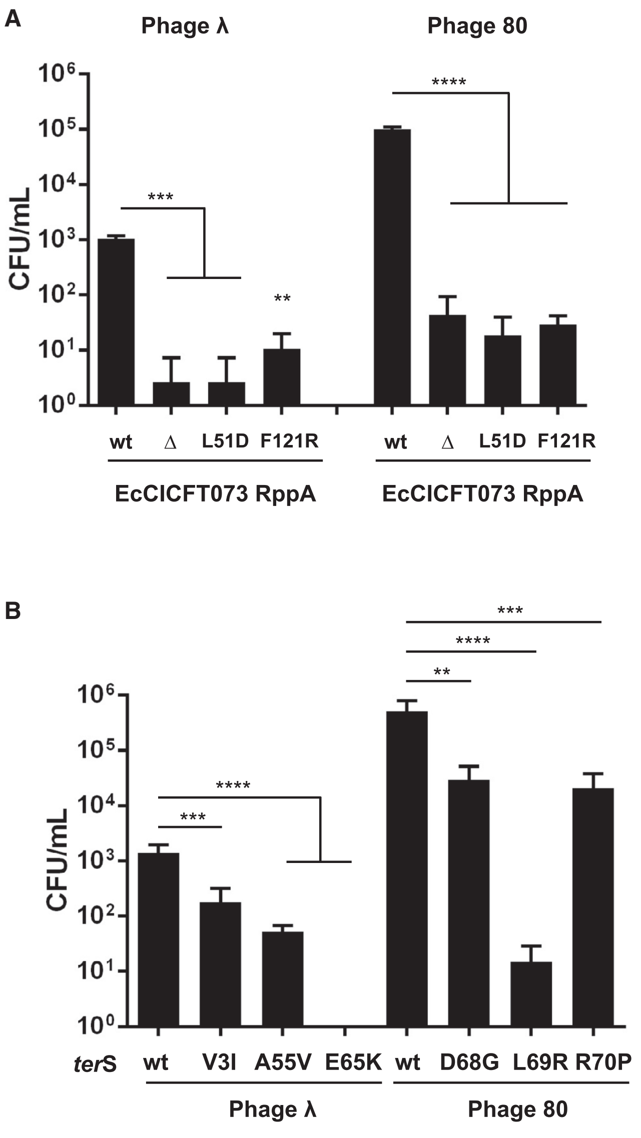

Figure 3. Effect of RppA and TerS Mutations on EcCICFT073 Transfer

(A) Lysogenic strains for phages λ or 80, carrying different versions of the EcCICFT073 island (WT or encoding different RppA mutants), were MC induced (2 μg/mL), and the transfer of the PICI was analyzed using *E. coli* 594 as the recipient strain. The means of the colony forming units (CFUs) and SD are presented (n = 3). A one-way ANOVA with Dunnett's multiple comparisons test was performed to compare mean differences between EcCICFT073-*rpp*A WT. Adjusted p values were as follows: ***p = 0.0005; **p = 0.0018; and ****p < 0.0001.

(B) Lysogenic strains, carrying either WT or mutant λ or 80 prophages, were MC induced in presence of EcCICFT073 and the transfer of the island analyzed using *E. coli* 594 as recipient strain. The means of the CFUs and SD are presented (n = 3). A one-way ANOVA with Dunnett's multiple comparisons test was performed to compare mean differences within between empty plasmids. Adjusted p values were as follows: Lambda TerS WT versus V3I ***p = 0.0001; Phage 80 TerS WT versus D68G **p = 0.0016; and Phage 80 TerS WT versus R70P ***p = 0.0007, ****p < 0.0001.

See also Figures S4 and S6.

recognition of *cosB* R elements (de Beer et al., 2002; Sippy et al., 2015), suggesting a similar DNA-binding mechanism for RppC and λ TerS as could be expected for the conserved DBD fold (Figure S5D). Notably, the DNA-contacting residues proposed by the model are highly conserved among RppA, RppB, and RppC, pointing out that, if Rpps mediate DNA binding, the operator sequence recognized by these three proteins could be similar. In contrast, Rpps should recognize alternative DNA sequences than λ TerS because the residues located at these positions differ between these two types of proteins. This observation would then explain why EcCICFT073 has a different *cos*B region, although it conserves the *cos*N and *cos*Q regions (Fillol-Salom et al., 2018) for λ TerL function.

The remaining C-terminal portion of RppC (residues 65–139) is folded in three α helices (α4–α6) and mediates protein dimerization, especially the long C-terminal α6 helix (residues 110–139) that runs parallel to the α6 helix of the second monomer forming a coiled-coil and providing most of the dimer contacts (Figure 4; Table S2). The dimerization interface buries ~660 $Å^2$ surface area and in helix α6 involves two hydrophobic patches formed by I123, I125, L129, L131, and L134 fringed by polar residues (Q115, R119, Q121, R127 T130, K132, and R133) and, surprisingly for a coiled-coil helix, three Gly residues (G118, G122, and G126) spaced one from each other by one helix turn. Sequence comparison among Rpps showed that residues mediating dimerization are present with low conservation (Figures S1C and S1D), rationalizing the lack of interaction between RppC and RppA observed in our bacterial two-hybrid test assays (Figure 2). Although RppC is a dimer like λ TerS, the available structural data show that both proteins use different surfaces to oligomerize. Although λ TerS does it through the DBD domain, RppC does not use its equivalent domain but rather employs the C-terminal portion.

Structural Basis of Rpp Function

Next, we attempted to solve the structure of the Rpp in complex with the λ TerS. Unfortunately, λ TerS is highly insoluble, which has hindered its structural characterization. In contrast, the N-terminal DBD portion (residues 1–98) forms soluble dimers that have facilitated its study in solution (de Beer et al., 2002; Yang et al., 1999). We produced the N-terminal soluble portion of λ TerS ($TerS^{N\text{-}ter}$ residues 1–98) and confirmed that it maintained its capacity to interact with Rpp (Figure S5A). Importantly, we were able to obtain the crystallographic structure of RppC in complex with the $TerS^{N\text{-}ter}$ at 2.8-Å resolution (Figure 5). The asymmetric unit of the crystal showed a heterodimer composed of single copies of RppC and $TerS^{N\text{-}ter}$. RppC again exploits the crystallographic symmetry to oligomerize, generating a homodimer almost identical to the observed in the crystal structure of RppC alone (RMSD of 0.6 Å for the superimposition of both homodimers). The RppC dimer interacts laterally with two monomers of $TerS^{N\text{-}ter}$ to form a heterocomplex with a $TerS^{N\text{-}ter}$-$RppC_2$-$TerS^{N\text{-}ter}$ tetrameric organization. As in the NMR structure (de Beer et al., 2002), $TerS^{N\text{-}ter}$ presents a wHTH fold consisting of three α helices (α1–α3) and two β strands (β1 and β2) with similar overall docking arrangements seen between the X-ray and NMR structures. However, the X-ray and NMR structures differ in the disposition of their C-terminal segments

Table 2. Data Collection and Refinement Statistics

Data Collection	RppC	RppC-TerS$^{1-98}$
Beamline	DLS I04	ESRF ID30B
Wavelength (Å)	0.9795	0.9762
Space group	$P4_32_12$	$C222_1$
Cell dimensions (Å)	a = 56.88, b = 56.88	a = 60.52, b = 101
	c = 132.27	c = 82.63
	$\alpha = \beta = \gamma = 90$	$\alpha = \beta = \gamma = 90$
Resolution (Å)	43.12–2.42 (2.54–2.4)[a]	50.50–3.00 (3.18–3.00)
Total reflections	46,007 (2,558)	18,007 (3,017)
Unique reflections	7,721 (389)	5,163 (832)
Completeness (%)	86.38 (78)	97.7 (98.7)
Multiplicity	6.0 (6.6)	3.5 (3.6)
Mean I/(σI)	13.6 (1.2)	11.1 (6.7)
Rmerge	0.073 (1.475)	0.064 (0.105)
Rpim	0.033 (0.619)	0.041 (0.066)
CC 1/2	0.999 (0.584)	0.992 (0.992)
Refinement		
Rwork	0.237	0.3124
Rfree	0.267	0.3321
Number of atoms	1,060	1,615
Protein	1,060	1,595
Water	–	20
RMSD, bonds (Å)	0.003	0.016
RMSD, angles (°)	0.679	1.75
Ramachandran Plot		
Preferred (%)	95	86
Allowed (%)	5	14
Outliers (%)	1	0

[a]Number in parentheses indicates values for the highest-resolution cell.

(residues 52–65), which show high mobility in the NMR structure, protruding away from the DBD domain, and in the X-ray structure is stabilized by contacts with RppC and forms part of the long (residues 43–65) α3 helix. Consistent with the mobility of this region observed in NMR experiments, we were unable to trace the 32 C-terminal residues (from 66 to 98) of TerS$^{N\text{-}ter}$ that have been proposed as helical linker between the DBD and the oligomerization domain of λ TerS.

However, the most striking observation from the comparison of TerS homodimer and the RppC-TerS heterodimer structures relates to the disposition of the DBDs in the docking. Superimposition of TerS DBD in both structures shows that RppC DBD occupies the same location as that of the second TerS DBD in the homodimer (Figure 5B). This arrangement indicates that RppC hijacks the phage-packaging machinery by mimicking the DNA-binding portion of TerS to form the dimer. λ TerS DBD homodimerizes by the reciprocal interaction of two patches of residues in the α1 (I11, F12, and G13) and α3 (S43, A44, I47, and A51) helices (Table S3). To form the heterocomplex, RppC not only mimics interactions with these TerS residues but also provides additional interactions between its dimerization domain and the three helices of the TerS DBD. In particular, TerS α3 in its new extended conformation runs parallel to RppC α6, forming the C-terminal portion of these helices into a nascent four-helix bundle in the heterotetramer. These additional interactions will favor the formation of the RppC-TerS heterocomplex over the TerS homodimer. Indeed, *in silico* analysis of both complexes with the PRODIGY server (Xue et al., 2016) predicts a higher binding affinity (ΔG -8.8 versus -6.2 kcal/mol) and dissociation constant ($3.7\ 10^{-7}$ versus $2.8\ 10^{-5}$ M) for the heterocomplex than for the homodimer. Sequence comparison of the Rpp family reveals that residues involved in heterocomplex formation are only partially conserved among Rpps, explaining the differences in affinity for these proteins for TerS.

We can now explain the properties of the λ TerS mutants obtained in the *in vivo* evolutionary experiments. The λ TerS E65 is located in the C-terminal part of the α3 helix, and its mutation to Lys would interfere with the formation of the four-helix bundle in the heterotetramer with Rpp. The TerS Y50 is situated in the main interface used by the DBDs to heterodimerize, and its mutation to Asn would have drastic effects in the complex formation (Figure S5E; Table S3).

Importantly, the λ TerS/RppC structure reveals the strategy used by RppC to perform its dual role: first, because the folding of the DBD formed in the heterodimer is the same as that observed for the λ TerS DBD (Figure 5B), this suggests that it will be functional as a DNA binding domain and should be essential for the recognition of the *cos*B site present in the EcCICFT073 island. As previously mentioned, and because our previous results indicated that the phage-encoded TerS was essential for EcCICFT073 packaging (Fillol-Salom et al., 2018), it was a mystery why this element has a different *cos*B site than its helper phage (Figure S1). Our structural data solve this question. Second, following the formation of the heterodimer DBD, the new DBD formed will have reduced affinity for the λ *cos* site, explaining how Rpp blocks phage packaging.

Functional Characterization of the RppC-TerS Complex

The structural analysis shows that Rpps present two interaction surfaces, one more C-terminal involved in both homo- and heterodimerization and other N-terminal mimicking the TerS DBD used to heterodimerize, and both should be required for Rpp function. In support of this, phages escaping Rpp interference present mutations in λ TerS residues that have a clear impact on the generation of the heterodimer (Figure 2; Table 1). To go further in these studies, and based on the structure of the RppC-λ TerS$^{N\text{-}ter}$ complex, two additional mutants were generated and analyzed in RppA. These correspond to L51D and F121R (Figure S5E). Note that the L51 residue is also conserved in RppC. RppA was used instead of RppC because it was not possible to obtain the EcCIEC2733.1 island encoding RppC, so the impact of the different mutations cannot be analyzed *in vivo* (in a well-defined helper phage-PICI system). In contrast, EcCICFT073 encodes RppA, and this element is mobilized by phages λ and 80 (Fillol-Salom et al., 2018). RppA L51 is placed in one of the two DBD patches that nucleate heterodimerization with TerS and F121 is found in α6 helix, the key structural element in Rpp homodimerization. Thus, the L51D mutation may disrupt heterodimerization with TerS, and the F121R

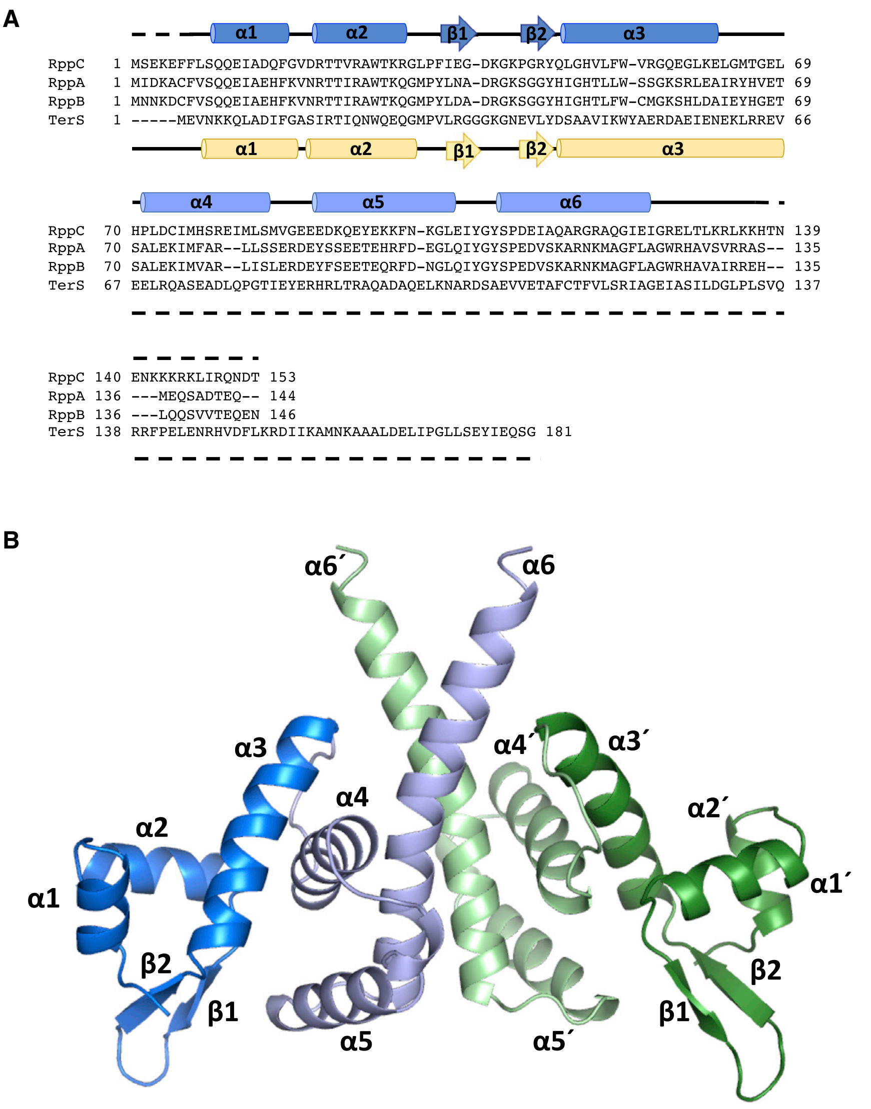

Figure 4. Crystallographic Structure of RppC

(A) Sequence alignment of Rpp proteins and λ TerS. Structural elements of RppC are shown above the sequence colored in blue tones. Structural elements of TerS (PDB: 1J9I) are shown below the sequence colored in yellow.

(B) Cartoon representation of the RppC dimer. Each monomer is colored in blue and green, respectively. DNA binding motifs are highlighted in dark tones. Secondary structural elements are numbered and labeled in order from N to C terminus. The apostrophe (′) indicates the elements from the second protomer.

See also Figure S5 and Table S2.

mutation would disrupt Rpp homodimerization. These predictions were confirmed using the aforementioned bacterial two-hybrid test. Thus, the RppA L51D mutant formed homodimers with RppA but was incapable of interacting with the λ TerS, and the RppA F121R mutant was incapable of forming homodimers (Figures S6A and S6B). Interestingly, F121R had also impaired capacity to intact with the λ TerS (Figures S6A and S6B), indicating that formation of Rpp dimer is essential for interaction with the λ TerS.

We next utilized complementary strategies to validate these mutations *in vivo*. First, we analyzed the ability of phages λ and 80 to infect an *E. coli* strain expressing from plasmid pBAD18 the two RppA mutants. As expected, none of the mutants blocked phage reproduction (Figure S6C). Second, we introduced the different *rpp*A mutations into the EcCICFT073 *cat* element and tested the ability of the different mutant islands to be mobilized by phages λ and 80. As shown in Figure 3A, the transfer of the island encoding the different RppA mutants was significantly reduced. Taken together, these results confirm that both homo- and heterodimer formation are essential for PICI transfer and phage interference.

Our structural data led us to hypothesize that the interaction of Rpp with the phage TerS generates a new DBD that specifically recognizes the EcCICFT073 DNA, but not the λ *cos*B, site. Because λ TerS has shown low-affinity and non-specific DNA binding activity *in vitro* that have forced the use of genetic experiments to dissect its DNA packaging specificity (Frackman et al., 1985; Sippy et al., 2015), we decided to perform additional experiments *in vivo* to test our hypothesis. In the first one, we introduced independently the λ, 80, or each of the two EcCICFT073 *cos* sites (containing the putative *cos*Q, *cos*N, and *cos*B sequences) into plasmid pET28a, which is not transferrable by phages λ or 80, and found that the cloned *cos* sites enabled transfer of the plasmids by these phages (Figures 6A and S7). Consistent with the presence of completely different *cos*B sequences, transfer of the plasmids carrying the EcCICFT073 *cos* sites was reduced compared to that observed with the plasmids carrying the cognate phage *cos* sequences (Figures 6A and S7). We next performed the same experiments but in presence of RppA expressed from plasmid pJP2233, a pBAD derivative plasmid carrying a different origin of replication to avoid plasmid incompatibilities. In support of the proposed model, expression of RppA significantly reduced phage-mediated transfer of the plasmid carrying the phage *cos* sites but significantly increased the transfer of the plasmid carrying the EcCICFT073 *cos*1 site (Figures 6A and S7). This result also explains why the *cos*1 site, but not the *cos*2, is essential for EcCICFT073 transfer. To further demonstrate that the RppA-TerS complex recognizes the EcCICFT073 *cos*B region present in the *cos*1 site, we swapped the *cos*B regions present in the EcCICFT073 *cos*1 and *cos*2 sites and analyzed the ability of these chimeric *cos* sites to be transferred by phage λ in presence of RppA. As shown in Figure 6B, only those plasmids carrying the *cos*B region from the *cos*1 site were transferred in presence of RppA.

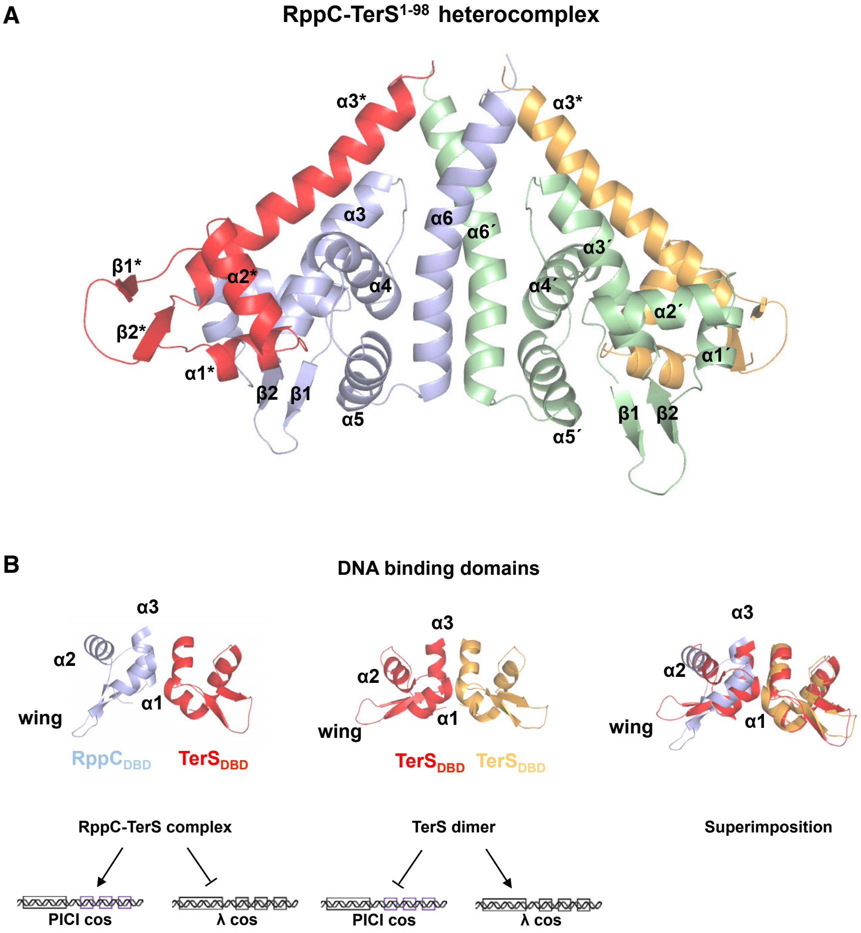

Figure 5. Crystallographic Structure of TerS λ^{1-98} in Complex with RppC

(A) Cartoon representation of the RppC-Terλ^{1-98} heterocomplex. RppC monomers are colored in blue and green. TerS λ^{1-98} monomers are colored in red and yellow. Secondary structural elements are numbered and labeled in order from N to C terminus. The apostrophe (') indicates the elements from the second RppC protomer, whereas TerS structural elements are indicated with asterisks (*).

(B) The DBD structure from the RppC-TerS heterocomplex (left), involved in PICI *cos* recognition, shows identical folding as the TerS DBD (middle), which specifically recognizes the λ *cos*. Superimposition of the previous DBD structures (right) shows quasi-identical disposition of the dimeric DBDs. Secondary structural elements are labeled in the left protomer.

See also Figures S5, S6, and S8 and Table S3.

Our model proposes that RppA interacts specifically with the EcCICFT073 *cos*1 site via the residues in the α2 helix (Figure S5D). To test this hypothesis, we generated a RppA mutant in which the residues R21-T22 were mutated to alanine. Note that these residues are essential components of the α2 helix (Figure 4) and are conserved in both RppB and RppC proteins (Figure S1). Next, the impact of this mutation in both phage interference and in the transfer of the plasmid carrying the EcCICFT073 *cos*1 site was analyzed. In support of our model, the RppA mutant retained its capacity to block phage packaging but was unable to promote the preferential packaging of the island (Figure 6C). Finally, we generated a chimeric 80 prophage in which the *cos*B region from the EcCICFT073 *cos*1 site replaced the phage *cos*B site. This chimeric prophage also contained a *cat* marker, which is used to test lysogenic conversion in *E. coli*. Next, we introduced into the strain lysogenic for the chimeric 80 prophage either the empty plasmid pBAD18 or the pBAD18 derivative expressing RppA. The different strains were induced with MC, the cultures lysed, and the number of lysogens generated in the *E. coli* 594 strain analyzed. In the absence of the Rpp protein, the chimeric 80 phage carrying the EcCICFT073 *cos*B site generated a small number of lysogens (Figure 6D). In contrast, expression of RppA significantly increased packaging and transfer of the chimeric phage, supporting the model that the RppA-TerS complex specifically recognizes the EcCICFT073 *cos*B, but not the phage *cos*B, site.

The Pirating Mechanism Involving Rpp Proteins Is Widespread in Nature

To generalize our results, we analyzed whether the Rpp homologs found in PICIs from different species (Table S1) also work by the same mechanisms as the *E. coli* Rpp proteins. To test this, we selected the Rpp protein from *Pluralibacter gergoviae* (GenPept: WP_086499225) and scrutinized the *P. gergoviae* genomes to find phage-encoded TerS proteins, which would be the target of the Rpp protein. One of these proteins was selected (GenBank: KMK30155.1) and its interaction with the *P. gergoviae* Rpp analyzed, using the two-hybrid system assay. As shown in Figures S8A and S8B, the *P. gergoviae* Rpp forms dimers and interacts with the *P. gergoviae* phage-encoded TerS. Interestingly, the *E. coli* RppC (but not the RppA) is also able to interact with the *P. gergoviae* phage-encoded TerS, and the *P. gergoviae* Rpp is able to interact with the λ TerS, but not with the evolved λ phages carrying mutations in the *ter*S gene (Figures S8A and S8B). Together, these results strongly suggest that all the Rpp proteins are structurally related. In fact, overexpression of the *P. gergoviae* Rpp protein interferes with λ phage reproduction (Figure S8C), confirming the idea that the Rpp proteins have a conserved and widespread mechanism of action. In summary, our results decipher the fascinating mechanism of action that allows the Gram-negative *cos* PICI elements to be packaged and disseminated in nature.

DISCUSSION

Two key features in the PICI lifestyle have been well conserved among all the PICIs analyzed so far: their capacity to interfere

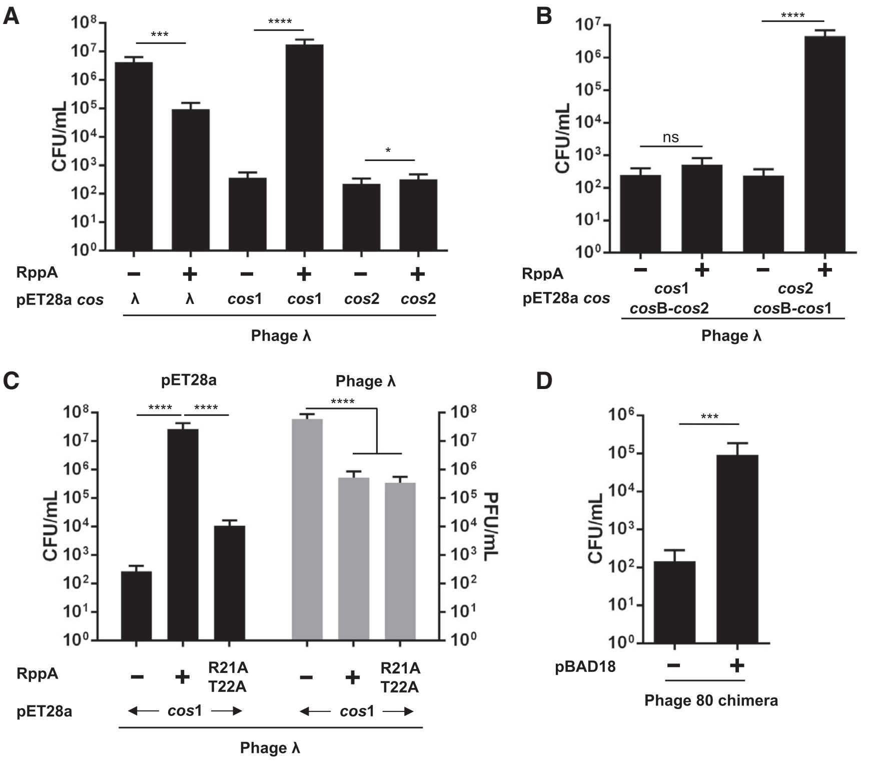

Figure 6. RppA Promotes EcCICFT073 *cos1* Recognition

(A) Strains lysogenic for phage λ containing pET28a with different *cos* sequences (λ, *cos*1, or *cos*2) and pBAD-15A expressing RppA (0.02% arabinose) were MC induced (2 μg/mL) and the transfer of the plasmids analyzed using *E. coli* WG5 as recipient strain. The means of the CFUs and SD are presented (n = 3). An unpaired t test was performed to compare mean differences of each pET28a *cos* plasmid in presence (+) or absence (–) or *rppA*. Adjusted p values were as follows: λ *cos* (+) versus (–) ***p = 0.0002; EcCICFT073 *cos*1 (+) versus (–) ****p < 0.0001; and EcCICFT073 *cos*2 (+) versus (–) *p = 0.04.

(B) RppA promotes recognition of the *cos*B region from the EcCICFT073 *cos*1 site. Strains lysogenic for phage λ containing pET28a with different *cos* chimeric sequences were MC induced and the transfer of the different plasmids analyzed. The means of the CFUs and SD are presented (n = 3). An unpaired t test was performed to compare mean differences of each pET28a *cos* plasmid in presence (+) or absence (–) or *rppA*. Adjusted p values were as follows: ****p < 0.0001.

(C) Strains lysogenic for phage λ containing pET28a with the *cos*1 site and pBAD18-15A expressing RppA WT or RppA R21A T22A were MC induced, and the transfer of the plasmid with the pET28a *cos*1 (left, black bars) or the titer of the λ phage (right, gray bars) was analyzed. The means of results and SD are presented (n = 3). A one-way ANOVA with Dunnett's multiple comparisons test was performed to compare mean differences between samples. Adjusted p values were as follows: ****p < 0.0001.

(D) The strain lysogenic for chimeric phage 80 with the *cos*B region from the EcCICFT073 *cos1* site was MC induced, in presence or absence of RppA, and the transfer of the chimeric phage 80 to recipient *E. coli* 594 was analyzed. The means of CFUs and SD are presented (n = 3). An unpaired t test was performed to compare phage 80 chimera (–) against (+). Adjusted p values were as follows: ***p = 0.0011.

See also Figures S5 and S7.

with phage reproduction and their ability to hijack the phage machinery for their own packaging and transfer. The most conserved strategy of PICI-mediated interference with phage reproduction is the production, using the phage-encoded proteins, of PICI small capsids, which are commensurate to the size of the PICI genomes (Penadés and Christie, 2015). Because PICI genomes are usually 1/3 in size than their helper phages, this strategy impairs packaging of a full-phage genome in the PICI capsids.

However, the production of PICI-sized capsids, although interfering with phage reproduction, does not favor packaging of the PICI element, suggesting PICIs require complementary strategies to increase their transferability in nature. Until now, only one of these strategies had been discovered, used by the prototypical members of the PICI family, the *Staphylococcus aureus* pathogenicity islands (SaPIs). SaPIs that use the headful mechanism for packaging (*pac* SaPIs) encode a homolog of the phage terminase small subunit ($TerS_S$) that specifically recognizes the SaPI genome and directs the packaging of the SaPIs into the SaPI- or phage-sized capsids (Ubeda et al., 2007). To help with this preferential packaging, *pac* SaPIs encode Ppi (for phage packaging interference; Ram et al., 2012), which binds to the phage terminase small subunit ($TerS_P$), but not to the SaPI $TerS_S$, blocking phage $TerS_P$ function. This process would favor SaPI packaging by facilitating the $TerS_S$-TerL interaction, and at the same time, this would block phage packaging by blocking the formation of the $TerS_P$-TerL complex (Ram et al., 2012). However, the exact mechanism by which Ppi performs its function remains to be deciphered.

In contrast to the two-shot strategy (SaPI TerS + Ppi) used by the *pac* SaPIs to promote their transfer, blocking helper phage reproduction, the Gram-negative *cos* PICIs have evolved an elegant one-shot strategy in which the same protein, Rpp, is used to perform both processes. This mechanism explains why the *cos E. coli* PICI has a *cos*B site different to that present in their inducing phages and why this strategy is so efficient in simultaneously performing both processes. Our structural data reveal that Rpps present a DBD domain structurally similar to TerS, suggesting that this protein could substitute for TerS in the *cosB* R elements binding. Because the structural models propose that TerS and Rpps present alternative residues in the key positions for DNA recognition, the R elements of the phage and the PICIs should differ, explaining why λ TerS R elements are not present in *cosB* region of EcCICFT073. Remarkably, the residues involved in DNA recognition seem to show some conservation among Rpps, suggesting that the R elements present are similar. This fact could open the door to a certain degree of promiscuity, and the genomes of nearby related islands could be packaged in different capsids, thus ensuring a high degree of transference.

Does the interaction of Rpp with TerS only aim to deprive the phage of this essential protein required for its own packaging? We do not think this is correct. Although this strategy blocks phage packaging, the Rpp-TerS interaction also allows the recruitment of the phage–packaging machinery. TerS interacts with TerL by its C-terminal portion (residues 100–181; Frackman et al., 1985; Wu et al., 1988). This region is dispensable for the interaction with RppC, as confirmed by the RppC-TerS structure, where it is absent. Indeed, we were unable to trace the 30 C-terminal residues of TerS in the RppC-TerS structure (residues 67–96), which precedes the TerL-interacting region, confirming the independent functions of the TerS domains involved in the RppC or TerL binding. In this scenario, it is tempting to speculate that the TerS C-terminal domains that project from the body of TerS-RppC heterocomplex are free to recruit TerL. Once TerL is recruited, the core of the catalytically component terminase complex is formed (Maluf et al., 2006) and other components of the phage machinery can then be hijacked, completing the elegant one-shot strategy developed by the PICIs to promote their preferential packaging.

Fascinating questions about the evolutionary history of the Gram-negative PICIs are raised by this study. What is the origin of the Rpp proteins? Why do different Rpps exist? Why do some PICIs have two different *cos* sites? The fact that TerS and Rpp share conserved DBD domains, including some sequence identity, suggests that these proteins either have a common ancestor or more likely the Rpp proteins have evolved from TerS. This evolution has generated Rpp proteins that perform some functions (DNA recognition) similarly to TerS. But they only work by forming a complex with TerS, explaining why the Rpp proteins affect phage packaging. This parasitic evolution has also generated Rpp variants, all with the ability to interfere with TerS function but unable to interact and interfere with the activity of the other Rpp proteins. With this strategy, and in the case of a strain containing several PICIs encoding different Rpp proteins, all the PICIs would be able to hijack the phage machinery for packaging without generating Rpp heterodimers that could affect the transfer of the different PICIs. Furthermore, sequence similarities would indicate some packaging promiscuity among PICIs. It is clear for all these scenarios that the PICIs are independently evolving genetic elements that have fine-tuned multiple strategies to spread in nature.

STAR★METHODS

Detailed methods are provided in the online version of this paper and include the following:

- KEY RESOURCES TABLE
- LEAD CONTACT AND MATERIALS AVAILABILITY
- METHOD DETAILS
  - Bacterial Strains and Growth Conditions
  - Induction
  - DNA Methods
  - Plasmid Construction
  - Phage Evolution
  - Southern Blot
  - Two-Hybrid Assay
  - Protein Expression and Purification
  - Protein Crystallization and Data Collection
  - Model Building and Refinement
- QUANTIFICATION AND STATISTICAL ANALYSIS
- DATA AND CODE AVAILABILITY

SUPPLEMENTAL INFORMATION

Supplemental Information can be found online at https://doi.org/10.1016/j.molcel.2019.06.017.

ACKNOWLEDGMENTS

The X-ray diffraction data reported in this work were collected in experiments performed at I04 and ID-30B beamlines at DLS and ESRF synchrotrons, respectively. Preliminary and complementary X-ray diffraction experiments were performed in I03 and I24 beamlines at DLS synchrotron and XALOC beamline at ALBA synchrotron. We thank the staff of the beamlines used at these synchrotrons for assistance in the measurement of the crystals. This work was supported by grant BIO2016-78571-P from the Ministerio de Economía y Competitividad (Spain) to A.M. and grants MR/M003876/1 and MR/S00940X/1 from the Medical Research Council (UK), BB/N002873/1 and BB/S003835/1 from the Biotechnology and Biological Sciences Research Council (BBSRC) (UK), Wellcome Trust 201531/Z/16/Z (UK), and ERC-ADG-2014 proposal no. 670932 Dut-signal from EU to J.R.P.. X-ray diffraction data collection was supported by Diamond Light Source Block Allocation Group (BAG) proposals MX16258 and MX20229 and by Spanish Synchrotron Radiation Facility ALBA Proposal 2018072901. J.R.P. is thankful to the Royal Society and the Wolfson Foundation for providing him support through a Royal Society Wolfson Fellowship.

AUTHOR CONTRIBUTIONS

Conceptualization, A.M. and J.R.P.; methodology, A.F.-S., J.B., M.A., J.R.C.-T., R.M.-R., A.W.R., R.J.C., J.C., A.M., and J.R.P.; investigation, A.F.-S., J.B., M.A., J.R.C.-T., R.M.-R., J.C., A.M., and J.R.P; writing – original draft, A.M. and J.R.P.; funding acquisition, R.J.C., A.M., and J.R.P.; resources, R.J.C., A.M., and J.R.P.; and supervision, A.M. and J.R.P.

DECLARATION OF INTERESTS

The authors declare no competing interests.

Received: April 19, 2019
Revised: May 25, 2019
Accepted: June 13, 2019
Published: July 23, 2019

REFERENCES

Adams, P.D., Afonine, P.V., Bunkóczi, G., Chen, V.B., Davis, I.W., Echols, N., Headd, J.J., Hung, L.-W., Kapral, G.J., Grosse-Kunstleve, R.W., et al. (2010). PHENIX: a comprehensive Python-based system for macromolecular structure solution. Acta Crystallogr. D Biol. Crystallogr. *66*, 213–221.

Battesti, A., and Bouveret, E. (2012). The bacterial two-hybrid system based on adenylate cyclase reconstitution in *Escherichia coli*. Methods *58*, 325–334.

Blank, K., Hensel, M., and Gerlach, R.G. (2011). Rapid and highly efficient method for scarless mutagenesis within the *Salmonella enterica* chromosome. PLoS ONE *6*, e15763.

Carpena, N., Manning, K.A., Dokland, T., Marina, A., and Penadés, J.R. (2016). Convergent evolution of pathogenicity islands in helper *cos* phage interference. Philos. Trans. R. Soc. Lond. B Biol. Sci. *371*, 20150505.

Chen, J., and Novick, R.P. (2009). Phage-mediated intergeneric transfer of toxin genes. Science *323*, 139–141.

Chen, J., Carpena, N., Quiles-Puchalt, N., Ram, G., Novick, R.P., and Penadés, J.R. (2015). Intra- and inter-generic transfer of pathogenicity island-encoded virulence genes by *cos* phages. ISME J. *9*, 1260–1263.

Datsenko, K.A., and Wanner, B.L. (2000). One-step inactivation of chromosomal genes in *Escherichia coli* K-12 using PCR products. Proc. Natl. Acad. Sci. USA *97*, 6640–6645.

de Beer, T., Fang, J., Ortega, M., Yang, Q., Maes, L., Duffy, C., Berton, N., Sippy, J., Overduin, M., Feiss, M., and Catalano, C.E. (2002). Insights into specific DNA recognition during the assembly of a viral genome packaging machine. Mol. Cell *9*, 981–991.

Emsley, P., Lohkamp, B., Scott, W.G., and Cowtan, K. (2010). Features and development of Coot. Acta Crystallogr. D Biol. Crystallogr. *66*, 486–501.

Evans, P.R., and Murshudov, G.N. (2013). How good are my data and what is the resolution? Acta Crystallogr. D Biol. Crystallogr. *69*, 1204–1214.

Fillol-Salom, A., Martínez-Rubio, R., Abdulrahman, R.F., Chen, J., Davies, R., and Penadés, J.R. (2018). Phage-inducible chromosomal islands are ubiquitous within the bacterial universe. ISME J. *12*, 2114–2128.

Frackman, S., Siegele, D.A., and Feiss, M. (1985). The terminase of bacteriophage lambda. Functional domains for *cos*B binding and multimer assembly. J. Mol. Biol. *183*, 225–238.

Guzman, L.M., Belin, D., Carson, M.J., and Beckwith, J. (1995). Tight regulation, modulation, and high-level expression by vectors containing the arabinose PBAD promoter. J. Bacteriol. *177*, 4121–4130.

Hoffmann, S., Schmidt, C., Walter, S., Bender, J.K., and Gerlach, R.G. (2017). Scarless deletion of up to seven methyl-accepting chemotaxis genes with an optimized method highlights key function of CheM in *Salmonella Typhimurium*. PLoS ONE *12*, e0172630.

Holm, L., and Laakso, L.M. (2016). Dali server update. Nucleic Acids Res. *44* (W1), W351–W355.

Maiques, E., Ubeda, C., Tormo, M.A., Ferrer, M.D., Lasa, I., Novick, R.P., and Penadés, J.R. (2007). Role of staphylococcal phage and SaPI integrase in intra- and interspecies SaPI transfer. J. Bacteriol. *189*, 5608–5616.

Maluf, N.K., Gaussier, H., Bogner, E., Feiss, M., and Catalano, C.E. (2006). Assembly of bacteriophage lambda terminase into a viral DNA maturation and packaging machine. Biochemistry *45*, 15259–15268.

Martínez-Rubio, R., Quiles-Puchalt, N., Martí, M., Humphrey, S., Ram, G., Smyth, D., Chen, J., Novick, R.P., and Penadés, J.R. (2017). Phage-inducible islands in the Gram-positive cocci. ISME J. *11*, 1029–1042.

Matos, R.C., Lapaque, N., Rigottier-Gois, L., Debarbieux, L., Meylheuc, T., Gonzalez-Zorn, B., Repoila, F., Lopes, M. de F., and Serror, P. (2013). *Enterococcus faecalis* prophage dynamics and contributions to pathogenic traits. PLoS Genet. *9*, e1003539.

McCoy, A.J., Grosse-Kunstleve, R.W., Adams, P.D., Winn, M.D., Storoni, L.C., and Read, R.J. (2007). Phaser crystallographic software. J. Appl. Cryst. *40*, 658–674.

Murshudov, G.N., Skubák, P., Lebedev, A.A., Pannu, N.S., Steiner, R.A., Nicholls, R.A., Winn, M.D., Long, F., and Vagin, A.A. (2011). REFMAC5 for the refinement of macromolecular crystal structures. Acta Crystallogr. D Biol. Crystallogr. *67*, 355–367.

Penadés, J.R., and Christie, G.E. (2015). The phage-inducible chromosomal islands: a family of highly evolved molecular parasites. Annu. Rev. Virol. *2*, 181–201.

Powell, H.R., Johnson, O., and Leslie, A.G.W. (2013). Autoindexing diffraction images with iMosflm. Acta Crystallogr. D Biol. Crystallogr. *69*, 1195–1203.

Quiles-Puchalt, N., Carpena, N., Alonso, J.C., Novick, R.P., Marina, A., and Penadés, J.R. (2014). Staphylococcal pathogenicity island DNA packaging system involving *cos*-site packaging and phage-encoded HNH endonucleases. Proc. Natl. Acad. Sci. USA *111*, 6016–6021.

Ram, G., Chen, J., Kumar, K., Ross, H.F., Ubeda, C., Damle, P.K., Lane, K.D., Penadés, J.R., Christie, G.E., and Novick, R.P. (2012). Staphylococcal pathogenicity island interference with helper phage reproduction is a paradigm of molecular parasitism. Proc. Natl. Acad. Sci. USA *109*, 16300–16305.

Rao, V.B., and Feiss, M. (2008). The bacteriophage DNA packaging motor. Annu. Rev. Genet. *42*, 647–681.

Ruzin, A., Lindsay, J., and Novick, R.P. (2001). Molecular genetics of SaPI1–a mobile pathogenicity island in *Staphylococcus aureus*. Mol. Microbiol. *41*, 365–377.

Sippy, J., Patel, P., Vahanian, N., Sippy, R., and Feiss, M. (2015). Genetics of critical contacts and clashes in the DNA packaging specificities of bacteriophages λ and 21. Virology *476*, 115–123.

Ubeda, C., Maiques, E., Knecht, E., Lasa, I., Novick, R.P., and Penadés, J.R. (2005). Antibiotic-induced SOS response promotes horizontal dissemination of pathogenicity island-encoded virulence factors in staphylococci. Mol. Microbiol. *56*, 836–844.

Ubeda, C., Maiques, E., Tormo, M.A., Campoy, S., Lasa, I., Barbé, J., Novick, R.P., and Penadés, J.R. (2007). SaPI operon I is required for SaPI packaging and is controlled by LexA. Mol. Microbiol. *65*, 41–50.

Vonrhein, C., Flensburg, C., Keller, P., Sharff, A., Smart, O., Paciorek, W., Womack, T., and Bricogne, G. (2011). Data processing and analysis with the autoPROC toolbox. Acta Crystallogr. D Biol. Crystallogr. *67*, 293–302.

Winn, M.D., Ballard, C.C., Cowtan, K.D., Dodson, E.J., Emsley, P., Evans, P.R., Keegan, R.M., Krissinel, E.B., Leslie, A.G.W., McCoy, A., et al. (2011). Overview of the CCP4 suite and current developments. Acta Crystallogr. D Biol. Crystallogr. *67*, 235–242.

Wu, W.F., Christiansen, S., and Feiss, M. (1988). Domains for protein-protein interactions at the N and C termini of the large subunit of bacteriophage lambda terminase. Genetics *119*, 477–484.

Xue, L.C., Rodrigues, J.P., Kastritis, P.L., Bonvin, A.M., and Vangone, A. (2016). PRODIGY: a web server for predicting the binding affinity of protein-protein complexes. Bioinformatics *32*, 3676–3678.

Yang, Q., Berton, N., Manning, M.C., and Catalano, C.E. (1999). Domain structure of gpNu1, a phage lambda DNA packaging protein. Biochemistry *38*, 14238–14247.

STAR★METHODS

KEY RESOURCES TABLE

REAGENT or RESOURCE	SOURCE	IDENTIFIER
Antibodies		
Anti-Digoxigenin-AP, Fab fragments	Sigma-Aldrich (Roche)	11093274910
Bacterial and Virus Strains		
Bacterial strains, see Table S4	N/A	N/A
Chemicals, Peptides, and Recombinant Proteins		
LB medium	Sigma-Aldrich	L3022
Bacteriological agar	Sigma-Aldrich	A5306; CAS 9002-18-0
Nutrient Broth No. 2	ThermoFisher (Thermo Scientific)	Cat#CM0001
Platinum® Taq DNA Polymerase High Fidelity	ThermoFisher (Invitrogen)	Cat#11304011
DreamTaq DNA Polymerase	ThermoFisher (Thermo Scientific)	Cat#EP0703
2,3,5-Triphenyltetrazolium chloride	Sigma-Aldrich	T8877; CAS 298-96-4
L-(+)-Arabinose	Sigma-Aldrich	A3256; CAS 5328-37-0
IPTG	Sigma-Aldrich	I6758; CAS 367-93-1
Thermo Scientific X-Gal	FisherScientific	10490470
4-Nitrophenyl β-D-galactopyranoside	Sigma-Aldrich	N1252; CAS 3150-24-1
Digoxigenin-11-dUTP, alkali-stable	Sigma-Aldrich (Roche)	11093088910
Lysozyme from hen egg white	Sigma-Aldrich (Roche)	10837059001
Proteinase K from Tritirachium album	Sigma-Aldrich	P2308
Nylon Membranes, positively charged	Sigma-Aldrich (Roche)	11417240001
Anhydrotetracycline hydrochloride	Sigma-Aldrich	37919; CAS 13803-65-1
Ampicillin sodium salt	Sigma-Aldrich	A9518; CAS 69-52-3
Kanamycin Sulfate	Sigma-Aldrich	60615; CAS 70560-51-9
Chloramphenicol	Sigma-Aldrich	C0378; CAS 56-75-7
Tetracycline	Sigma-Aldrich	T3258; CAS 60-54-8
HisPur Ni-NTA Resin	ThermoFisher (Thermo Scientific)	Cat#88221
SelenoMethionine Solution	Molecular Dimensions	Cat#MD12-503B
Mitomycin C	Sigma-Aldrich	M0503; CAS 50-07-7
Crystallization screenings JBS I, JBS II	Jena Biosciences	Cat#CS114-L
Crystallization screening JCSG	Molecular Dimensions	Cat#MD1-40
Critical Commercial Assays		
QIAquick PCR Purification Kit	QIAgen	Cat#28106
QIAprep Spin Miniprep Kit	QIAgen	Cat#27106
Deposited Data		
Atomic coordinates of RppC	This paper	6HLK
Atomic coordinates of the RppC-Terλ$^{1-98}$ heterocomplex	This paper	6HN7
Original data in Mendeley dataset	This paper	https://doi.org/10.17632/m64fw49kr8.2
Oligonucleotides		
Primers used in this study, see Table S5	N/A	N/A
Recombinant DNA		
Plasmids used in this study, see Table S6	N/A	N/A
Software and Algorithms		
GraphPad prism	GraphPad Software	https://www.graphpad.com/scientific-software/prism/
Mosflm	Powell et al., 2013	https://www.mrc-lmb.cam.ac.uk/harry/imosflm/ver722/introduction.html

(*Continued on next page*)

Continued

REAGENT or RESOURCE	SOURCE	IDENTIFIER
Aimless	Evans and Murshudov, 2013	http://www.ccp4.ac.uk/dist/html/aimless.html
Phenix suite	Adams et al., 2010	http://www.phenix-online.org/
CCP4 suite	Winn et al., 2011	http://www.ccp4.ac.uk/
Phaser	McCoy et al., 2007	http://www.ccp4.ac.uk/html/phaser.html
Refmac	Murshudov et al., 2011	http://www.ccp4.ac.uk/html/refmac5.html
Coot	Emsley et al., 2010	https://www2.mrc-lmb.cam.ac.uk/personal/pemsley/coot/

LEAD CONTACT AND MATERIALS AVAILABILITY

Further information and requests for reagents should be directed to Lead Contact José R Penadés (joser.penades@glasgow.ac.uk).

METHOD DETAILS

Bacterial Strains and Growth Conditions

Bacterial strains used in this study are listed in Table S4. Strains were grown at 37°C or 30°C on Luria-Bertani (LB) agar or in LB broth with shaking (180 rpm). Ampicillin (100 μg ml^{-1}), Kanamycin (30 μg ml^{-1}), Chloramphenicol (20 μg ml^{-1}) or Tetracycline (20 μg ml^{-1}; all Sigma-Aldrich), were added when appropriate.

Induction

Bacteria were grown in LB broth to OD_{600} = 0.2 and induced by adding mitomycin C (2 μg ml^{-1}). Cultures were grown at 32°C with gentle shaking (80 rpm). Generally, cell lysis occurred 4-5 h post-induction. The number of phage particles in a lysate was quantified using the titering assay. A 1:50 dilution (in fresh LB broth) of an overnight culture of the appropriate *E. coli* recipient strain was prepared and grown until OD600 = 0.3-0.4 was reached. Strains were infected using 50 μL of the recipient culture with the addition of 100 μL of phage lysate serial dilutions, prepared with phage buffer, and incubated for 5 min at room temperature. The different mixtures of culture-phage dilution were plated out on phage base agar plates (PBA; 25 g of Nutrient Broth No. 2, Oxoid; 7g agar) supplemented with $CaCl_2$ to a final concentration of 10mM. PBA plates were kept at room temperature to set up and, afterward, were incubated at 37°C for 24 h. The number of plaques formed (phage particles present in the lysate) were counted and the plaque forming units (PFU) estimated. PBA plates were stained to enhance plaque visibility in the images taken. At least, 6 mL of LB supplemented with 0.1% (w/v) 2,3,5-triphenyltetrazolium chloride (TTC) solution was added per PBA plate and incubated for 30 min at room temperature. Plaques remained unstained due to only living bacteria being able to reduce TTC dye to red formazan.

The PICIs or phage 80 derivatives used in this work contained a *tet*A or *cat* antibiotic cassette. These markers allow for selection of the PICI or phage on selective LB plates, supplemented either with 20 μg/ml tetracycline or 20 μg/ml chloramphenicol. In plasmid transduction experiments, plasmids were selected based on their plasmid antibiotic resistance gene. Transduction titering assays were performed in *E. coli* using strain 594 as recipient. A 1:50 dilution of an overnight culture (in fresh LB broth) was prepared and grown until OD_{600} = 1.4 was reached. Strains were infected using 1 mL of the recipient culture with the addition of 100 μL of phage lysate serial dilutions, prepared in phage buffer, and cultures were supplemented with $CaCl_2$ to a final concentration of 4.4 mM before incubation for 30 min at 37°C. This incubation allows the PICI or phage to infect the acceptor strain. The different culture-phage dilutions were plated out on LBA plates containing the PICI-phage-plasmid appropriate antibiotic. LBA plates were kept at room temperature to set up and, afterward, were incubated at 37°C for 24 h. The number of colonies formed (PICI-phage-plasmid particles in a lysate) were counted and the colony forming units (CFU) were estimated.

DNA Methods

Gene insertions or deletions were performed as described (Datsenko and Wanner, 2000). The chloramphenicol (*cat*) or kanamycin resistance (*km*R) makers were amplified by PCR, with primers listed in Table S5, and inserted in the PICI genome using λ Red recombinase-mediated recombination. The PCR product was transformed into the recipient strain harboring plasmid pKD46, which expresses the λ Red recombinase. The insertion of the resistance markers were verified by PCR. Site-directed scarless mutagenesis was performed as described previously (Hoffmann et al., 2017; Blank et al., 2011). The *km*R marker together with an I-*Sce*I recognition restriction site was amplified by PCR, using primers listed in Table S5, and inserted into the recipient strain harboring plasmid pRWG99, which expresses the λ Red recombinase protein. After verification of the insertion by PCR, 80-mer DNA fragments derived from oligonucleotides or PCR products were electroporated into the mutant strain expressing the λ Red recombinase-mediated system. Successful recombinants were selected by expression of I-*Sce*I endonuclease. The different mutants obtained were subsequently verified by PCR and DNA sequencing.

Plasmid Construction

The plasmids used in this study (Table S6) were constructed by cloning PCR products, amplified with the oligonucleotides listed in Table S5 (Sigma-Aldrich), into the appropriate vectors. The cloned plasmids were verified by Sanger sequencing (Eurofins Genomics). Synthetic plasmids were purchased from DC BIOSCIENCES Limited.

Phage Evolution

Phages were evolved to overcome either the plasmid- or the PICI-mediated interference. The phage plaques obtained after infection of the appropriate strains were collected in a tube containing phage buffer (50 mM Tris pH = 8, 1 mM $MgSO_4$, 4 mM $CaCl_2$ and 100 mM NaCl). Tubes were centrifuged at 5000 rpm for 10 min. The supernatant was filtered using a sterile 0.2 μm filter (Minisart® single use syringe filter unit) and the resultant lysate was used in a new round of phage infection. Consecutive rounds of phage infection, collection of the top layer and generation of new lysate, were performed until the phage overcame the mediated-PICI or plasmid interference. Then, single plaques of insensitive phage mutants were selected to generate individual phage lysogenic strains, which were sequenced by whole genome sequencing.

Southern Blot

Following plasmid (0.02% arabinose; Sigma-Aldrich) and phage (mitomycin C; Sigma-Aldrich from *Streptomyces caespitosus*) induction, samples were taken at defined time points and pelleted. Samples were re-suspended in 50 μL lysis buffer (47.5 μL TES-Sucrose and 2.5 μL lysozyme [10 μg ml^{-1}]; Sigma-Aldrich) and incubated at 37°C for 1 h. Then, 55 μL of SDS 2% proteinase K buffer (47.25 μL H_2O, 5.25 μL SDS 20%, 2.5 μL proteinase K [20 mg ml^{-1}], Sigma-Aldrich from *Tritirachium album*) was added to the obtained lysates and incubated at 55°C for 30 min. Lysates were vortexed with 10 μL of 10x loading dye for 1h. Samples were frozen and thawed in cycles of 5 min incubation in dry ice with ethanol and in a water bath at 65°C. This cycle was repeated three times. Chromosomal DNA was separated by agarose gel electrophoresis by running samples on 0.7% agarose gel at 30V, overnight. The DNA was transferred to Nylon membranes (Hybond-N 0.45 mm pore size filters; Amersham Life Science) using standard methods. DNA was detected using a DIG-labeled probe (Digoxigenin-11-dUTP alkali-labile; Roche) and anti-DIG antibody (Anti-Digoxigenin-AP Fab fragments; Roche), before washing and visualization. The primers used to obtain the DIG-labeled probes are listed in Table S5.

Two-Hybrid Assay

The two-hybrid assay for protein-protein interaction was conducted as previously described (Battesti and Bouveret, 2012) using two compatible plasmids; pUT18C and pKT25, expressing the different protein combinations. Both plasmids were co-transformed into *E. coli* BTH101 for the Bacterial Adenylate Cyclase Two Hybrid (BACTH) system and plated on LB supplemented with ampicillin, kanamycin, 0.1 mM of isopropyl-b-D thiogalactopyranoside (IPTG) and X-gal as an indicator. After incubation at 30°C for 24-48 h, the protein-protein interaction was detected by a color change. Blue colonies represent an interaction between the two clones, while white/yellow colonies are negative for any interaction.

For quantification of the BACTH analysis, strains were grown overnight at 37°C in LB medium containing the appropriate antibiotics and 0.5 mM IPTG. Following overnight induction, a 1 mL aliquot of each strain was pelleted. The Miller method was used to measure β-galactosidase activity levels, using ortho-Nitrophenyl-β-galactoside (ONPG; Sigma-Aldrich) as the substrate. Pellets were re-suspended in the same volume of chilled Z buffer (0.06 M $Na_2HPO_4x7H_2O$, 0.04 M $NaH_2PO_4xH_2O$, 0.01 M KCl, 0.001 M $MgSO_4$ and 0.05M β -mercaptoethanol). The OD_{600} of the re-suspended pellets was measured. The re-suspended cells were diluted in Z buffer to 1 mL (0.1 mL cells + 0.9 mL Z buffer) and cells were permeabilized by adding 100 μL chloroform and 50 μL 0.1% SDS. Immediately after, the mix was vortexed and the tubes were equilibrated for 5 min in a 28°C water bath. The reaction was initiated by adding 0.2 mL of ONPG (4 mg/mL). The time of addition was recorded precisely with a timer. Immediately, the mix was vortexed and the tubes were incubated at 28°C in a water bath. When sufficient yellow color was observed, the reaction was stopped by adding 0.5 mL 1 M Na_2CO_3. The time of addition was recorded precisely and the mix was vortexed. Following this, 1 mL of sample was transferred to Eppendorf tubes and centrifuged for 5 minutes at maximum r.p.m and the OD at 420nm and at 550nm for each tube was recorded. The average of at least three independent experiments is shown in Miller units.

Protein Expression and Purification

Proteins were overexpressed from *Escherichia coli* BL21 (DE3) (Novagen) cells transformed with the corresponding expression plasmids (Table S6). Cultures were grown at 37°C in LB medium supplemented with 100 μg ml^{-1} ampicillin to an OD_{600} of 0.5–0.6. Then, protein expression was induced with 1 mM IPTG at 16°C for 16 h. Cells were harvested by centrifugation at 4°C, 4000 rpm for 30 min, resuspended in lysis buffer (100 mM Tris pH = 8, 300 mM NaCl, 1 mM TCEP) and lysed by sonication. The soluble fractions were obtained by centrifugation at 4°C, 15000 rpm for 1h and loaded onto a pre-equilibrated Nickel affinity gravity column (HisPur™ Ni-NTA Resin; Thermo Fisher). After two washes with 20 mM (40x bed volume) and 50 mM imidazole (30x bed volume), the proteins were eluted with lysis buffer containing 250 mM imidazole. The fractions were analyzed by SDS-PAGE and those fractions showing purest protein were selected, concentrated, and stored at −80°C.

For anomalous X-ray diffraction and phasing, RppC was selenomethionine-labeled (SeMet) by expressing the protein in SelenoMethionine Medium Complete (Molecular Dimensions Ltd; MD 12-500), according to the manufacturer instructions, and purified as described previously.

Protein Crystallization and Data Collection

Crystals of the RppC protein or the RppC-λ$TerS^{N-ter}$ heterocomplex were obtained by vapor-diffusion technique using a sitting drop setup at 15°C. Crystallization drops were generated by mixing equal volumes of each protein solution and the corresponding reservoir solution, and were equilibrated against 100-300 μL reservoir solution. SeMet derivative RppC was crystallized at 15 mg ml^{-1} in a reservoir solution of 15% PEG8K, 0.1 M sodium acetate pH = 6. The heterocomplex was crystallized at 10 mg ml^{-1} in a reservoir solution of 2 M ammonium acetate, 0.1 M sodium acetate (pH = 5). The crystals were cryo-protected using 25%–35% of glycerol solution when freezing in liquid nitrogen. X-ray data collection was carried out at 110K. RppC was collected by Single-wavelength anomalous diffraction (SAD) on the I04 beamline at the Diamond Light Source synchrotron radiation facility (DLS; Didcot, UK) at a wavelength of 0.9795Å. X-ray data of RppC-λ$TerS^{N-ter}$ heterocomplex was collected on the beamline ID-30B of the European Synchrotron Radiation Facility (ESRF; Grenoble, France). Data from SeMet-labeled RppC were indexed, integrated, and scaled using the program autoPROC (Vonrhein et al., 2011), whereas the heterocomplex data were processed and reduced with Mosflm (Powell et al., 2013) and Aimless (Evans and Murshudov, 2013) programs. The crystallographic parameters and data-collection statistics are listed in Table 2.

Model Building and Refinement

Solution and refinement of the crystallographic structure of RppC was performed with the *Phenix* suite (Adams et al., 2010). Automated structure solution using SAD phasing technique was carried out on the Autosol pipeline of Phenix, and a total of 4 selenium atoms were localized, which was enough to calculate experimental phasing and model building.

Structure of RppC-λ$TerS^{N-ter}$ was solved using the CCP4 suite (Winn et al., 2011). Phases were obtained by molecular-replacement using Phaser (McCoy et al., 2007). The structure of the RppC monomer (obtained previously) was used as a model, as well as the monomer of the λ TerS DNA binding domain (PDB: 1J9I; (de Beer et al., 2002)). All the final models were generated by iterative cycles of refinement using Refmac (Murshudov et al., 2011) and manual optimization with Coot (Emsley et al., 2010). Data refinement statistics are given in Table 2. Atomic coordinates and structure factors have been deposited in the PDB (Key Resources Table).

QUANTIFICATION AND STATISTICAL ANALYSIS

All statistical analyses were performed as indicated in the figure legends using GraphPad Prism 6.01 software, where n represents the number of independent experiments.

DATA AND CODE AVAILABILITY

Coordinates for atomic structures have been deposited at the RCSB Protein Data Bank (PDB: 6HLK and PDB: 6HN7). The original data and figures have been deposited in Mendeley dataset (https://doi.org/10.17632/m64fw49kr8.2).

Molecular Cell, Volume *75*

Supplemental Information

Hijacking the Hijackers: *Escherichia coli* Pathogenicity Islands Redirect Helper Phage Packaging for Their Own Benefit

Alfred Fillol-Salom, Julio Bacarizo, Mohammed Alqasmi, J. Rafael Ciges-Tomas, Roser Martínez-Rubio, Aleksander W. Roszak, Richard J. Cogdell, John Chen, Alberto Marina, and José R. Penadés

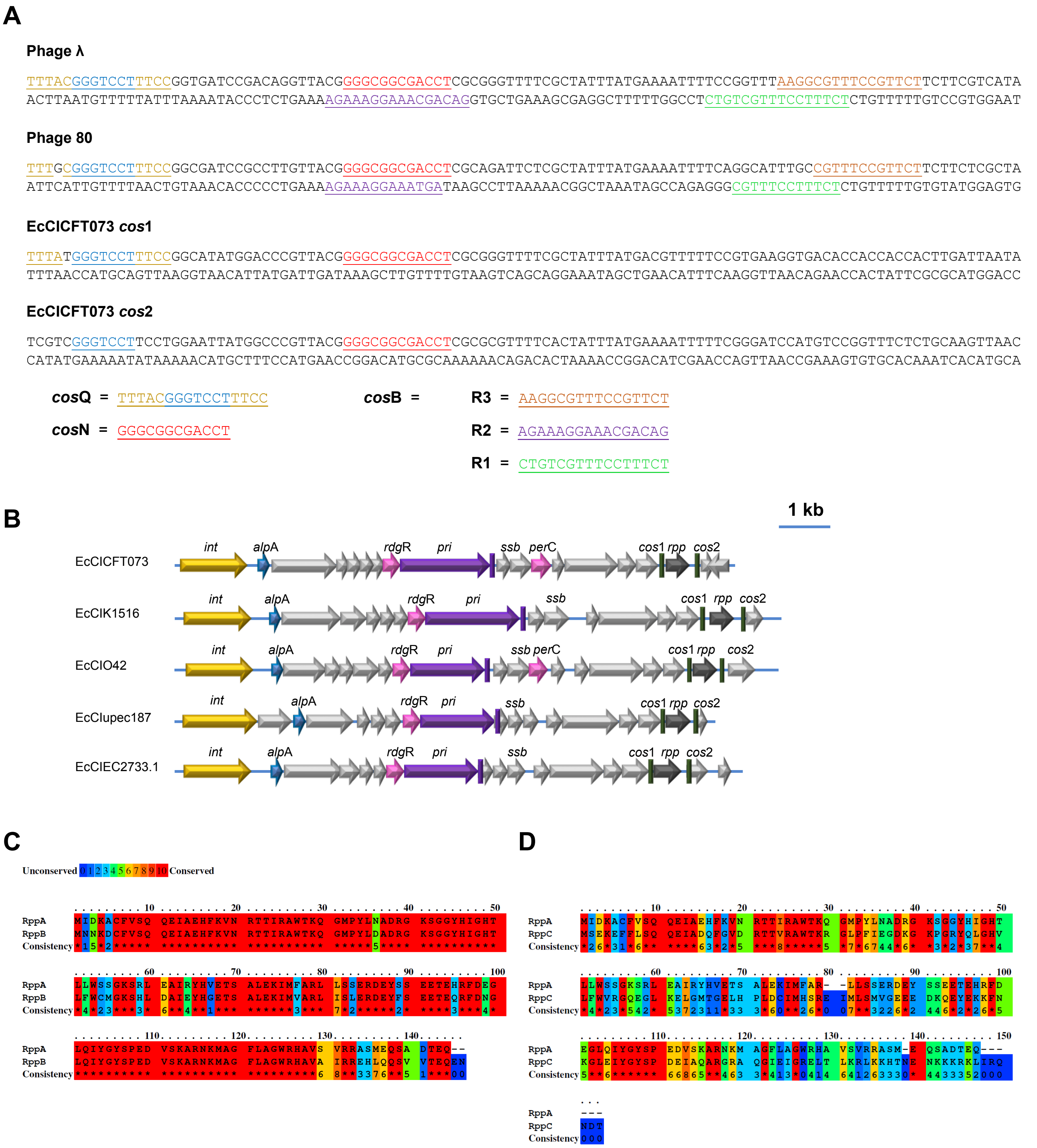

Figure S1. *cos* and Rpp sequences. Related to Figures 1 and S2 and Table S1.
(A) Sequence of the *cos* regions from phages λ and 80 and from the EcCICFT073 element. While all the elements carry the same *cos*Q and *cos*N sequences, the EcCICFT073 PICI *cos*B sequences are completely divergent.
(B) Genome maps for *E. coli* PICIs encoding *rpp* genes. Genomes are aligned according to the prophage convention, with the integrase gene (*int*) at the left end. Genes are coloured according to their function: *int* is yellow; transcription regulator (*alp*A) is dark blue; replication genes (*pri*) are purple; redirecting phage packaging genes (*rpp*) are grey; virulence genes are pink; other accessory genes are red; genes encoding hypothetical proteins are white. *cos* sites are indicated as green rectangles.
(C-D) Rpp homologues in *E. coli* PICIs. Protein sequence alignment of RppA and RppB (C) or RppA and RppC (D), generated using the PRALINE server. Colours indicate relative sequence conservation at each position, with red being most conserved and blue least.

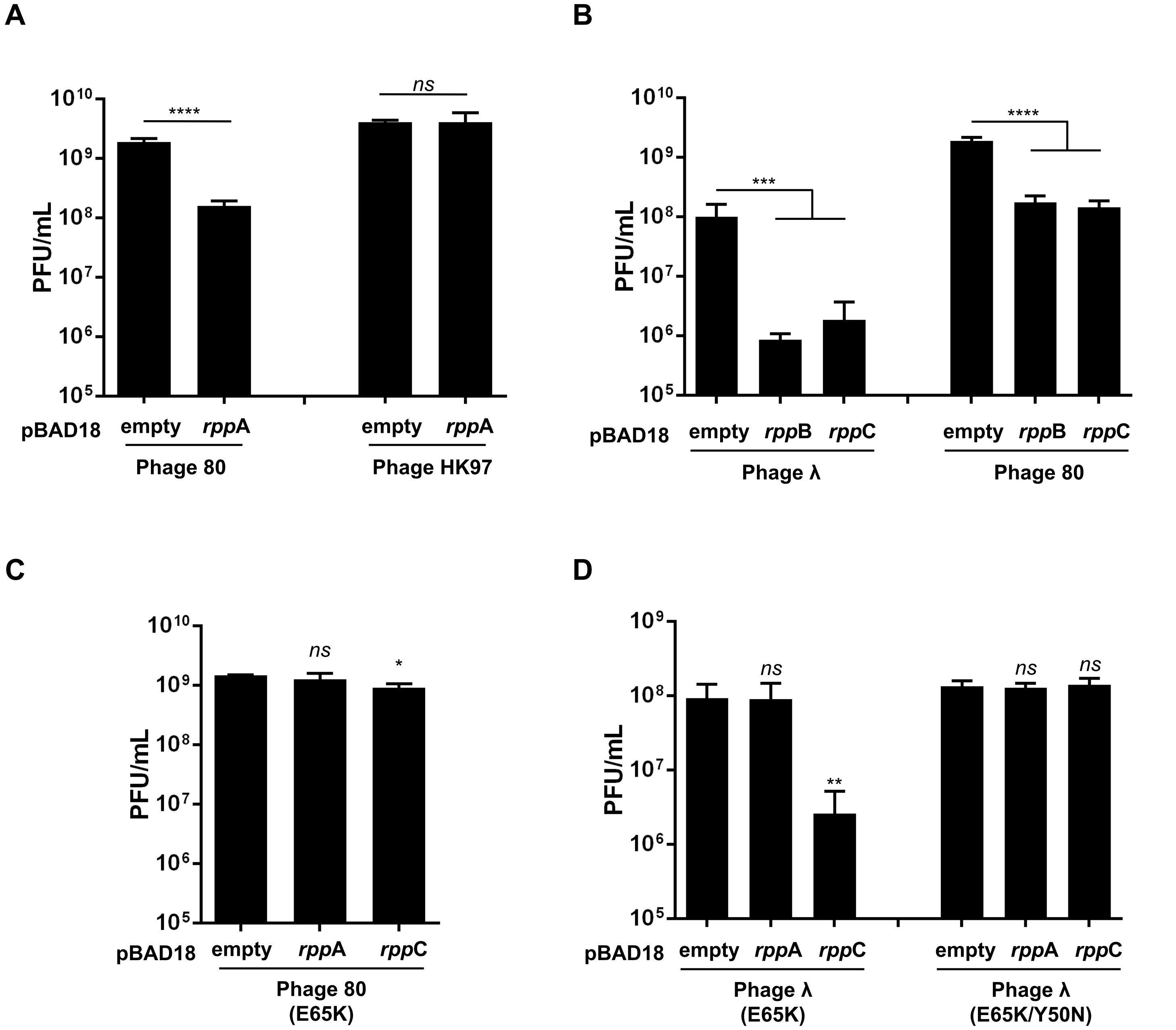

Figure S2. The Rpp homologues block helper phage reproduction. Related to Figures 1, 2 and S1.

(A) *E. coli* strain 594 expressing RppA from plasmid pBAD18 was infected with phages 80 or HK97 and plated on phage base agar plates supplemented with 0.1% arabinose using phage top agar. Plates were incubated for 24 h at 37°C and the number of phage plaques were quantified. The means and standard deviation from three independent experiments are presented (n=3). An unpaired t-test was performed to compare mean differences within rows. Adjusted *p* values were as follows: $p<0.0001^{****}$. *ns*, not significant.

(B) *E. coli* strain 594 containing different pBAD18 derivatives was infected with phage lambda or phage 80 and plated on phage base agar plates supplemented with 0.1% arabinose using phage top agar. Plates were incubated for 24 h at 37°C and the number of phage plaques quantified. The means of results and standard deviation from three independent experiments are presented (n=3). A 1-way ANOVA with Dunnett's multiple comparisons test was performed to compare mean differences between pBAD18 empty row. Adjusted *p* values were as follows: λ: empty vs *rpp*B $p=0.0003^{***}$, empty vs *rpp*C $p=0.0005^{***}$, $p<0.0001^{****}$.

(C-D) Phage 80 and λ mutants insensitive to the RppA and RppC interference. *E. coli* strain 594 containing different pBAD18 derivatives was infected with the evolved phage 80 carrying the TerS E65K mutation (C) or with evolved λ mutants (TerS E65K or E65K/Y50N; D) and plated on phage base agar plates supplemented with 0.1% arabinose using phage top agar. Plates were incubated for 24 h at 37°C. The means of results and standard deviation from three independent experiments are presented (n=3). A 1-way ANOVA with Dunnett's multiple comparisons test was performed to compare mean differences between samples. Adjusted *p* values were as follows: $p=0.0337^{*}$, $p=0.0010^{**}$. *ns*, not significant.

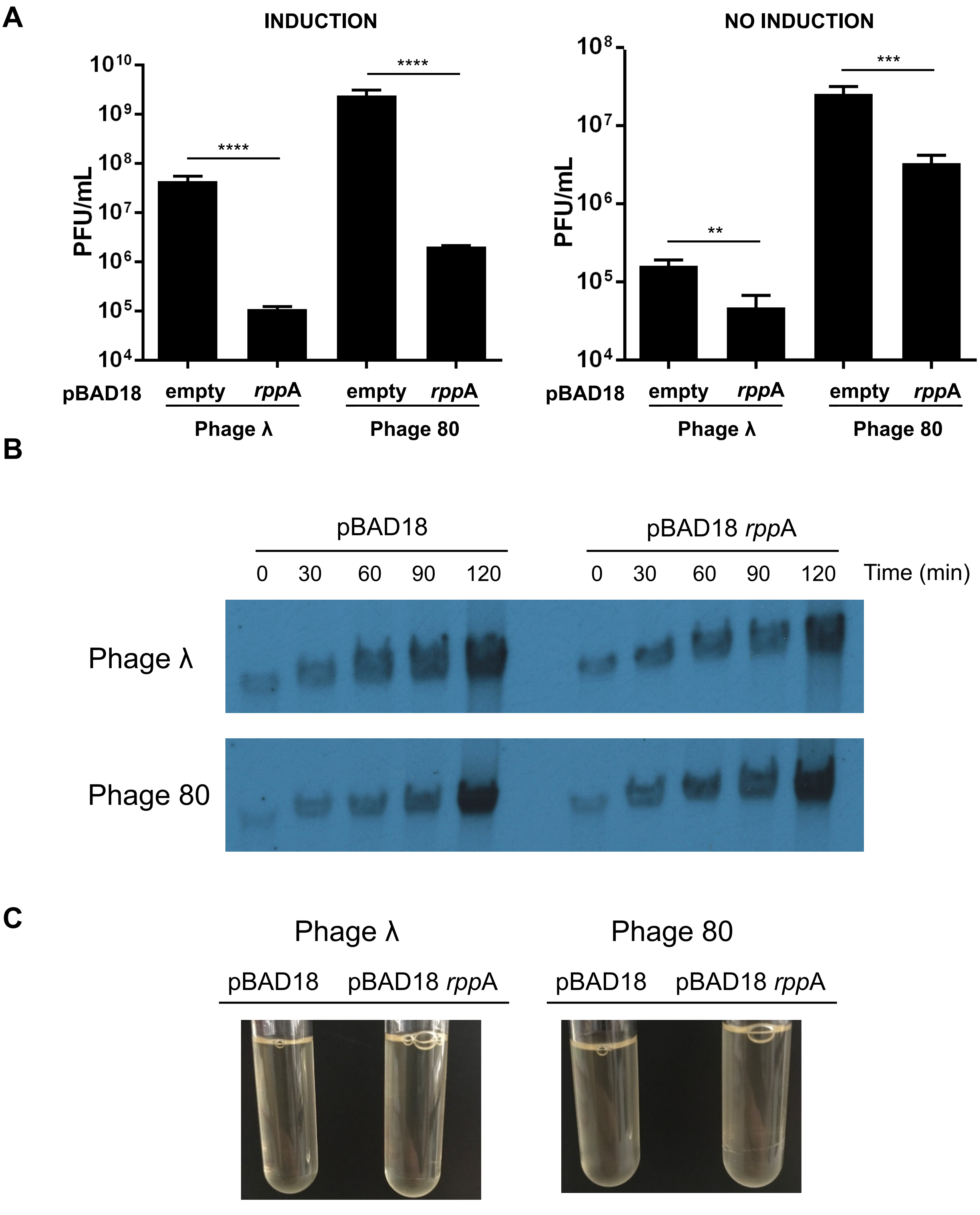

Figure S3. Identification of the Rpp target. Related to Figure 2.

(A) Lysogenic strains for phage λ or 80, carrying plasmid pBAD18 empty or expressing RppA, were induced (left) or not (right) with MC, and the titre of the phage analysed. Expression of RppA from plasmid pBAD18 was induced using 0.02% arabinose when appropriate. The experiment shows the no. of plaques/mL of lysate, using *E. coli* 594 as recipient strain. The means of results and standard deviation from three independent experiments are presented (n=3). An unpaired t-test was performed to compare mean differences within rows. Adjusted *p* values were as follows: *p*<0.0001****, *p*=0.0006***, *p*=0.0079**.

(B) Phage replication after expression of the cloned *rpp*A gene. Lysogenic strains for phage λ or 80 carrying plasmid pBAD18 empty or expressing RppA, were induced and one millilitre of each culture at different time points after induction was collected and used to prepare standard minilysates, which were resolved on a 0.7% agarose gel, Southern blotted and probed for phage DNA.

(C) RppA does not affect phage lysis. Lysogenic strains for for phage λ or 80, carrying plasmid pBAD18 empty or expressing RppA, were MC induced and photographed 6 h after induction.

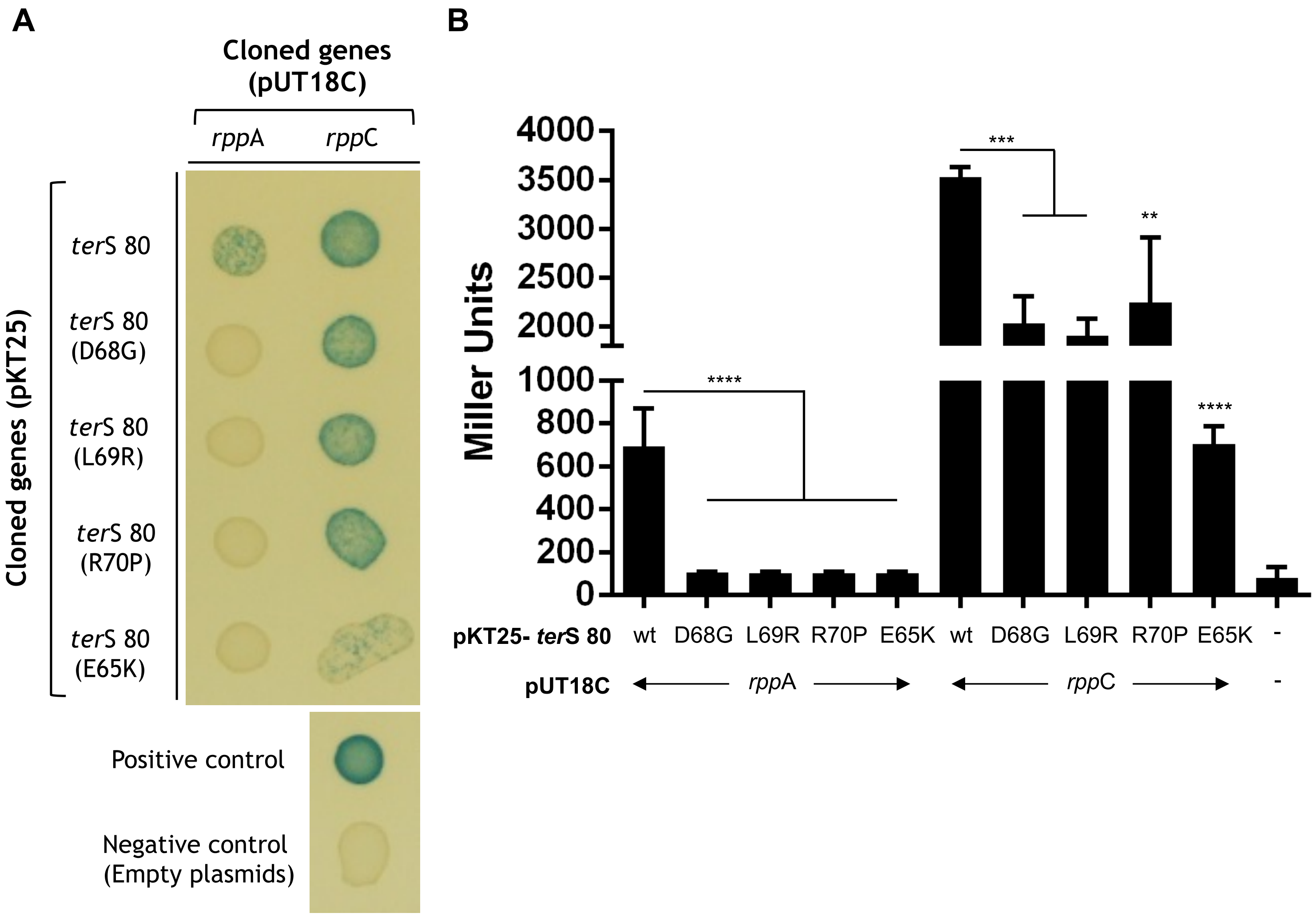

Figure S4. Characterisation of the phage 80 TerS-Rpp interaction. Related to Figures 2 and 3.
(A) BACTH analysis was performed using the plasmid pKT25 encoding different phage 80 *ter*S versions (wt, D68G, L69R, R70P and E65K) and plasmid pUT18C encoding *rpp*A or *rpp*C. Different plasmid combinations are indicated.
(B) Quantification of the BACTH analysis after overnight induction with 0.5mM IPTG. The means of results and standard deviation from three independent experiments are presented (n=3). A 1-way ANOVA with Dunnett's multiple comparisons test was performed to compare mean differences between empty plasmids. Adjusted *p* values were as follows: *rpp*C-*ter*S 80 wt vs *rpp*C-*ter*S 80 D68G p=0.0010***, *rpp*C-*ter*S 80 wt vs *rpp*C-*ter*S 80 L69R p=0.0005***, *rpp*C-*ter*S 80 wt vs *rpp*C-*ter*S 80 R70P p=0.0029**, p<0.0001****.

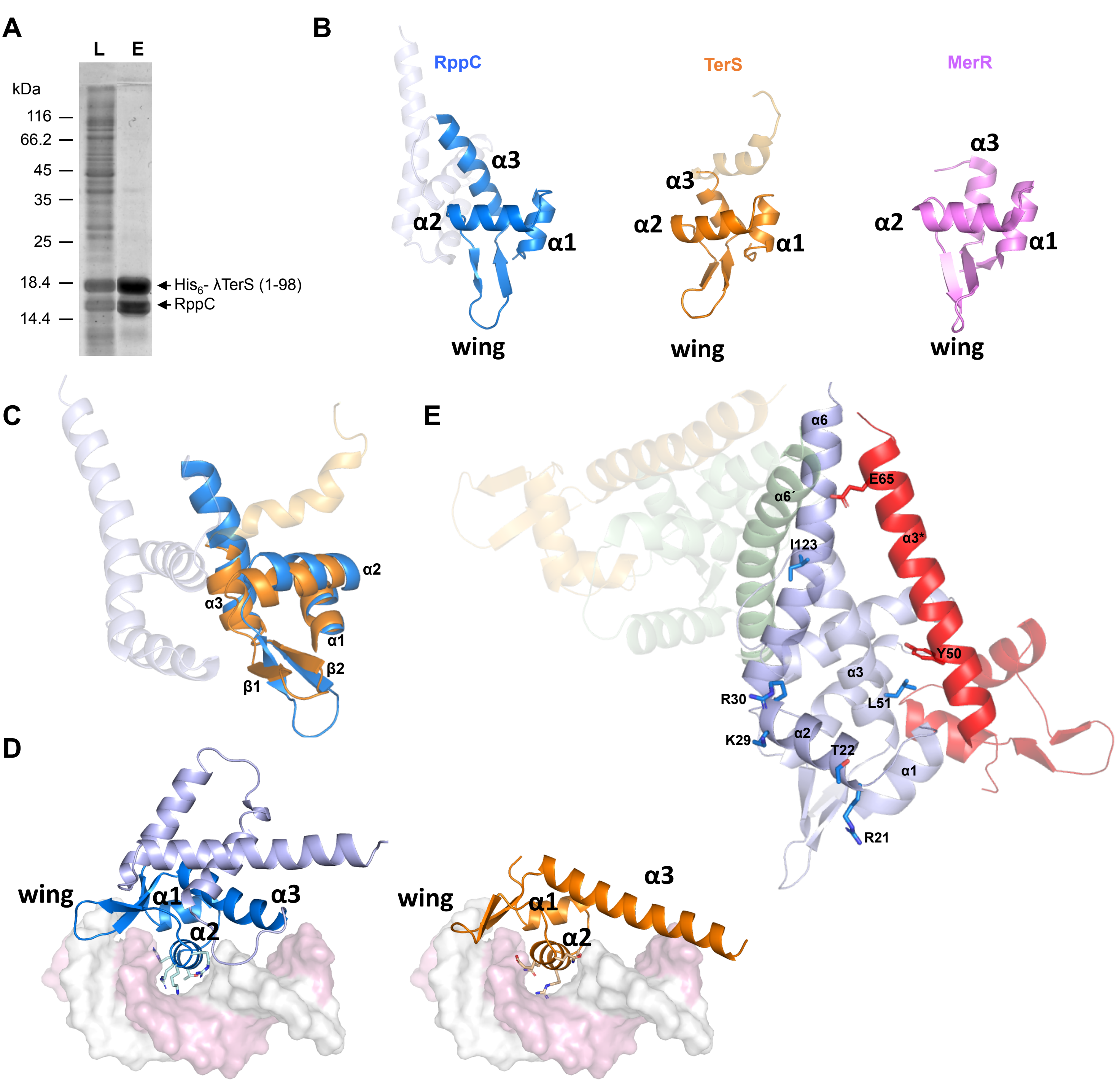

Figure S5. Characterisation of the RppA-TerS interaction. Related to Figures 2, 4, 5 and 6.

(A) RppC interacts with λ TerS (1-98). Affinity chromatography of untagged RppC using His6–λ TerS (residues 1-98). The presence of the different proteins was monitored in the load (lane L), flow-through, wash and elute (lane E) fractions by Coomassie staining.

(B) RppC DBD presents a canonical winged helix-turn-helix DNA binding domain similar to that present in the TerS DBD and in other DNA binding proteins as the MerR transcriptional regulator (PDB 6ama). The wHTH motif from each protein is highlighted in dark tone and the secondary structural elements are labelled in order from N- to C-terminus.

(C) Superimposition of the DNA binding motifs from RppC (blue) and λ TerS DBD (orange) shows identical folding.

(D) Model of RppC-DNA and λTerS-DNA complexes. Cartoon representation of RppC-DNA (left) and λTerS-DNA (right) models. RppC and $\lambda TerS^{1-98}$ are coloured in blue and orange, respectively, with the DBD highlighted in dark tone. Secondary structural elements of the DBDs are labelled in order from N- to C-terminus. The DNA binding models show a common DNA recognition and binding strategy with the helix 2 inserting into the DNA major groove and the wing in the minor groove. The RppC residues placed in these facing secondary structural elements are responsible of the specific PICI *cos*B sequence recognition (R21, T22, R25, K29, R30, from α2, represented in stick, and residues K39 and K41 from the wing), while the equivalent residues present in the λ TerS are involved in the specific recognition of the phage *cos*B site.

(E) Location of the mutated residues in the RppC-$\lambda TerS^{1-98}$ heterocomplex structure. Cartoon representation of the RppC-$\lambda TerS^{1-98}$ heterocomplex. RppC protomers are coloured in green and blue, respectively. The $\lambda TerS^{1-98}$ protomers are in red and yellow, respectively. Key secondary structural elements are labelled in order from N- to C-terminus, the apostrophe (´) indicates the second RppC molecule and the asterisk (*) the λTerS. Mutated residues are labelled and shown in stick representation, with carbon atoms coloured according to the protomer to which they belong. Nitrogen, oxygen and phosphorous atoms are coloured in dark blue, red and orange, respectively.

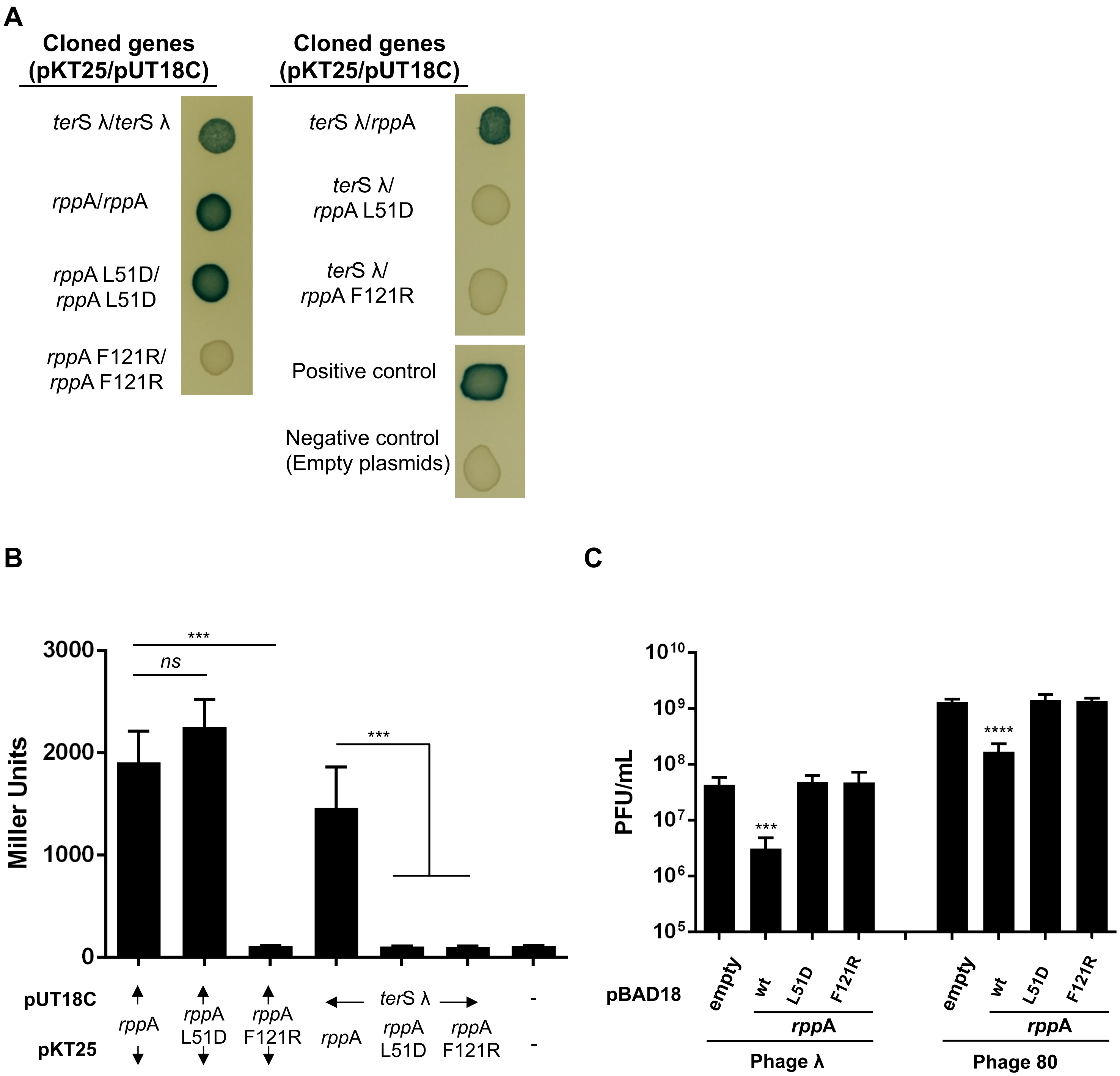

Figure S6. Characterisation of the RppA L51D and F121R mutants. Related to Figures 3 and 5.
(A) BACTH analysis was performed using derivative plasmids pKT25 and pUT18C expressing either the λ TerS or the different RppA mutants. Different plasmid combinations are indicated.
(B) Quantification of the BACTH analysis after overnight induction with 0.5mM IPTG. The means of results and standard deviation from three independent experiments are presented (n=3). A 1-way ANOVA with Dunnett's multiple comparisons test was performed to compare mean differences between *rpp*A and *rpp*A versions or *ter*S λ-*rpp*A and *ter*S λ-different *rpp*A versions. Adjusted *p* values were as follows: *rpp*A-*rpp*A vs *rpp*A F121R-*rpp*A F121R p=0.0002***, *ter*S-*rpp*A vs *ter*S-*rpp*A L51D/F121R p=0.0007***. *ns*, not significant.
(C) The RppA mutants do not block phage reproduction. *E. coli* strains expressing different RppA mutant proteins were infected with phages λ 80 and plated on phage base agar plates supplemented with 0.1% arabinose using phage top agar. Plates were incubated for 24 h at 37°C, and the number of phage plaques quantified. The means of results and standard deviation from three independent experiments are presented (n=3). A 1-way ANOVA with Dunnett's multiple comparisons test was performed to compare mean differences between pBAD18 empty row. Adjusted *p* values were as follows: p=0.0003***, p<0.0001****.

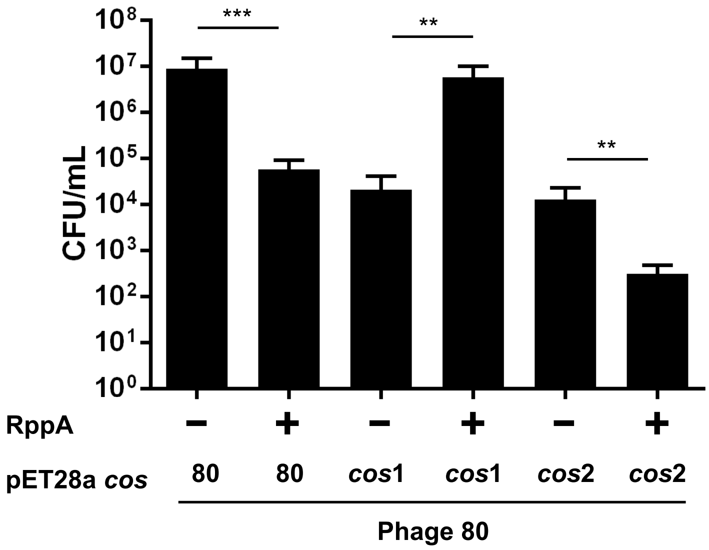

Figure S7. RppA promotes EcCICFT073 *cos*1 recognition. Related to Figure 6.
pET28a derivative plasmids, containing different *cos* sequences (80, *cos*1 or *cos*2) were introduced into the lysogenic strains for phage 80. The strains were MC induced (2 µg/ml) and the transfer of the plasmids analysed in presence or absence of RppA. Expression of RppA from plasmid pBAD18-15A was induced using 0.02% arabinose when appropriate. The figure shows the number of transductants/mL of lysate, using *E. coli* WG5 as recipient strain. The means of results and standard deviation from three independent experiments are presented (n=3). An unpaired *t*-test was performed to compare mean differences of each pET28a *cos* plasmid in presence (+) or absence (-) or *rpp*A. Adjusted *p* values were as follows: Phage 80 pET28a *cos* 80 (+) vs (-) p=0.0006***, Phage 80 pET28a *cos*1 (+) vs (-) p=0.0014**, Phage 80 pET28a *cos*2 (+) vs (-) p=0.0033**.

A

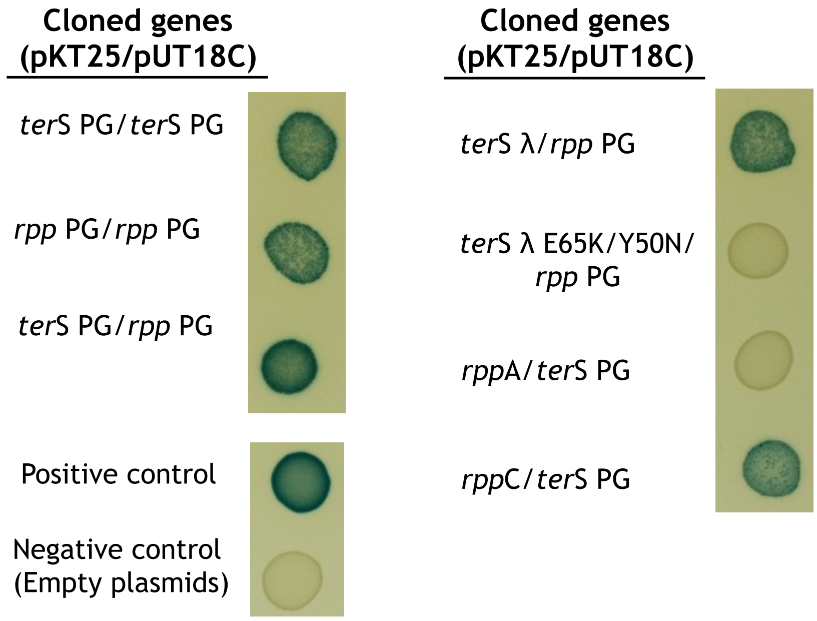

B

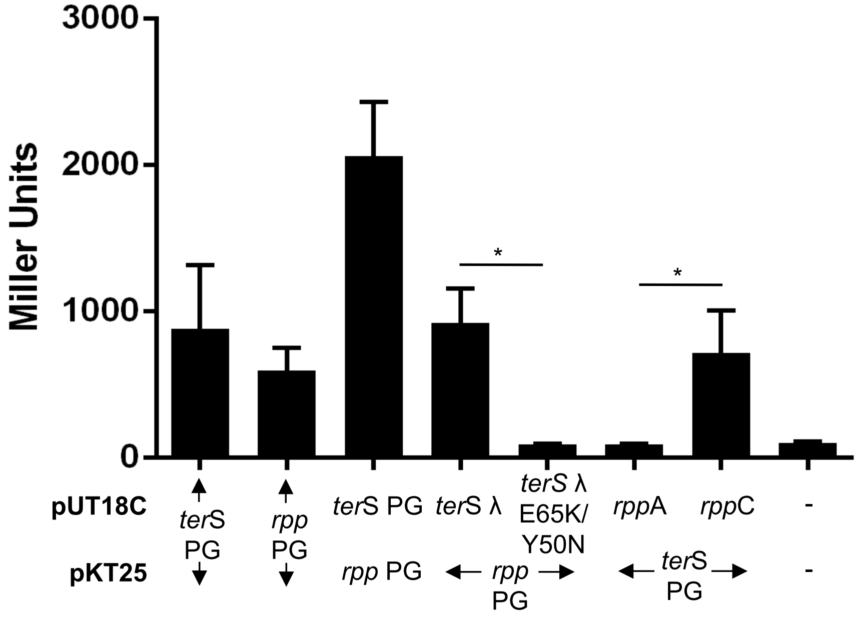

C

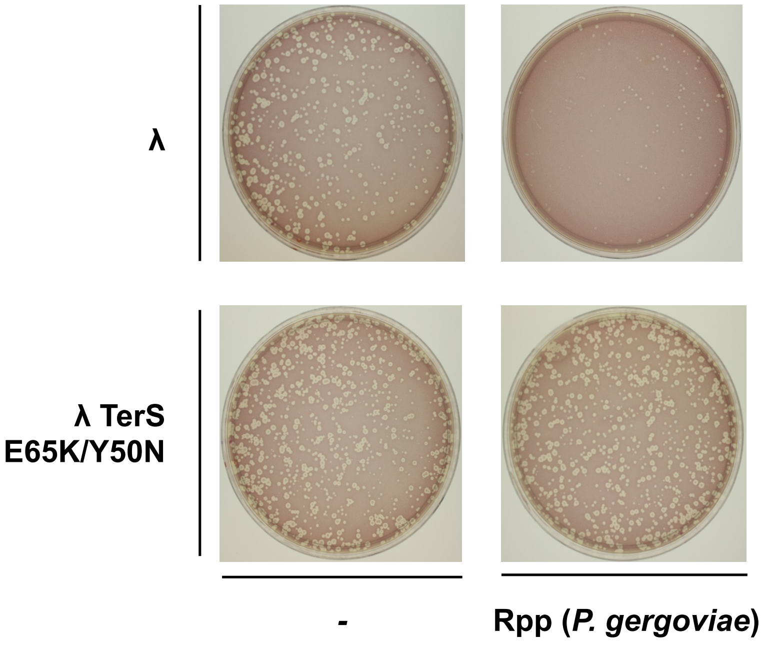

Figure S8. Testing the Rpp-TerS interaction in *Pluralibacter gergoviae*. Related to Figures 2 and 5.

(A) BACTH analysis was performed using the plasmids pKT25 and pUT18C encoding *P. gergoviae* *ter*S (KMK30155.1) and *rpp* (WP_086499225) or *E. coli* *ter*S λ, *rpp*A or *rpp*C. Different plasmid combinations are indicated.

(B) Quantification of the BACTH analysis after overnight induction with 0.5mM of IPTG. The means of results and standard deviation from three independent experiments are presented (n=3). An unpaired t-test was performed to compare mean differences within rows. Adjusted *p* values were as follows: *ter*S λ-*rpp* PG vs *ter*S λ E65K/Y50N-*rpp* PG p=0.0108*, *rpp*A-*ter*S PG vs *rpp*C-*ter*S PG p=0.0204*.

(C) Rpp from *Pluralibacter gergoviae* interferes with λ reproduction. *E. coli* strains JP13131 (594 pBAD18 empty) or JP18876 (594 pBAD18 *rpp* PG) were infected with phage λ, plated on phage bottom agar and incubated for 24 h at 37 °C. Plates were stained with 0.1% (w/v) TTC in LB and photographed.

Table S1. Identification of Rpp proteins in Gram-negative PICIs. Related to Figures 1 and S1.

Specie	Strain	Accession number
E. coli	CFT073	AAN79972.1[a]
E. coli	K1516	EZB64746.1
E. coli	O42	CBG34579.1[a]
E. coli	upec-187	WP_033562019
E. coli	EC2733.1	WP_072135240.1[b]
Shigella boydii	Sb277	ABB66699.1
Shigella dysenteriae	DMB SH20201	RIE73627.1
Shigella flexneri	CDC 796-83 SGF	EFW59515.1
Shigella sonnei	sh1475	SJK45406.1
Salmonella enterica	BCW_2636	WP_080171664.1
Pluralibacter gergoviae	MGH173	OUF48099.1

[a]The protein deposited in the GenBank lacks the first 31 residues.
[b]The protein deposited in the GenBank lacks the first 64 residues.

Table S2. Intersubunit interactions in the RppC dimer. Related to Figure 4.

Homodimer RppC						
RppC subunit A			RppC subunit B			
Structural element	Residue	Atom type	Structural element	Residue	Atom type	Distance
α3	63(LEU)	CA	α6	133(ARG)	CD	3.84
		C				3.66
		CD2			CZ	3.77
	64(GLY)	C		129(LEU)	CD2	3.64
		O		132(LYS)	NZ	2.62
α6	111(ASP)	CB		114(ALA)	CB	3.92
	114(ALA)	C		115(GLN)	CA	3.90
		CB		111(ASP)	CB	3.92
	115(GLN)	CA		114(ALA)	C	3.90
		CG		118(GLY)	CA	3.96
	118(GLY)	CA		115(GLN)	CG	3.96
				118(GLY)	C	3.97
		C				3.39
					CA	3.97
				119(ARG)		3.75
	119(ARG)	CA		118(GLY)	C	3.75
		CG		121(GLN)	CG	3.84
	121(GLN)	CG		119(ARG)	CG	3.84
	122(GLY)	CA		123(ILE)	CG1	3.91
		C		122(GLY)	C	3.53
	123(ILE)	CG1			CA	3.91
				125(ILE)	CG2	3.94
	125(ILE)	CG2		123(ILE)	CG1	3.94
	126(GLY)	C		126(GLY)	C	3.96
	129(LEU)	CD2		64(GLY)	C	3.64
	130(THR)	CG2		130(THR)	CG2	3.85
				131(LEU)	CD2	3.80
	131(LEU)	CD2		130(THR)	CG2	3.80
				134(LEU)	CD1	3.74
	132(LYS)	NZ	α3	64(GLY)	O	2.62
	133(ARG)	CD		63(LEU)	CA	3.84
					C	3.66
		CZ			CD2	3.77
	134(LEU)	CD1	α6	134(LEU)	CD1	3.55
				131(LEU)	CD2	3.74

Table S3. Intermolecular interactions in the RppC-TerS^{Nter} complex. Related to Figure 5.

Heterocomplex RppC-TerS						
RppC			TerS			
**Structural element**	**Residue**	**Atom type**	**Structural element**	**Residue**	**Atom type**	**Distance**
**α1**	6(PHE)	CD1	**Lα1**	1(MET)	CE	3.88
		CZ				3.97
				3(VAL)	CG1	3.53
			**Lα1-α2**	11(ILE)	CD1	3.74
			**α1**	7(GLN)	CD	3.94
		CE2			CB	3.52
					CG	3.74
					CD	3.98
				11(ILE)	CD1	3.36
					CG1	3.65
		CB				3.97
		CG				3.60
					CD1	3.83
		CD2				3.40
					CG1	3.16
	16(GLN)	NE2	**α3**	43(SER)	OG	2.62
**α2**	47(LEU)	CB	**α1**	11(ILE)	CG2	3.64
					CG	3.57
					CD2	3.74
	51(LEU)	CD2		12(PHE)	CE1	3.61
			**α3**	50(TYR)	CD2	3.72
		CD1				3.90
	52(PHE)	CE1			CZ	3.92
	55(ARG)	CD		51(ALA)	CB	3.80
		NE		54(ASP)	OD1	3.00
	63(LEU)	CD1		58(GLU)	CD	3.78
**α3**	77(HIS)	CE1			CD	3.77
	81(ILE)	CG2		57(ILE)	CG2	3.89
		CD1		61(LYS)	CE	3.91
	82(MET)	CE		22(TRP)	CH2	3.98
	85(MET)	O			NE1	2.72
		CG			CZ2	3.76
		CE		53(ARG)	CG	3.02
					CD	3.62
				57(ILE)	CD1	2.28
		CB		53(ARG)	CZ	3.60
**α4**	95(TYR)	CE1	**α2**	14(ALA)	C	3.67
				15(SER)	CA	3.52
		CE2			CB	3.82
		CZ			CA	3.52
					CB	3.55
**α6**	127(ARG)	NH2	**α3**	61(LYS)	O	2.82
				65(GLU)	OE1	2.97
	133´(ARG)	NE			OE1	2.85
		NH1			OE1	2.65

L = loop

Table S4. Strains used in this study. Related to STAR Methods.

Strain	Description	Reference
594	Laboratory strain	
C600	Laboratory strain	ATCC 23738
WG5	Laboratory strain	
DH5α	Laboratory strain	
BTH101	Bacterial Adenylate CyclaseTwo-hybrid System Kit	Euromedex
BL21 (DE3)	Protein overexpression	Novagen
JP10400	C600 phage lambda lysogen	(Fillol-Salom et al., 2018)
JP12507	594 phage 80 lysogen	(Fillol-Salom et al., 2018)
JP12508	594 phage HK97 lysogen	This work
JP13131	594 pBAD18	This work
JP19328	594 pJP2214	This work
JP19329	594 pJP2215	This work
JP19330	594 pJP2216	This work
JP13132	594 pJP2217	This work
JP13133	594 pJP2218	This work
JP13134	594 pJP2219	This work
JP13135	594 pJP2220	This work
JP13136	594 pJP2221	This work
JP13137	594 pJP2222	This work
JP12677	C600 EcCICFT073-c1501::*tet*A	(Fillol-Salom et al., 2018)
JP13957	C600 EcCICFT073-c1501::*tet*A Δc1503	This work
JP16526	594 pJP2243	This work
JP16528	594 pJP2304	This work
JP12979	C600 lambda evolved-EcCICFT073::*tet*A (*nu*1-V3I)	This work
JP12982	C600 lambda evolved-EcCICFT073::*tet*A (*nu*1-A55V)	This work
JP12993	C600 lambda evolved-pBAD18 *rpp*A (*nu*1-E65K)	This work
JP16575	JP12993 evolved-pBAD18 *rpp*C (*nu*1-E65K/Y50N)	This work
JP13173	594 phage 80 evolved-pBAD18 *rpp*A (1) (gp01-D68G)	This work
JP17583	594 phage 80 evolved-pBAD18 *rpp*A (2) (gp01-L69R)	This work
JP17545	594 phage 80 evolved-pBAD18 *rpp*A (3) (gp01-R70P)	This work
JP16571	594 phage 80 evolved-pBAD18 *rpp*C (gp01-E65K)	This work
JP15009	JP10400 pBAD18	This work
JP15012	JP10400 pJP2218	This work
JP15013	JP12507 pBAD18	This work
JP15016	JP12507 pJP2218	This work
JP19363	BTH101 pJP2225 pJP2224	This work
JP19364	BTH101 pJP2228 pJP2224	This work
JP19365	BTH101 pJP2262 pJP2224	This work
JP19366	BTH101 pJP2225 pJP2244	This work
JP19367	BTH101 pJP2228 pJP2244	This work
JP19368	BTH101 pJP2262 pJP2244	This work
JP19369	BTH101 pKT25-control pUT18C-control	This work
JP19370	BTH101 pKT25 pUT18C	This work
JP19388	BTH101 pJP2225 pJP2250	This work
JP19389	BTH101 pJP2249 pJP2224	This work
JP19390	BTH101 pJP2245 pJP2244	This work
JP19391	BTH101 pJP2249 pJP2244	This work
JP19371	BTH101 pJP2229 pJP2224	This work
JP19372	BTH101 pJP2230 pJP2224	This work
JP19373	BTH101 pJP2231 pJP2224	This work
JP19374	BTH101 pJP2232 pJP2224	This work

Strain	Description	Reference
JP19375	BTH101 pJP2305 pJP2224	This work
JP19376	BTH101 pJP2229 pJP2244	This work
JP19377	BTH101 pJP2230 pJP2244	This work
JP19378	BTH101 pJP2231 pJP2244	This work
JP19379	BTH101 pJP2232 pJP2244	This work
JP19380	BTH101 pJP2305 pJP2244	This work
JP13413	JP10400 EcCICFT073-c1504-07::*cat*	This work
JP15342	JP10400 EcCICFT073-c1504-07::*cat* c1503*	This work
JP13891	JP12507 EcCICFT073-c1504-07::*cat*	This work
JP15377	JP12507 EcCICFT073-c1504-07::*cat* c1503*	This work
JP15293	JP12979 EcCICFT073-c1504-07::*cat*	This work
JP15294	JP12993 EcCICFT073-c1504-07::*cat*	This work
JP15295	JP12982 EcCICFT073-c1504-07::*cat*	This work
JP15325	JP13173 EcCICFT073-c1504-07::*cat*	This work
JP17617	JP17583 EcCICFT073-c1504-07::*cat*	This work
JP17618	JP17545 EcCICFT073-c1504-07::*cat*	This work
JP19394	BTH101 pJP2258 pJP2255	This work
JP19395	BTH101 pJP2259 pJP2256	This work
JP19397	BTH101 pJP2225 pJP2255	This work
JP19398	BTH101 pJP2225 pJP2256	This work
JP19332	594 pJP2252	This work
JP19333	594 pJP2253	This work
JP15839	JP10400 EcCICFT073-c1504-07::*cat* c1503 L51D	This work
JP18258	JP10400 EcCICFT073-c1504-07::*cat* c1503 F121R	This work
JP15961	JP12507 EcCICFT073-c1504-07::*cat* c1503 L51D	This work
JP18084	JP12507 EcCICFT073-c1504-07::*cat* c1503 F121R	This work
JP15994	JP10400 pJP2030 pJP2233	This work
JP15995	JP10400 pJP2030 pJP2234	This work
JP15996	JP10400 pJP2033 pJP2233	This work
JP15997	JP10400 pJP2033 pJP2234	This work
JP15998	JP10400 pJP2034 pJP2233	This work
JP15999	JP10400 pJP2034 pJP2234	This work
JP16012	JP12507 pJP2031 pJP2233	This work
JP16013	JP12507 pJP2031 pJP2234	This work
JP16014	JP12507 pJP2033 pJP2233	This work
JP16015	JP12507 pJP2033 pJP2234	This work
JP16016	JP12507 pJP2034 pJP2233	This work
JP16017	JP12507 pJP2034 pJP2234	This work
JP16000	JP10400 pJP2035 pJP2233	This work
JP16001	JP10400 pJP2035 pJP2234	This work
JP16002	JP10400 pJP2036 pJP2233	This work
JP16003	JP10400 pJP2036 pJP2234	This work
JP19697	JP10400 pJP2033 pJP2311	This work
JP16578	594 phage 80 orf63-orf64:*cat* chimera *cos*B:*cos*1 EcCICFT073 pBAD18	This work
JP16579	594 phage 80 orf63-orf64:*cat* chimera *cos*B:*cos*1 EcCICFT073 pJP2218	This work
JP19401	BTH101 pJP2306 pJP2308	This work
JP19402	BTH101 pJP2306 pJP2309	This work
JP19404	BTH101 pJP2307 pJP2309	This work
JP19405	BTH101 pJP2225 pJP2309	This work
JP19406	BTH101 pJP2262 pJP2309	This work
JP19407	BTH101 pJP2249 pJP2308	This work

Strain	Description	Reference
JP19408	BTH101 pJP2245 pJP2308	This work
JP18876	594 pJP2310	This work

Table S6. Plasmids used in this study. Related to STAR Methods.

Plasmid	Description	Reference
**pET28a**	Km^R. Expression vector	Novagen
**pProEX HTa**	Amp^R. Expression vector	Life Technologies
**pKD46**	Amp^R. Thermosensitive plasmid with Red system of lambda phage	(Datsenko and Wanner, 2000)
**pCP20**	Amp^R. Thermosensitive plasmid with Red system of lambda phage	(Datsenko and Wanner, 2000)
**pWRG717**	Amp^R, *km*R. pBluescript II SK+ derivative, *aph* resistance cassette and I-*Sce*I cleavage site.	(Hoffmann et al., 2017)
**pWRG99**	Amp^R. Thermosensitive plasmid with Red system of lambda phage and I-*Sce*I endonuclease under control of tetracycline-inducible promoter (P_{tetA})	(Blank et al., 2011)
**pUT18C**	Amp^R. Bacterial Adenylate CyclaseTwo-hybrid System Kit	Euromedex
**pKT25**	Km^R. Bacterial Adenylate CyclaseTwo-hybrid System Kit	Euromedex
**pUT18C-control**	Amp^R. Bacterial Adenylate CyclaseTwo-hybrid System Kit	Euromedex
**pKT25-control**	Km^R. Bacterial Adenylate CyclaseTwo-hybrid System Kit	Euromedex
**pBAD18**	Amp^R. Expression vector	(Guzman et al., 1995)
**pJP2233**	pBAD18 derivative, origin of replication 15A. Expression vector. Amp^R	This work
**pJP2214**	pBAD18 c1499 EcCICFT073	This work
**pJP2215**	pBAD18 c1500 EcCICFT073	This work
**pJP2216**	pBAD18 c1501 EcCICFT073	This work
**pJP2217**	pBAD18 c1502 EcCICFT073	This work
**pJP2218**	pBAD18 c1503 EcCICFT073	This work
**pJP2219**	pBAD18 c1504 EcCICFT073	This work
**pJP2220**	pBAD18 c1505 EcCICFT073	This work
**pJP2221**	pBAD18 c1506 EcCICFT073	This work
**pJP2222**	pBAD18 c1507 EcCICFT073	This work
**pJP2304**	pBAD18 *rpp*B	This work
**pJP2243**	pBAD18 *rpp*C	This work
**pJP2225**	pKT25 *ter*S lambda	This work
**pJP2228**	pKT25 *ter*S lambda E65K	This work
**pJP2262**	pKT25 *ter*S lambda E65K/Y50N	This work
**pJP2224**	pUT18C *rpp*A	This work
**pJP2244**	pUT18C *rpp*C	This work
**pJP2249**	pKT25 *rpp*A	This work
**pJP2245**	pKT25 *rpp*C	This work
**pJP2250**	pUT18C *ter*S lambda	This work
**pJP2229**	pKT25 *ter*S phage 80	This work
**pJP2230**	pKT25 *ter*S phage 80 D68G	This work
**pJP2231**	pKT25 *ter*S phage 80 L69R	This work
**pJP2232**	pKT25 *ter*S phage 80 R70P	This work
**pJP2305**	pKT25 *ter*S phage 80 E65K	This work
**pJP2285**	pProEX HTa *rpp*C	This work
**pJP2286**	pProEX HTa *ter*S lambda(1-98) *rpp*C	This work
**pJP2255**	pUT18C *rpp*A L51D	This work

Plasmid	Description	Reference
**pJP2256**	pUT18C *rpp*A F121R	This work
**pJP2258**	pKT25 *rpp*A L51D	This work
**pJP2259**	pKT25 *rpp*A F121R	This work
**pJP2252**	pBAD18 *rpp*A L51D	This work
**pJP2253**	pBAD18 *rpp*A F121R	This work
**pJP2234**	pBAD18 15A *rpp*A	This work
**pJP2030**	pET28a *cos* lambda	(Fillol-Salom A., 2018)
**pJP2031**	pET28a *cos* phage 80	(Fillol-Salom A., 2018)
**pJP2033**	pET28a *cos*1 EcCICFT073	(Fillol-Salom A., 2018)
**pJP2034**	pET28a *cos*2 EcCICFT073	(Fillol-Salom A., 2018)
**pJP2035**	pET28a *cos*1-*cos*B *cos*2 EcCICFT073	This work
**pJP2036**	pET28a *cos*2-*cos*B *cos*1 EcCICFT073	This work
**pJP2311**	pBAD18 15A *rpp*A R21A T22A	This work
**pJP2306**	pKT25 *ter*S *Pluralibacter gergoviae*	This work
**pJP2307**	pKT25 *rpp Pluralibacter gergoviae*	This work
**pJP2308**	pUT18C *ter*S *Pluralibacter gergoviae*	This work
**pJP2309**	pUT18C *rpp Pluralibacter gergoviae*	This work
**pJP2310**	pBAD18 *rpp Pluralibacter gergoviae*	This work
